# Investigating unmet need for healthcare using the European Health Interview Survey: a cross-sectional survey study of Luxembourg

**DOI:** 10.1136/bmjopen-2021-048860

**Published:** 2021-08-03

**Authors:** Valerie Moran, Marc Suhrcke, Maria Ruiz-Castell, Jessica Barré, Laetitia Huiart

**Affiliations:** 1 Department of Population Health, Luxembourg Institute of Health, Strassen, Luxembourg; 2 Living Conditions department, Luxembourg Institute of Socio-Economic Research, Esch-sur-Alzette/Belval, Luxembourg; 3 Centre for Health Economics, University of York, York, UK; 4 Health and Health Systems, Luxembourg Institute of Socio-Economic Research, Esch-sur-Alzette/Belval, Luxembourg; 5 Service Nomenclature, conventions, analyse et prospective, Caisse nationale de santé, Luxembourg, Luxembourg; 6 Direction générale, Santé publique France, Saint-Maurice, France

**Keywords:** health economics, health policy, international health services, organisation of health services

## Abstract

**Objectives:**

We investigate the prevalence of unmet need arising from wait times, distance/transportation and financial affordability using the European Health Interview Survey. We explore associations between individual characteristics and the probability of reporting unmet need.

**Design:**

Cross-sectional survey conducted between February and December 2014.

**Setting and participants:**

4004 members of the resident population in private households registered with the health insurance fund in Luxembourg aged 15 years and over.

**Outcome measures:**

Six binary variables that measured unmet need arising from wait time, distance/transportation and affordability of medical, dental and mental healthcare and prescribed medicines among those who reported a need for care.

**Results:**

The most common barrier to access arose from wait times (32%) and the least common from distance/transportation (4%). Dental care (12%) was most often reported as unaffordable, followed by prescribed medicines (6%), medical (5%) and mental health (5%) care. Respondents who reported bad/very bad health were associated with a higher risk of unmet need compared with those with good/very good health (wait: OR 2.41, 95% CI 1.53 to 3.80, distance/transportation: OR 7.12, 95% CI 2.91 to 17.44, afford medical care: OR 5.35, 95% CI 2.39 to 11.95, afford dental care: OR 3.26, 95% CI 1.86 to 5.71, afford prescribed medicines: OR 2.22, 95% CI 1.04 to 4.71, afford mental healthcare: OR 3.58, 95% CI 1.25 to 10.30). Income between the fourth and fifth quintiles was associated with a lower risk of unmet need for dental care (OR 0.29, 95% CI 0.16 to 0.53), prescribed medicines (OR 0.38, 95% CI 0.17 to 0.82) and mental healthcare (OR 0.17, 95% CI 0.05 to 0.61) compared with income between the first and second quintiles.

**Conclusions:**

Recent and planned reforms to address waiting times and financial barriers to accessing healthcare may help to address unmet need. In addition, policy-makers should consider additional policies targeted at high-risk groups with poor health and low incomes.

**Strengths and limitations of this study**

- We investigated unmet need using a representative cross-sectional population based survey—the European Health Interview Survey (EHIS).
- The EHIS allowed us to investigate unmet need due to the affordability of prescribed medications and mental healthcare, in addition to medical and dental care.
- As unmet need was self-reported, it may be subject to recall bias or response bias.
- The EHIS does not collect data on unmet need due to wait for different types of services.
- This study explored associations between unmet need and sociodemographic, health status and risk factor variables but did not establish causality.

## Introduction

In recent years, the concept of universal health coverage (UHC) has emerged as a key objective of international organisations including the WHO, Organisation for Economic Co-Operation and Development (OECD) and World Bank, who support national governments to achieve this goal. UHC is defined as ‘ensuring that all people have access to needed health services…of sufficient quality to be effective while also ensuring that the use of these services does not expose the user to financial hardship’.^1^ Therefore, access to care is a core component of UHC. At the European Union (EU) level, the European Parliament, Council and Commission announced the European Pillar of Social Rights in 2017.^2^ The Pillar comprises 20 guiding principles that underpin a fair and inclusive society. Chapter 3 of the Pillar relates to social protection and inclusion and contains the principle that everyone is entitled to timely access to good quality affordable healthcare.^3^ Barriers to accessing healthcare arising from the cost, physical accessibility and quality of services can lead to unmet need at an individual level due to affordability, distance and waiting times.^4 5^ Unmet need may also arise from other factors related to personal choices and circumstances including a lack of time to seek care due to family or work responsibilities, fear or dislike of medical personnel and treatment, a preference to wait and see if the symptoms resolve by themselves without seeking care and issues related to health literacy including language problems and a lack of knowledge of appropriate medical care.^6 7^

Countries have introduced various policies to address different aspects of unmet need. Many countries have implemented maximum waiting times, particularly for specialist consultations and elective treatments.^8^ Countries have also introduced policies to reduce financial barriers to accessing healthcare. Belgium has put in place a range of financial protection measures including lower copayments for vulnerable groups and ceilings on the total amount of copayments paid by a household based on household income, regulation of supplementary payments and the third-party payment system (outlined in more detail below).^9^ In France, individuals with chronic illnesses, pregnant women, low-income groups and individuals who suffered a work accident are exempt from most or all copayments.^10 11^

### Previous literature on unmet need for healthcare

Survey data are commonly used to ascertain individuals’ perceptions of unmet need arising from various barriers to accessing care.^12 13^ To date, studies that investigated unmet need within and across European countries have used data from the European Union Statistics on Income and Living Conditions (EU-SILC),^14–25^ the European Social Survey (ESS)^26 27^ and the European Health Interview Survey (EHIS).^28–30^ Several studies that investigated unmet need for medical care^14 16 18 19 21 27^ found that females were associated with higher unmet need compared with males. While there were conflicting results across studies for variables measuring age, education, employment, immigrant status and urban vs rural area, there was consensus for other covariates. Respondents in poorer health^14 16 23 25–27^ and those with a chronic condition or illness^14 16 25 27^ were more likely to report unmet need. Higher income groups were less likely to report unmet need^14 16 23 25 27^ as were respondents with greater social capital and social support.^19 27^ A small number of studies examined the determinants of unmet need for different services. Chaupain-Guillot and Guillot^15^ found that older age was associated with a lower probability of reporting unmet need for medical and dental care while poorer health status and lower income was associated with a higher probability. However, while a higher level of education was associated with an increased probability of reporting unmet need for medical care, it was associated with a reduced probability of reporting unmet need for dental care. Hoebel *et al*^28^ investigated unmet need among older people with low socioeconomic status in Germany. Among those aged 50–64, low socioeconomic status was associated with higher unmet need for medical, dental and mental healthcare for both men and women. However, among the 65–85 age group low socioeconomic status was associated with higher unmet need only among men for medical and dental care. Rotarou and Sakellariou and Sakellariou and Rotarou^29 30^ found that people with a disability were more likely to report higher unmet need for medical, dental and mental healthcare in Greece and the UK, respectively.

### The Luxembourgish health system and access to healthcare

Luxembourg provides universal coverage of healthcare through a mandatory social health insurance system, the Caisse Nationale de Santé (CNS). In 2018, the CNS covered 93% of the resident population.^31 32^ The proportion not covered included EU officials based in Luxembourg, who were insured under a separate scheme provided by their employer^33^ and vulnerable populations including the homeless and irregular immigrants and their families.^34^

In Luxembourg, when patients access healthcare including doctor consultations in outpatient or inpatient settings, dental and paramedical services, they must pay providers the full cost on receipt of care and then apply for a refund from the CNS for the covered share of the payment (excluding copayments). The CNS directly reimburses providers for hospital services (excluding the doctors’ fees), laboratory tests and pharmaceuticals, leaving the patient to pay only the copayment at the point of use.^35^

Luxembourg is among a minority of countries (including Belgium and France) in the EU where patients pay ambulatory care providers directly and then claim reimbursement from the social health insurance fund.^36^ The payment of the full cost of medical, dental and mental healthcare by the patient on receipt of care may create a financial barrier to accessing care for certain groups, for example, those on lower incomes.^34^ In Belgium and France, specific population groups including those on low incomes or with a chronic illness have their costs directly covered by the social insurance fund, a scheme known as the ‘tiers payant*’* or third-party payment system.^10 37^ In Luxembourg, the government introduced the system of ‘third-party social payment’ (‘tiers payant social’) in 2013, which entitles people in economic hardship to request assistance with the payment of healthcare expenses.^38^ Eligible patients are exempted from the payment of costs and the CNS reimburses the provider. The local social welfare office covers any copayments the patient cannot afford. The purpose of this policy is to enable people in economic hardship to access healthcare.^38^ Therefore, the third-party payment system addresses unmet need arising from financial barriers to care in order to reduce financial hardship arising from healthcare utilisation.^9^ However, it is unclear what the impact of this policy has been on addressing unmet need due to affordability of care in Luxembourg, as the policy has not been evaluated to date. Nevertheless, following a national debate over recent years, the government announced in November 2019 that a universal system of third-party payment would be introduced,^39^ which would cover the entire enrolled population of the CNS and replace the existing third-party social payment.

In addition to financial access to care, waiting times for health services have been identified as an important policy issue in Luxembourg. Waiting times are commonly a pertinent issue in countries with a national health system funded by general taxation. However, a recent report highlighted that waiting times were a medium to high priority in Luxembourg, in contrast to its neighbouring countries of Belgium and Germany, where waiting times were a low to medium or low priority.^8^ This report also revealed that waiting times were an issue across different types of services including specialist care, diagnostic tests, hospital emergency departments, primary care and cancer care. Data on waiting times for healthcare in Luxembourg are not routinely published. However, a 2016 study revealed an average wait of almost 4 hours between admission to and discharge from emergency services, which are provided by each of the four general hospitals. The majority (75%) of attendees experienced a wait of 3 hours or less, just below the government target of 85%.^40^ These findings prompted the government to set a maximum waiting time target of 2.5 hours for emergency services.^41^ Efforts have also been undertaken to improve the organisation of cancer care in order to reduce waiting times. In 2016, the government embarked on a reform of the National Health Laboratory’s diagnostic services by reducing outsourcing to other countries and concentrating the delivery of these services in Luxembourgish hospitals instead. The government also introduced maximum waiting time targets for cancer care. At least 95% of patients should receive a diagnosis within five working days while specific targets are in place for different types of cancer (eg, a maximum of 4 weeks between chemotherapy and radiotherapy or 2 weeks following receipt of the analytical report).^8^ If residents of Luxembourg enrolled in the CNS perceive wait times as too long, they may seek healthcare in another EU state. Prior authorisation from the CNS is not required for a doctor consultation (in a health centre, clinic or hospital) but is required if the consultation uses specialised medical equipment or hospital infrastructure or for inpatient treatments with at least one overnight stay. The CNS may withhold authorisation if the necessary treatment can be provided in Luxembourg within a medically justifiable time frame.^42^

The issue of unmet need for healthcare in Luxembourg has not been studied to date despite its policy relevance and the availability of relevant survey data. The objective of this paper is to investigate the prevalence and determinants of unmet need in Luxembourg. We used EHIS data as it allowed us to explore the prevalence and determinants of unmet need separately for waiting time, distance or transportation and the affordability of medical, dental and mental healthcare and prescribed medicines. Therefore, we also contribute to the limited number of studies that investigated unmet need not only for medical care but also for dental and mental healthcare and prescribed medications.

## Data and methods

### Study population and design

The EHIS is a cross-sectional observational survey undertaken in all EU countries. A first wave of data was collected in 17 EU member states (excluding Luxembourg) between 2006 and 2009.^43^ A second wave of the survey (EHIS 2) was collected in all 28 EU countries together with Iceland, Norway and Turkey between 2013 and 2015. Detailed information on the EHIS 2 methodology is available in a manual published by Eurostat^43^ while a paper published by the Robert Koch Institute in Germany^44^ provides a concise overview of the background and study methodology of the EHIS 2. Information on the EHIS two for Luxembourg, including the questionnaire and data access procedure is available on the Ministry of Health Directorate of Health website.^45^ The survey collected information on health status, health determinants, utilisation of and barriers to access to healthcare and sociodemographic characteristics.^46^ The coverage of the survey included the resident population in private households aged 15 years and over. A one-stage random sample stratified by age, sex and district of residence (Luxembourg, Diekirch and Grevenmacher) was drawn from the registry of CNS insurees.^45 47^ Among the 16 000 individuals invited to participate, 4823 responded (response rate of 30.1%) by submitting an electronic (70%) or paper (30%) questionnaire.^46^ Of these respondents, 4118 participants met the inclusion criteria, provided informed consent and completed the questionnaire (participation rate of 24.7%). Data were collected between February and December 2014.^47^ The EHIS 2 Luxembourg database comprised 4004 individuals who completed more than 50% of the questionnaire and had no missing data for age, sex or district, (final participation rate of 25%). This database was prepared according to a European protocol^48^ and was validated by Eurostat.^45^

The EHIS differs from the EU-SILC and ESS as it does not ask respondents a binary (yes/no) question on whether they have unmet need. Rather, the EHIS asks respondents to consider unmet need arising from specific barriers to accessing healthcare, including long waits, distance or transportation problems and the affordability of services. The EHIS data allows the investigation of each component of unmet need separately and the consideration of financial barriers for medical, dental and mental healthcare and prescribed medicines. The survey questions are available in online supplemental appendix 1. Table 1 shows the number and percentage of respondents who reported no unmet need, an unmet need or no need for healthcare for each component of unmet need. The percentage of respondents who reported no need for healthcare ranged from 15% for affordability of dental care and prescribed medicines to 38% for mental healthcare.

10.1136/bmjopen-2021-048860.supp1 Supplementary data

**Table 1:** Responses for the six components of unmet need

	Wait		Distance or transportation problems		Could not afford medical care		Could not afford dental care		Could not afford prescribed medicines		Could not afford mental healthcare	
	N	%	N	%	N	%	N	%	N	%	N	%
No	1906	48	2482	62	2866	72	2809	70	2977	74	2097	52
Yes	878	22	92	2	182	5	399	10	216	5	106	3
No need for healthcare	1043	26	983	25	782	20	599	15	609	15	1533	38
Missing	177	4	447	11	174	4	197	5	202	5	268	7
Total	4004	100	4004	100	4004	100	4004	100	4004	100	4004	100

### Outcome variables

For our outcome variables, we created six binary variables to measure barriers to access due to wait time, distance and affordability of medical care, dental care, prescribed medicines and mental healthcare among those who reported a need for care. We coded these variables as one, if respondents replied ‘yes’, as zero if respondents replied ‘no’ and as missing if respondents reported ‘no need for healthcare’.

### Explanatory variables

**Figure 1:**
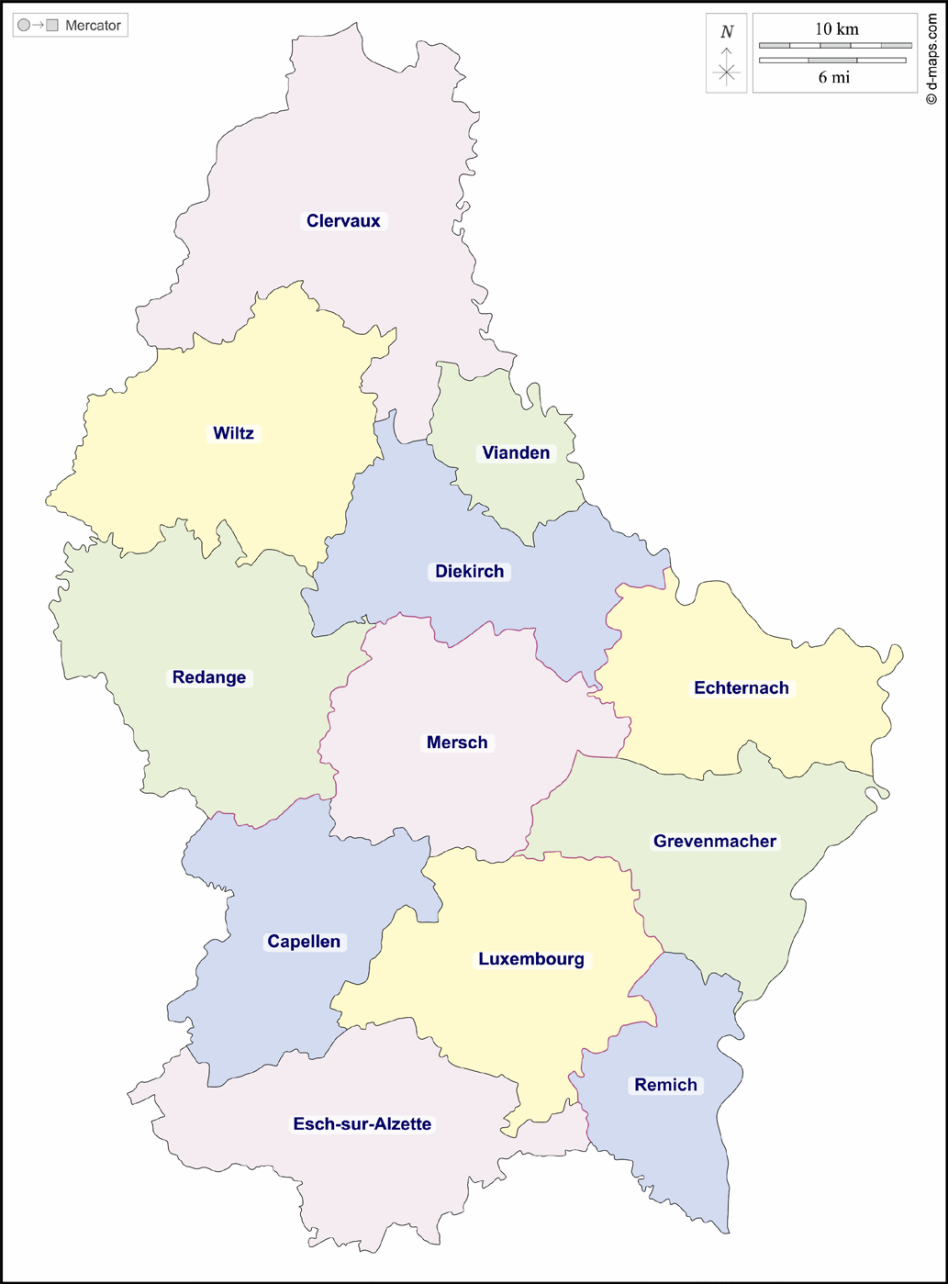
Map of Luxembourg cantons.

Explanatory variables covered sociodemographics, health behaviours and health status. Sociodemographic variables included sex, age, marital status, immigrant, education, employment status, income, social support and being an informal carer. Sex, marital status, immigrant and informal carer were constructed as binary variables while age, education and employment status were constructed as categorical variables. Household income was measured in quintiles and included as a categorical variable. Following Ruiz-Castell *et al*,^49^ we created a categorical variable on low, moderate and high social support using the questions: ‘how much concern do people show in what you are doing?’ and ‘how easy is it to get practical help from neighbours if you should need it?’. Health risk factors included body mass index (BMI), smoking and alcohol consumption. We considered alcohol consumption as ‘irregular’, if it occurred at most 2–3 days per month, and ‘regular’, if it occurred at least once per week. We used three measures of health status: self-assessed health, presence of a chronic disease and limitations in activities due to health problems. We also included binary variables (fixed effects) for the twelve cantons (see figure 1) to capture geographical differences in unmet need.

We excluded observations with missing data for the explanatory variables. Online supplemental appendix 1, table A1 shows the percentage of missing data for each explanatory variable. Missing data for the explanatory variables did not exceed 7%, except for income, where 29% of observations had missing data. Online supplemental appendix 1, table A2 shows the characteristics of respondents with missing income data. These were more likely female, aged 15–24, unmarried, of Luxembourgish nationality, had only primary and pre-primary education, were students, fulfilling domestic tasks or in compulsory service, had no limitations in activities due to a health problem and were less likely to be overweight or smoke daily.

### Statistical data analysis

We conducted separate analyses for each component of unmet need and investigated associations between the outcome and explanatory variables using multivariate logistic regression models of the following form:

$$P(y=1|x)=\frac{e^{\alpha +\beta x}}{1+e^{\left(\alpha +\beta x\right)}}$$

where y is unmet need due to wait, distance or affordability and X is a vector of explanatory variables.

We used sample weights to ensure the sample was representative of the population in terms of age, sex and district of residence. We estimated models with robust standard errors. As a sensitivity analysis, following Bataineh *et al*,^50^ we included a binary variable equal to one for observations with missing data for income in all models. We reported results for all models as ORs adjusted for the explanatory variables with 95% CI. We estimated all models using Stata V.15.^51^

### Patient and public involvement

Patients or the public were not involved in the design, conduct, reporting or dissemination of the EHIS.

## Results

Table 2 shows the sample size for each component of unmet need and the weighted percentage of unmet need. The most commonly cited barrier to access was due to wait times (32%) while the least common was distance (4%). Fifteen per cent of respondents reported unmet need due to affordability of care. Dental care (12%) was most commonly cited as being unaffordable, followed by prescribed medicines (6%), medical (5%) and mental healthcare (5%).

**Table 2:** Prevalence of unmet need

Variable	N	N, unmet need=1	%
Wait	1639	529	32
Distance or transportation problems	1531	51	4
Could not afford medical care	1813	94	5
Could not afford dental care	1888	222	12
Could not afford prescribed medicines	1899	108	6
Could not afford mental healthcare	1268	63	5

N refers to number of respondents in estimation sample, N, unmet need=1 refers to the number of respondents in the estimation sample who reported an unmet need for each component and % is the percentage of estimation sample who reported an unmet need for each component weighted for age, sex and district of residence.

Table 3 presents the number and weighted percentage of respondents according to the explanatory variables for each component of unmet need. A higher proportion of respondents who had bad or very bad health, a chronic disease, limitations in activities due to health problems or were ex-drinkers reported unmet need for all components. A higher proportion of respondents with obesity, low social support, and whose income fell below the first quintile reported unmet need due to distance and financial barriers. There was no discernible pattern between the remaining respondent characteristics and types of unmet need.

**Table 3:** Characteristics of respondents reporting an unmet need due to wait, distance or affordability

Variable	Wait (n=1639)		Distance (n=1531)		Could not afford medical care (n=1813)		Could not afford dental care (n=1888)		Could not afford prescribed medicines (n=1899)		Could not afford mental healthcare (n=1268)	
	N	%	N	%	N	%	N	%	N	%	N	%
Sex												
Male	215	45	27	57	44	51	106	52	63	61	19	35
Female	314	55	24	43	50	49	116	48	45	39	44	65
Age												
15–24	35	8	10	24	6	8	15	8	7	8	4	7
25–34	119	23	11	20	16	17	46	21	21	19	22	36
35–44	119	22	10	18	20	20	46	20	12	11	15	23
45–54	119	21	3	5	25	26	55	25	31	28	10	16
55–64	78	13	6	12	14	14	37	15	16	14	11	17
65 and over	59	12	11	21	13	15	23	11	21	20	1	1
Marital status												
No	187	37	24	50	43	46	97	45	51	50	38	62
Yes	342	63	27	50	51	54	125	55	57	50	25	38
Immigrant												
No	322	62	29	56	60	65	134	61	80	73	31	51
Yes	207	38	22	44	34	35	88	39	28	27	32	49
Education												
Primary and pre-primary	34	7	5	9	11	12	18	8	12	12	5	7
Secondary and post-secondary	264	50	27	54	66	71	146	66	76	71	37	61
Tertiary	231	43	19	37	17	17	58	25	20	18	21	32
Job status												
Employed	334	62	25	47	52	54	136	61	53	47	32	50
Unemployed	15	3	1	2	8	9	13	6	5	5	7	12
Retired	91	17	12	22	16	16	32	14	24	21	4	6
Student, domestic, compulsory service	74	15	10	23	11	14	30	14	19	20	13	21
Permanently disabled and other inactive status	15	3	3	6	7	7	11	5	7	7	7	12
Social support												
Low	19	4	4	7	8	8	16	7	10	9	10	16
Moderate	228	44	24	48	39	42	84	39	37	35	35	55
High	282	52	23	45	47	50	122	54	61	56	18	29
Household income												
Below 1st quintile (lowest)	100	19	21	40	35	37	67	30	34	32	25	40
Between 1st and 2nd quintile	82	16	13	30	13	15	50	23	18	17	13	20
Between 2nd and 3rd quintile	133	24	6	11	24	26	57	26	33	30	15	23
Between 3rd and 4th quintile	108	21	7	13	12	13	29	13	12	11	7	12
Between 4th and 5th quintile (highest)	106	20	4	7	10	10	19	8	11	10	3	4
Informal carer												
No	419	79	43	84	75	80	183	82	88	82	53	84
Yes	110	21	8	16	19	20	39	18	20	18	10	16
BMI												
Normal or underweight (<25)	286	53	23	45	47	51	93	42	42	39	28	45
Overweight (25–29)	141	28	14	28	19	19	78	34	33	30	19	30
Obese (≥30)	102	19	14	27	28	30	51	23	33	31	16	25
Smoker												
No	422	80	39	76	60	65	150	67	84	78	38	60
Occasionally	27	5	3	7	10	10	12	5	3	2	2	3
Daily	80	15	9	18	24	24	60	28	21	20	23	37
Alcohol consumption												
Never	102	19	13	25	22	25	55	25	23	22	14	23
Ex-drinkers	61	11	9	18	15	16	36	17	13	12	12	20
Irregularly	51	9	9	15	12	12	23	10	16	15	7	10
Regularly	180	34	10	19	27	27	52	24	34	31	19	30
Everyday	135	26	10	23	18	20	56	25	22	20	11	16
Self-assessed health												
Good/very good	325	61	17	34	40	41	113	50	56	51	29	46
Fair	142	27	20	40	26	29	74	34	31	30	18	28
Bad/very bad	62	12	14	26	28	30	35	16	21	19	16	26
Chronic disease												
No	132	25	10	20	25	26	63	28	26	32	8	13
Yes	397	75	41	80	69	74	159	72	82	68	55	87
Limitations in activities due to health problems												
No limitations	272	51	17	35	36	37	106	48	44	40	24	37
Limited/severely limited	257	49	34	65	58	63	116	52	64	60	39	63
Canton												
Capellen	54	10	3	6	11	12	24	10	14	12	8	12
Clervaux	14	3	5	9	4	5	7	4	8	8	2	3
Diekirch	17	3	5	11	7	8	11	6	5	5	2	3
Echternach	14	3	2	5	4	4	9	4	3	3	2	3
Esch sur Alzette	143	26	12	22	30	32	70	32	29	25	18	29
Grevenmacher	37	7	5	10	6	7	15	7	9	9	4	8
Luxembourg	171	32	13	26	18	18	55	23	19	17	20	31
Mersch	37	7	1	2	5	5	10	5	9	8	1	1
Redange	16	3	1	3	3	3	4	2	2	2	1	1
Remich	17	3	2	4	3	3	9	4	5	5	3	6
Vianden	3	1	1	2	1	1	3	1	2	2	1	2
Wiltz	6	1	1	2	2	3	5	2	3	3	1	1

N refers to number of respondents in estimation sample and % is percentage of estimation sample weighted for age, sex and district of residence.

BMI, body mass index.

Table 4 displays the adjusted ORs from the multivariate logistic regression models. Respondents who reported bad or very bad health were associated with a higher risk of experiencing unmet need for every component, compared with those with good or very good health. Variables positively associated with reporting unmet need due to wait times included being female (OR 1.52, 95% CI 1.19 to 1.93), having a chronic condition (OR 1.45, 95% CI 1.11 to 1.91) and limitations in activities due to health problems (OR 1.33, 95% CI 1.02 to 1.73). Respondents aged 65 years and over were less likely to experience long wait times in comparison to the 15–24 age group (OR 0.44, 95% CI 0.21 to 0.90). A lower risk of experiencing delays due to distance or transportation problems was associated with the age groups 35–44 (OR 0.20, 95% CI 0.05 to 0.75), 45–54 (OR 0.04, 95% CI 0.01 to 0.23) and 55–64 (OR 0.14, 95% CI 0.03 to 0.63) as well as the income groups between the second and third quintiles (OR 0.19, 95% CI 0.06 to 0.54) and the fourth and fifth quintiles (OR 0.14, 95% CI 0.04 to 0.57). There is some consistency in determinants of unmet need arising from financial barriers for different types of care. Moderate or high social support was associated with a lower risk for dental care, prescribed medicines and mental healthcare. Married respondents were also associated with a lower risk for prescribed medicines (OR 0.53, 95% CI 0.33 to 0.84) and mental healthcare (OR 0.40, 95% CI 0.21 to 0.79). Non-participation in the labour force due to studies, domestic work or compulsory service was associated with a higher risk of being unable to afford prescribed medicines (OR 2.22, 95% CI 1.04 to 4.76) and mental healthcare (OR 2.95, 95% CI 1.28 to 6.77). Income between the first and second quintiles was associated with a lower risk of unmet need for medical care (OR 0.37, 95% CI 0.18 to 0.78) and prescribed medicines (OR 0.49, 95% CI 0.26 to 0.95); income between the third and fourth quintiles was associated with a lower risk for dental care (OR 0.46, 95% CI 0.26 to 0.79) and prescribed medicines (OR 0.33, 95% CI 0.15 to 0.70) and income between the fourth and fifth quintiles was associated with a lower risk for dental care (OR 0.29, 95% CI 0.16 to 0.53), prescribed medicines (OR 0.38, 95% CI 0.17 to 0.82) and mental healthcare (OR 0.17, 95% CI 0.05 to 0.61). Daily smokers were associated with a higher risk of unmet need for dental (OR 1.82, 95% CI 1.28 to 2.60) and mental healthcare (OR 2.15, 95% CI 1.10 to 4.17) compared with non-smokers. Immigrants were at higher risk of being unable to afford prescribed medicines (OR 0.58, 95% CI 0.35 to 0.97).

**Table 4:** Multivariate logistic regression models for unmet need due to wait, distance or affordability

Variable	Wait (n=1639)	Distance (n=1531)	Could not afford medical care (n=1813)	Could not afford dental care (n=1888)	Could not afford prescribed medicines (n=1899)	Could not afford mental healthcare (n=1268)
Sex						
Male	1	1	1	1	1	1
Female	1.52 (1.19 to 1.93)**	0.83 (0.40 to 1.75)	1.04 (0.64 to 1.71)	0.96 (0.69 to 1.33)	0.55 (0.35 to 0.88)*	2.89 (1.41 to 5.94)**
Age						
15–24	1	1	1	1	1	1
25–34	1.32 (0.72 to 2.40)	0.27 (0.07 to 1.02)	1.76 (0.49 to 6.27)	1.62 (0.71 to 3.71)	3.22 (0.98 to 10.57)	6.39 (1.75 to 23.35)**
35–44	1.04 (0.55 to 1.93)	0.20 (0.05 to 0.75)*	1.87 (0.51 to 6.81)	1.36 (0.58 to 3.16)	1.53 (0.40 to 5.86)	2.49 (0.62 to 9.97)
45–54	1.00 (0.54 to 1.83)	0.04 (0.01 to 0.23)***	2.09 (0.58 to 7.49)	1.53 (0.66 to 3.56)	3.65 (1.05 to 12.66)*	1.33 (0.31 to 5.60)
55–64	0.59 (0.31 to 1.12)	0.14 (0.03 to 0.63)*	1.55 (0.39 to 6.06)	1.51 (0.62 to 3.68)	2.24 (0.58 to 8.58)	2.18 (0.51 to 9.35)
65 and over	0.44 (0.21 to 0.90)*	0.27 (0.07 to 1.07)	2.28 (0.50 to 10.36)	1.21 (0.46 to 3.19)	2.98 (0.76 to 11.75)	0.17 (0.01 to 2.16)
Marital status						
No	1	1	1	1	1	1
Yes	1.16 (0.89 to 1.52)	1.14 (0.47 to 2.78)	0.73 (0.43 to 1.23)	0.76 (0.53 to 1.08)	0.53 (0.33 to 0.84)**	0.40 (0.21 to 0.79)**
Immigrant						
No	1	1	1	1	1	1
Yes	0.79 (0.61 to 1.01)	1.18 (0.55 to 2.52)	0.79 (0.49 to 1.26)	0.85 (0.61 to 1.19)	0.58 (0.35 to 0.97)*	1.37 (0.68 to 2.76)
Education						
Primary and pre-primary	1	1	1	1	1	1
Secondary and post-secondary	1.14 (0.71 to 1.85)	1.26 (0.42 to 3.79)	1.48 (0.61 to 3.55)	1.60 (0.85 to 3.02)	1.35 (0.64 to 2.85)	1.55 (0.45 to 5.35)
Tertiary	1.40 (0.82 to 2.37)	2.59 (0.80 to 8.41)	0.55 (0.20 to 1.53)	1.20 (0.59 to 2.44)	0.67 (0.27 to 1.65)	1.22 (0.29 to 5.11)
Job status						
Employed	1	1	1	1	1	1
Unemployed	0.62 (0.31 to 1.25)	0.21 (0.04 to 1.11)	1.74 (0.59 to 5.11)	1.4 (0.65 to 3.02)	1.51 (0.48 to 4.75)	1.51 (0.45 to 5.09)
Retired	1.22 (0.76 to 1.95)	0.91 (0.29 to 2.83)	0.60 (0.23 to 1.54)	0.58 (0.32 to 1.05)	0.88 (0.38 to 2.02)	1.20 (0.30 to 4.71)
Student, domestic, compulsory service	1.25 (0.82 to 1.91)	0.66 (0.23 to 1.85)	0.81 (0.33 to 2.01)	1.01 (0.58 to 1.76)	2.22 (1.04 to 4.76)*	2.95 (1.28 to 6.77)*
Permanently disabled and other inactive status	0.80 (0.38 to 1.69)	1.60 (0.29 to 8.81)	0.92 (0.35 to 2.41)	0.90 (0.44 to 1.84)	1.40 (0.58 to 3.37)	2.97 (0.92 to 9.64)
Social support						
Low	1	1	1	1	1	1
Moderate	1.54 (0.81 to 2.92)	0.84 (0.26 to 2.72)	0.60 (0.22 to 1.66)	0.46 (0.23 to 0.95)*	0.32 (0.14 to 0.77)*	0.27 (0.09 to 0.76)*
High	0.95 (0.50 to 1.81)	0.64 (0.19 to 2.22)	0.44 (0.16 to 1.23)	0.42 (0.21 to 0.86)*	0.30 (0.13 to 0.70)**	0.11 (0.03 to 0.35)***
Household income						
Below 1st quintile (lowest)	1	1	1	1	1	1
Between 1st and 2nd quintile	0.85 (0.57 to 1.26)	0.74 (0.31 to 1.76)	0.37 (0.18 to 0.78)**	0.77 (0.48 to 1.21)	0.49 (0.26 to 0.95)*	0.49 (0.22 to 1.12)
Between 2nd and 3rd quintile	1.01 (0.70 to 1.48)	0.19 (0.06 to 0.54)**	0.73 (0.40 to 1.36)	0.77 (0.49 to 1.21)	0.77 (0.43 to 1.36)	0.78 (0.35 to 1.72)
Between 3rd and 4th quintile	1.16 (0.77 to 1.73)	0.35 (0.11 to 1.10)	0.47 (0.22 to 1.01)	0.46 (0.26 to 0.79)**	0.33 (0.15 to 0.70)**	0.47 (0.17 to 1.29)
Between 4th and 5th quintile (highest)	0.91 (0.59 to 1.38)	0.14 (0.04 to 0.57)**	0.46 (0.20 to 1.07)	0.29 (0.16 to 0.53)***	0.38 (0.17 to 0.82)*	0.17 (0.05 to 0.61)**
Informal carer						
No	1	1	1	1	1	1
Yes	1.36 (1.02 to 1.82)*	0.92 (0.42 to 2.03)	1.17 (0.67 to 2.03)	0.96 (0.63 to 1.45)	0.91 (0.53 to 1.56)	0.72 (0.32 to 1.64)
BMI						
Underweight or normal	1	1	1	1	1	1
Overweight	0.81 (0.62 to 1.06)	1.04 (0.49 to 2.22)	0.49 (0.27 to 0.90)*	1.28 (0.90 to 1.83)	1.17 (0.69 to 1.99)	1.92 (0.94 to 3.91)
Obese	0.99 (0.72 to 1.35)	1.24 (0.61 to 2.55)	1.09 (0.62 to 1.92)	1.35 (0.89 to 2.02)	1.72 (0.96 to 3.08)	1.49 (0.68 to 3.27)
Smoker (no)						
No	1	1	1	1	1	1
Occasionally	1.06 (0.64 to 1.74)	1.38 (0.43 to 4.43)	2.76 (1.27 to 5.99)*	0.96 (0.49 to 1.86)	0.39 (0.10 to 1.44)	0.35 (0.07 to 1.64)
Daily	0.91 (0.66 to 1.26)	0.87 (0.38 to 1.96)	1.25 (0.72 to 2.15)	1.82 (1.28 to 2.60)**	0.90 (0.54 to 1.50)	2.15 (1.10 to 4.17)*
Alcohol consumption						
Never	1	1	1	1	1	1
Ex-drinkers to drinkers	1.49 (0.95 to 2.35)	1.09 (0.41 to 2.90)	1.33 (0.59 to 3.01)	1.60 (0.93 to 2.77)	1.10 (0.49 to 2.49)	1.92 (0.73 to 5.08)
Irregularly	1.06 (0.68 to 1.63)	1.68 (0.64 to 4.38)	1.22 (0.54 to 2.73)	0.89 (0.51 to 1.55)	1.19 (0.59 to 2.41)	1.27 (0.40 to 4.04)
Regularly	1.12 (0.81 to 1.55)	0.75 (0.29 to 1.94)	0.89 (0.48 to 1.65)	0.63 (0.41 to 0.97)*	0.97 (0.54 to 1.74)	1.36 (0.59 to 3.17)
Everyday	1.07 (0.75 to 1.53)	0.97 (0.37 to 2.53)	0.85 (0.41 to 1.74)	1.02 (0.65 to 1.59)	0.80 (0.41 to 1.56)	1.20 (0.49 to 2.98)
Self to assessed health						
Good/very good	1	1	1	1	1	1
Fair	1.21 (0.90 to 1.62)	2.45 (1.17 to 5.12)*	1.26 (0.66 to 2.43)	1.63 (1.10 to 2.40) *	0.9 (0.52 to 1.55)	1.01 (0.44 to 2.28)
Bad/very bad	2.41 (1.53 to 3.80)***	7.12 (2.91 to 17.44)***	5.35 (2.39 to 11.95)***	3.26 (1.86 to 5.71)***	2.22 (1.04 to 4.71)*	3.58 (1.25 to 10.30)*
Chronic disease						
No	1	1	1	1	1	1
Yes	1.45 (1.11 to 1.91)**	1.42 (0.60 to 3.37)	0.69 (0.38 to 1.28)	0.83 (0.57 to 1.21)	0.84 (0.48 to 1.44)	2.33 (0.89 to 6.08)
Limitations in activities due to health problems						
No limitations	1	1	1	1	1	1
Limited/severely limited	1.33 (1.02 to 1.73)*	1.37 (0.67 to 2.81)	1.39 (0.74 to 2.64)	1.15 (0.79 to 1.67)	1.49 (0.86 to 2.58)	1.53 (0.64 to 3.66)
Canton						
Esch zur Alzette	1	1	1	1	1	1
Capellen	1.21 (0.80 to 1.82)	1.08 (0.25 to 4.57)	1.42 (0.64 to 3.13)	1.41 (0.83 to 2.40)	2.12 (1.04 to 4.30)*	1.63 (0.61 to 4.37)
Clervaux	0.90 (0.46 to 1.75)	3.31 (0.78 to 14.14)	1.08 (0.31 to 3.81)	0.76 (0.31 to 1.88)	2.06 (0.77 to 5.53)	0.53 (0.12 to 2.31)
Diekirch	0.92 (0.49 to 1.72)	3.54 (1.02 to 12.32)	2.27 (0.87 to 5.95)	1.31 (0.60 to 2.83)	1.88 (0.68 to 5.18)	0.67 (0.12 to 3.85)
Echternach	1.43 (0.69 to 2.97)	2.02 (0.36 to 11.36)	1.63 (0.40 to 6.67)	1.47 (0.68 to 3.17)	1.13 (0.29 to 4.37)	1.19 (0.14 to 10.34)
Grevenmacher	1.67 (1.01 to 2.78)	3.06 (1.02 to 9.15)	1.38 (0.49 to 3.90)	1.27 (0.65 to 2.49)	2.53 (1.08 to 5.96)*	1.28 (0.30 to 5.51)
Luxembourg	1.27 (0.94 to 1.72)	1.40 (0.59 to 3.30)	0.80 (0.42 to 1.50)	0.99 (0.65 to 1.50)	1.02 (0.54 to 1.94)	1.54 (0.73 to 3.25)
Mersch	1.67 (1.01 to 2.76)*	0.43 (0.05 to 3.74)	1.11 (0.39 to 3.22)	0.88 (0.40 to 1.94)	2.38 (1.00 to 5.67)	0.29 (0.04 to 2.23)
Redange	1.07 (0.56 to 2.04)	0.95 (0.09 to 10.02)	0.91 (0.27 to 3.04)	0.53 (0.17 to 1.60)	0.60 (0.13 to 2.80)	0.80 (0.08 to 7.68)
Remich	0.99 (0.52 to 1.90)	1.64 (0.32 to 8.40)	0.75 (0.23 to 2.40)	1.05 (0.50 to 2.20)	1.51 (0.58 to 3.97)	3.45 (0.94 to 12.74)
Vianden	0.59 (0.14 to 2.46)	6.92 (0.62 to 77.07)	0.80 (0.14 to 4.45)	1.04 (0.29 to 3.67)	2.13 (0.58 to 7.77)	1.64 (0.24 to 11.19)
Wiltz	0.39 (0.15 to 0.99)	0.45 (0.07 to 3.16)	1.79 (0.33 to 9.75)	0.95 (0.34 to 2.71)	2.35 (0.57 to 9.65)	0.40 (0.05 to 3.14)
Constant	0.15 (0.05 to 0.42)***	0.08 (0.01 to 0.67)*	0.10 (0.02 to 0.57)*	0.22 (0.06 to 0.78)*	0.14 (0.03 to 0.59)**	0.01 (0.00 to 0.11)***

Estimatesare weighted for age, sex and district of residence. Data are OR adjusted for explanatory variables (95%CI).

*P<0.05, **p<0.01, ***p<0.001.

BMI, body mass index.

Online supplemental appendix 1, table A3 shows the results of the sensitivity analysis that included a binary variable measuring income non-response in each model. This variable was statistically significant in only the model for affordability of dental care with non-reporting of income associated with a lower risk of reporting unmet need due to the affordability of dental care (OR 0.64, 95% CI 0.42 to 0.97). Results for all models were largely unchanged. As in the main analyses, a lower risk of unmet need due to the affordability of dental care was associated with moderate (OR 0.43, 95% CI 0.24 to 0.78) or high (OR 0.40, 95% CI 0.22 to 0.72) social support, income between the third and fourth quintile (OR 0.48, 95% CI 0.28 to 0.81) and fourth and fifth quintile (OR 0.30, 95% CI 0.17 to 0.54) and regular alcohol consumption (OR 0.60, 95% CI 0.42 to 0.87).

## Discussion

### A statement of the principal findings

In this paper, we investigated the prevalence and determinants of unmet need in Luxembourg using EHIS 2 data. The most common barrier to accessing healthcare arose from long waits (32%). Compared with other EU countries who collected this data using the EHIS, Luxembourg had the highest proportion of respondents who reported unmet need due to long waits.^52^ Respondents were most likely to report being unable to afford dental care (12%), followed by prescribed medicines (6%)—a result that is on par with the EU averages (12.3% and 4.6%, respectively).^53^ The percentage of respondents who reported being unable to afford mental healthcare (5%) was higher than the EU average (2.7%).^53^ The association of various determinants of unmet need varied according to the different components. However, bad or very bad self-reported health was positively associated with every component of unmet need. This group was twice as likely to report unmet need due to wait time, seven times more likely to report unmet need due to distance or transportation, and two to five times more likely to report unmet need due to affordability of services compared with respondents who assessed their health as good or very good. Another notable finding was the clear income gradient for unmet need due to the affordability of care, with the highest income quintile 71% less likely to report unmet need for dental care, 62% less likely for prescribed medications and 83% less likely for mental healthcare compared with the lowest income quintile.

### Comparisons with previous studies

Previous studies^14 16 21 23 25^ have investigated unmet need within countries using EU-SILC data. The use of the EHIS brought two important advantages that moved us beyond these studies. First, we undertook separate analyses of unmet need arising from long waits, distance or transportation problems, and affordability of medical, dental and mental healthcare and prescribed medicines. Second, we exploited a richer set of health variables, which enabled us to investigate risk factors including BMI, smoking and alcohol consumption as determinants of unmet need. Moreover, this is the first study that used the EHIS to investigate the determinants of unmet need in the general population. Previous studies used the EHIS to investigate unmet need for specific population groups. Sakellariou *et al*^30^ investigated whether people with disabilities had higher unmet need for healthcare due to waiting times, distance or transport problems and unaffordability of medical and mental healthcare and prescribed medicines compared with people without disabilities in the UK. Rotarou *et al*^29^ conducted a similar study for Greece. Hoebel *et al*^28^ focused only on respondents aged 50–85 years of age in Germany to investigate unmet need due to the affordability of medical, dental and mental healthcare and prescribed medicines. They did not consider long waits or distance or transportation problems.

Three previous studies^14 19 23^ used EU-SILC data on reasons for unmet need to investigate associations with various determinants. Two studies^14 23^ considered the main reason for unmet need due to availability (waiting lists) and accessibility (affordability and distance/transportation) while the remaining study^19^ considered affordability and distance/transportation as separate categories. Comparable to our results, these studies reported a positive association between bad and very bad health (compared with very good) and unmet need due to availability^14^ and accessibility^14 23^ or only affordability of care.^19^ One study^19^ found that social support as measured by the ability to seek help was associated with a lower probability of unmet needs for medical care arising from economic costs, reflecting our finding that higher levels of social support were associated with lower unmet need due to affordability of care. Our result that females were associated with higher unmet need due to wait times was not supported by these studies, which found evidence of higher unmet need for females due to accessibility^14^ and affordability of care.^19^ Women and respondents with bad or very bad self-assessed health may have been unable to afford mental healthcare because they have higher need. A previous study^49^ investigated the burden of depression in Luxembourg and reported a higher prevalence rate of depression symptoms in females compared with males and in those who perceived their health as poor, compared with those who perceived their health as good. This study also found that good social support was associated with a lower risk of depression, which could indicate a lower need for mental healthcare and hence lower unmet need arising from the affordability of mental healthcare.

Similar to previous studies,^14 23^ we found that higher income was associated with a lower risk of unmet need due to distance and affordability of services. Income disparities in unmet need due to the affordability of services may have reflected the requirement for patients to pay the full costs of many services directly to providers at the point of use. Our finding of no statistically significant relationship between income and unmet need due to waits was also reflected in previous studies.^14 16 19 23^ Although we did not find any association between unmet need due to waits and socioeconomic variables including education, income and job status, previous studies reported evidence of inequalities in waiting times related to education and income.^54–56^ The EHIS data allowed us to consider the association between health behaviours and unmet need. We found that these variables were only associated with unmet need arising from the affordability of healthcare. Therefore, our results add to a previous study from Canada^50^ that considered the relationship between obesity, drinking and smoking and unmet need due to health system factors (including unavailability of services and long waiting times and excluding cost) and found evidence of a positive association between smoking (both daily and occasional) and unmet need.

### Study limitations

Our study is subject to limitations arising from our data, some of which could in principle be overcome in future research, via development and adjustment of the EHIS questionnaire. As unmet need was self-reported, the data may suffer from the limitations inherent in survey data, including recall or response bias (respondent inaccurately remembers or misunderstands the question).^57 58^ Moreover, self-reported unmet need may be influenced by unobservable factors such as cultural norms, health literacy and expectations of health services.^15 20 59 60^ The EHIS did not ask respondents if they perceived long waits across the healthcare system or for particular services (primary care, cancer care, elective treatment, diagnostic tests), nor the length of time perceived as long. The survey sampling did not include sections of society who lack any insurance coverage and may have high unmet need including the homeless and irregular immigrants and their families.^34^ Individuals living in collective households (eg, a boarding house or hostel or a dormitory in an educational establishment)^43^ and institutions were also excluded. Despite these limitations, survey data remains the best available source.^59^ We excluded observations with missing data for household income. The profile of respondents who did not report household income indicated that they were young people in full-time education who may have been living in the family home. Perhaps these respondents did not know the value of the household income, because they were not a main earner. We could assume that some of these respondents were less likely to bear the financial costs of care and therefore less likely to report unmet need due to the affordability of care. Indeed, the results of the sensitivity analysis as shown in online supplemental appendix 1, table A3 suggested this was the case, although the variable measuring income non-response is only statistically significant in the model measuring affordability of dental care.

### Implications for clinicians and policy-makers

The Luxembourgish health system is characterised by a high rate of population health insurance coverage, a comprehensive benefit package and a relatively low level of out-of-pocket payments. Nevertheless, access to healthcare is an important issue, particularly in relation to wait times, as reflected in our finding that the majority of respondents reported unmet need due to long waiting times to obtain an appointment. While Luxembourg has introduced reforms to address waiting times in emergency departments and for cancer care, there is potential to implement measures that could address waiting times for other priority services where patients must wait to obtain an appointment, for example, primary care. Unlike Luxembourg, in many European countries, nurses or physician assistants play a key role in primary care by providing health education, immunisations and routine checks of people with chronic illnesses,^8^ allowing general practitioners more time to attend to patients with more complex needs. Policy-makers could also consider the introduction of a maximum waiting time for specialist consultations, a policy adopted by several European countries.

In response to the COVID-19 pandemic, teleconsultations are now reimbursed by the CNS. The retention and embedding of telemedicine into the healthcare system in Luxembourg could help to reduce unmet need due to distance and transportation, particularly for those with poor health who may face difficulties in travelling to healthcare providers. The increased use of teleconsultations could also help to address waiting times for particular services, for example, primary and specialist care.

While the introduction of a universal third-party payment system may help to address unmet need due to the affordability of care this reform may not be sufficient by itself as individuals may still face a financial barrier to care due to copayments. For example, while prescribed medicines are currently covered by the third-party payment system, meaning that individuals only pay the copayment, our results show that 6% of respondents reported unmet need to affordability of prescribed medicines. Therefore, additional reforms aimed at reducing the risk of unmet need could address copayments and target high-risk groups such as those with low incomes or in poor health, similar to policies in Belgium and France (as described in the Introduction section). Currently the CNS does not reimburse visits to psychologists and expanding health insurance coverage to psychologists could help to address unmet need due to the affordability of mental healthcare, especially for at-risk groups including individuals who are female, aged 25–34, students or undertaking domestic duties or compulsory service and with low income.

### Implications for future research

Future revisions of the EHIS could expand the social variables collected, for example, by adding information on ethnicity or parents’ country of birth. Additional questions on social support could help to distinguish between emotional, instrumental and informational support.^19^ Future research could investigate the issue of waiting times in Luxembourg for different types of services in order to inform policy measures to reduce unmet need. Recent policies focused on the reorganisation of cancer care delivery and introduction of waiting time targets for cancer care could be evaluated. Given our finding of income disparities in unmet need due to the affordability of care, an evaluation of the implementation of the third-party payment system to date would inform its planned expansion and potential further adjustments. The collection of a third wave of EHIS data will enable an assessment of trends in unmet need in Luxembourg over time. This research also demonstrates that the EHIS is a useful resource that could be used for future within-country and between-country analyses of unmet need for healthcare in the general population in European countries. While previous cross-country studies have examined patient and health system characteristics associated with unmet need across European countries for medical and dental care, the EHIS could complement these studies by extending the analyses to unmet need arising from the affordability of mental healthcare and prescribed medicines.

## Conclusions

Compared with other EU countries, Luxembourg has high per capita public healthcare expenditures, a low share of total health spending financed by out-of-pocket payments and a comprehensive benefit package.^61 62^ Nevertheless, this study provides evidence that some individuals experienced difficulties accessing care, particularly due to long waits and affordability of care. Future reforms to improve access to healthcare should first target high-risk groups including those with low incomes and poor health. Future policies to address unmet need should also consider other vulnerable populations who lack formal healthcare coverage including the homeless, undocumented immigrants and those in the informal labour market.

## Supplementary Material

Reviewer comments

Author's manuscript

## Data Availability

Data may be obtained from a third party and are not publicly available. Information on the data access procedure for the 2014 EHIS for Luxembourg is available on the Ministry of Health website: https://sante.public.lu/fr/statistiques/ehis/ehis-methodologie/ehis-formulaire-demande-de-donnees.docx.

## References

[1] WHO. Health systems: universal health coverage, 2020. Available: https://www.who.int/healthsystems/universal_health_coverage/en/#:~:text=Universal%20health%20coverage%20is%20defined,the%20user%20the%20financial%20hardship

[2] European Commission. European Pillar of social rights, 2021. Available: https://ec.europa.eu/info/strategy/priorities-2019-2024/economy-works-people/jobs-growth-and-investment/european-pillar-social-rights_en#background

[3] European Commission. The European Pillar of social rights in 20 principles, 2021. Available: https://ec.europa.eu/info/strategy/priorities-2019-2024/economy-works-people/jobs-growth-and-investment/european-pillar-social-rights/european-pillar-social-rights-20-principles_en

[4] Gulliford M, Figueroa-Munoz J, Morgan M, et al. What does 'access to health care' mean? J Health Serv Res Policy 2002;7:186–8. 10.1258/135581902760082517 12171751

[5] European Union. Opinion on benchmarking access to healthcare in the EU. Brussels: European Union, 2017.

[6] Eurostat. Methodological guidelines and description of EU-SILC target variables PH010: General health, 2017. Available: https://ec.europa.eu/eurostat/documents/1012329/8655367/PERSONAL+DATA_Health.pdf/0a942278-dd03-47f6-9af6-3f3000f678ce

[7] Allin S, Masseria C. Unmet need as an indicator of health care access. Eurohealth 2009;15:7–10 http://eprints.lse.ac.uk/id/eprint/28454

[8] OECD. Waiting times for health services next in line, in OECD health policy studies. Paris: OECD Publishing, 2020.

[9] Bouckaert N, Maertens de Noordhout C, Van de Voorde C. Health system performance assessment: how equitable is the Belgian system? Brussels: Belgian Health Care Knowledge Centre (KCE), 2020.

[10] Goujard A. France: improving the efficiency of the healthcare system, in OECD Economics Department Working Papers No. 1455. Paris: OECD Publishing, 2018.

[11] European Observatory on Health Systems and Policies. State of health in the EU. Brussels: OECD Publishing, 2019.

[12] European Union. Expert Panel on effective ways of investing in Health (EXPH), Report on Access to Health Services in the European Union. Brussels: European Commission, 2016.

[13] OECD. Health for Everyone?: Social Inequalities in Health and Health Systems, in OECD Health Policy Studies. Paris: OECD Publishing, 2019.

[14] Cavalieri M. Geographical variation of unmet medical needs in Italy: a multivariate logistic regression analysis. Int J Health Geogr 2013;12:27. 10.1186/1476-072X-12-27 23663530 PMC3662566

[15] Chaupain-Guillot S, Guillot O. Health system characteristics and unmet care needs in Europe: an analysis based on EU-SILC data. Eur J Health Econ 2015;16:781–96. 10.1007/s10198-014-0629-x 25186072

[16] Connolly S, Wren M-A. Unmet healthcare needs in Ireland: analysis using the EU-SILC survey. Health Policy 2017;121:434–41. 10.1016/j.healthpol.2017.02.009 28233599

[17] Detollenaere J, Hanssens L, Vyncke V, et al. Do we Reap what we sow? exploring the association between the strength of European primary healthcare systems and inequity in unmet need. PLoS One 2017;12:e0169274. 10.1371/journal.pone.0169274 28046051 PMC5207486

[18] Elstad JI. Income inequality and foregone medical care in Europe during the great recession: multilevel analyses of EU-SILC surveys 2008-2013. Int J Equity Health 2016;15:101. 10.1186/s12939-016-0389-6 27388561 PMC4936318

[19] Fiorillo D. Reasons for unmet needs for health care: the role of social capital and social support in some Western EU countries. Int J Health Econ Manag 2020;20:79-98. 10.1007/s10754-019-09271-0 31297726

[20] Israel S. How social policies can improve financial accessibility of healthcare: a multi-level analysis of unmet medical need in European countries. Int J Equity Health 2016;15:41. 10.1186/s12939-016-0335-7 26944542 PMC4779225

[21] Legido-Quigley H, Karanikolos M, Hernandez-Plaza S, et al. Effects of the financial crisis and Troika austerity measures on health and health care access in Portugal. Health Policy 2016;120:833–9. 10.1016/j.healthpol.2016.04.009 27263063

[22] Madureira-Lima J, Reeves A, Clair A, et al. The great recession and inequalities in access to health care: a study of unemployment and unmet medical need in Europe in the economic crisis. Int J Epidemiol 2018;47:58–68. 10.1093/ije/dyx193 29024999 PMC5837221

[23] Popovic N, Terzic-Supic Z, Simic S, et al. Predictors of unmet health care needs in Serbia; analysis based on EU-SILC data. PLoS One 2017;12:e0187866. 10.1371/journal.pone.0187866 29117216 PMC5678705

[24] Reeves A, McKee M, Mackenbach J, et al. Public pensions and unmet medical need among older people: cross-national analysis of 16 European countries, 2004-2010. J Epidemiol Community Health 2017;71:174–80. 10.1136/jech-2015-206257 27965315 PMC5284463

[25] Yardim MS, Uner S. Equity in access to care in the era of health system reforms in turkey. Health Policy 2018;122:645–51. 10.1016/j.healthpol.2018.03.016 29598885

[26] Fjær EL, Stornes P, Borisova LV, et al. Subjective perceptions of unmet need for health care in Europe among social groups: findings from the European social survey (2014) special module on the social determinants of health. Eur J Public Health 2017;27:82–9. 10.1093/eurpub/ckw219 28355635

[27] Quintal C, Lourenço Óscar, Ramos LM, et al. No unmet needs without needs! assessing the role of social capital using data from European social survey 2014. Health Policy 2019;123:747–55. 10.1016/j.healthpol.2019.06.001 31213332

[28] Hoebel J, Rommel A, Schröder SL, et al. Socioeconomic inequalities in health and perceived unmet needs for healthcare among the elderly in Germany. Int J Environ Res Public Health 2017;14. 10.3390/ijerph14101127. [Epub ahead of print: 26 09 2017]. PMC5664628 28954436

[29] Rotarou ES, Sakellariou D. Access to health care in an age of austerity: disabled people’s unmet needs in Greece. Crit Public Health 2019;29:48–60. 10.1080/09581596.2017.1394575

[30] Sakellariou D, Rotarou ES. Access to healthcare for men and women with disabilities in the UK: secondary analysis of cross-sectional data. BMJ Open 2017;7:e016614. 10.1136/bmjopen-2017-016614 PMC5629679 28893735

[31] d'Gesondheetskees CNS. Rapport annuel 2018. Luxembourg: CNS d'Gesondheetskees, 2019.

[32] Statec. Le Portail des Statistiques Grand-Duché de Luxembourg, 2020. Available: https://statistiques.public.lu/stat/TableViewer/tableView.aspx?ReportId=12853&IF_Language=fra&MainTheme=2&FldrName=1

[33] Berthet F et al. Health Systems in Transition Luxembourg. Brussels: European Observatory on Health Systems and Policies, 2015.

[34] Monde Mdu, tous La santé pour. Rapport annuel 2018 Médecins Du Monde Luxembourg Médecins du Monde Luxembourg; 2019.

[35] CNS d'Gesondheetskees. Reimbursement, 2019. Available: https://cns.public.lu/en/assure/remboursement-prise-charge.html

[36] OECD. Health systems characteristics survey. Available: https://qdd.oecd.org/subject.aspx?Subject=hsc

[37] Institut national d'assurance maladie-invalidité. Tiers payant : ne payez que votre part, la mutualité paie directement le dispensateur de soins, 2017

[38] CNS d'Gesondheetskees. Third-Party social payment, 2020. Available: https://cns.public.lu/en/professionnels-sante/medecins/tiers-payant-social.html

[39] Government of Luxembourg. Réunion du comité quadripartite en matière d'assurance maladie-maternité Communiqué, 2019. Available: https://gouvernement.lu/fr/actualites/toutes_actualites/communiques/2019/11-novembre/13-reunion-quadripartite.html

[40] Luxembourg Ministry of Health, Luxembourg Ministry of Social Security. Étude du fonctionnement des services d’urgence. Luxembourg: Luxembourg Ministry of Health and Luxembourg Ministry of Social Security, 2017.

[41] Luxembourg Ministry of Health and Luxembourg Ministry of Social Security. Communiqué - Conférence de presse (15.12.2017) Vers une amélioration des services d’urgence, 2017. Available: https://sante.public.lu/fr/actualites/2017/12/urgences/urgences.pdf

[42] CNS d'Gesondheetskees. Treatment abroad, 2016. Available: https://cns.public.lu/en/assure/vie-privee/a-etranger.html

[43] Eurostat and European Commission. European Health Interview Survey (EHIS wave 2) Methodological manual. Luxembourg: Publications Office of the European Union, 2013.

[44] Robert Koch-Institut. European health interview survey (EHIS) 2 – background and study methodology. J Health Monitoring 2019;4:66–79. 10.25646/6228 PMC8734125 35146260

[45] Santé.lu. European health interview survey (EHIS), 2018. Available: https://sante.public.lu/fr/statistiques/ehis/index.html

[46] Luxembourg Ministry of Health Directorate of Health, Luxembourg Institute of Health. European health interview survey (EHIS) study methodology. Luxembourg: Luxembourg Ministry of Health Directorate of Health and Luxembourg Institute of Health, 2017.

[47] Bejko D, Ruiz-Castell M, Schritz A, et al. "To survey or to register" is that the question for estimating population incidence of injuries? Arch Public Health 2018;76:76. 10.1186/s13690-018-0322-0 30564315 PMC6296149

[48] Eurostat. Quality report of the second wave of the European Health Interview survey. European Union: Luxembourg: Publications Office of the European Union, 2018.

[49] Ruiz-Castell M, Kandala N-B, Perquin M, et al. Depression burden in Luxembourg: individual risk factors, geographic variations and the role of migration, 2013-2015 European health examination survey. J Affect Disord 2017;222: :41–8. 10.1016/j.jad.2017.06.056 28668715

[50] Bataineh H, Devlin RA, Barham V. Unmet health care and health care utilization. Health Econ 2019;28): :529–42. 10.1002/hec.3862 30693596

[51] StataCorp. Stata Statistical Software: Release 15. College Station, TX: StataCorp LLC, 2017.

[52] Eurostat. Self-Reported unmet needs for health care by sex, age, specific reasons and educational attainment level, 2020. Available: https://appsso.eurostat.ec.europa.eu/nui/show.do?dataset=hlth_ehis_un1e&lang=en

[53] Eurostat. Self-Reported unmet needs for specific health care-related services due to financial reasons by sex, age and educational attainment level, 2020. Available: https://appsso.eurostat.ec.europa.eu/nui/show.do?dataset=hlth_ehis_un2e&lang=en

[54] Simonsen NF, Oxholm AS, Kristensen SR, et al. What explains differences in waiting times for health care across socioeconomic status? Health Econ 2020;29:1764–85. 10.1002/hec.4163 32996212

[55] Siciliani L, Verzulli R. Waiting times and socioeconomic status among elderly Europeans: evidence from share. Health Econ 2009;18:1295–306. 10.1002/hec.1429 19191260

[56] Laudicella M, Siciliani L, Cookson R. Waiting times and socioeconomic status: evidence from England. Soc Sci Med 2012;74:p. 1331–41. 10.1016/j.socscimed.2011.12.049 22425289

[57] Arora VS, Karanikolos M, Clair A, et al. Data resource profile: the European Union statistics on income and living conditions (EU-SILC). Int J Epidemiol 2015;44:451–61. 10.1093/ije/dyv069 25948659

[58] Iezzoni I. *Risk adjustment for performance measurement*. In: Performance measurement for health system improvement. Cambridge: Cambridge University Press, 2009.

[59] Baeten R et al. Inequalities in access to healthcare. A study of national policies. Brussels: European Commission, 2018.

[60] Eurostat. Methodological issues in the analysis of the socioeconomic determinants of health using EU-SILC data, in Eurostat Methodologies and Working Papers. Luxembourg: Publications Office of the European Union, 2010.

[61] OECD. OECD Health Statistics 2020. Paris: OECD Publishing, 2020.

[62] OECD and European Observatory on Health Systems and Policies. Luxembourg: country health profile 2019, state of health in the EU. Paris/European Observatory on Health Systems and Policies, Brussels: OECD Publishing, 2019.

