# Supplementary material for: Investigating unmet need for healthcare using the European Health Interview Survey: a cross-sectional survey study of Luxembourg
Source: BMJ Open. 2021 Aug 3;11(8):e048860. doi: 10.1136/bmjopen-2021-048860 (PMC8336210; doi:10.1136/bmjopen-2021-048860)
Supplement: Supplementary data [file bmjopen-2021-048860supp001.pdf]

Appendix 1. Unmet Needs for Health Care Questions

3.22. In the **past 12 months**, have you experienced delay in getting health care because/due to…?

	Yes	No	No need for healthcare
The time needed to obtain an appointment was too long	☐	☐	☐
Distance or transportation problems	☐	☐	☐

3.23. Was there any time in the **past 12 months** you needed the following kinds of care, but could not afford it?

	Yes	No	No need for healthcare
Medical care	☐	☐	☐
Dental care	☐	☐	☐
Prescribed medicines	☐	☐	☐
Mental health care (by a psychologist or a psychiatrist for example)	☐	☐	☐

Source: Santé.lu. European Health Interview Survey (EHIS) 2018. Available from: https://sante.public.lu/fr/statistiques/ehis/ehis-methodologie/ehis-questionnaire-en.pdf

Appendix 2.

Table A1. Missing data for independent variables

Variable	N	%
Sex	0	0
Age	0	0
Marital status	123	3.34
Immigrant	27	0.71
Education	182	4.82
Employment status	284	7.30
Household income	1,116	28.84
Social support	66	1.69
Informal carer	170	4.46
BMI	58	1.43
Smoker	44	1.11
Alcohol consumption	143	3.64
Self-assessed health	51	1.28
Chronic disease	70	1.75
Limitations in activities due to health problems	217	5.60
Canton	6	0.14

Note: N refers to number of respondents in estimation sample and % is percentage of estimation sample weighted for age, sex and district of residence.

Table A2. Characteristics of respondents with and without missing income data

	Missing income				
	No		Yes		
**Variable**	**N**	**(%)**	**N**	**(%)**	**p-value**
Sex					
Male	1,415	(77)	425	(23)	<0.001
Female	1,473	(68)	691	(32)	
Age					
15-24	221	(47)	247	(53)	<0.001
25-34	526	(77)	154	(23)	
35-44	588	(80)	151	(20)	
45-54	599	(74)	206	(26)	
55-64	480	(74)	172	(26)	
65 and over	474	(72)	186	(28)	
Marital status					
No	1,061	(69)	474	(31)	<0.001
Yes	1,763	(75)	583	(25)	
Immigrant					
No	1,757	(70)	751	(30)	<0.001
Yes	1,113	(76)	356	(24)	
Education					
Primary and pre-primary	268	(67)	133	(33)	<0.001
Secondary & post-secondary	1,514	(72)	581	(28)	
Tertiary	1,020	(77)	306	(23)	
Job Status					
Employed	1,619	(79)	429	(21)	<0.001
Unemployed	75	(75)	25	(25)	
Retired	616	(76)	194	(24)	
Student, domestic, compulsory service	345	(53)	301	(47)	
Permanently disabled and other inactive status	80	(69)	36	(31)	
Social support					
Low	108	(73)	39	(27)	0.95
Moderate	1,030	(73)	389	(27)	
High	1,716	(72)	656	(28)	
Informal Carer					
No	2,295	(73)	843	(27)	0.354
Yes	497	(71)	199	(29)	
BMI					
Normal or Underweight (<25)	1,407	(70)	603	(30)	0.002
Overweight (25-29)	904	(76)	290	(24)	
Obese (>=30)	530	(71)	212	(29)	
Smoker					
No	2,244	(71)	908	(29)	0.007
Occasionally	165	(72)	63	(28)	

Daily	450	(78)	130	(22)	
Alcohol consumption					
Never	622	(74)	220	(26)	0.054
Ex-drinkers	225	(67)	112	(33)	
Irregularly	301	(75)	98	(25)	
Regularly	948	(72)	370	(28)	
Everyday	714	(74)	251	(26)	
Self-assessed health					
Good/very good	1,952	(71)	784	(29)	0.065
Fair	721	(75)	242	(25)	
Bad/Very bad	191	(75)	63	(25)	
Chronic disease					
No	937	(71)	390	(29)	0.066
Yes	1,913	(73)	694	(27)	
Limitations in activities due to health problems					
No limitations	1,711	(72)	682	(29)	0.005
Limited/severely limited	1,055	(76)	339	(24)	
Canton					
Capellen	263	(69)	117	(31)	0.252
Clervaux	94	(72)	36	(28)	
Diekirch	123	(72)	49	(28)	
Echternach	72	(71)	29	(29)	
Esch zur Alzette	854	(73)	309	(27)	
Grevenmacher	170	(76)	54	(24)	
Luxembourg	835	(73)	316	(27)	
Mersch	165	(68)	76	(32)	
Redange	92	(63)	53	(37)	
Remich	113	(72)	45	(28)	
Vianden	23	(82)	5	(18)	
Wiltz	80	(76)	25	(24)	

Note: N refers to number of respondents and (%) is percentage of estimation sample weighted for age, sex and district of residence.

Table A3. Sensitivity analysis with dummy variable for non-reporting of income

	Wait (n=2,141)	Distance (n=2,000)	Could not afford medical care (n=2,348)	Could not afford dental care (n=2,465)	Could not afford prescribed mediciness (n=2,462)	Could not afford mental health care (n=1,672)
**Variable**						
Sex						
Male	1.00	1.00	1.00	1.00	1.00	1.00
Female	1.55 (1.25-1.92)***	0.81 (0.44-1.49)	0.80 (0.52-1.23)	0.94 (0.7-1.25)	0.54 (0.36-0.80)**	2.34 (1.29-4.25)**
Age						
15-24	1.00	1.00	1.00	1.00	1.00	1.00
25-34	1.13 (0.7-1.8)	0.29 (0.1-0.84)*	2.78 (0.99-7.82)	1.48 (0.78-2.80)	2.46 (1.04-5.84)*	3.28 (1.21-8.90)*
35-44	0.84 (0.51-1.37)	0.16 (0.05-0.51)**	2.94 (1.00-8.66)	1.25 (0.64-2.46)	1.26 (0.46-3.48)	1.34 (0.47-3.80)
45-54	0.76 (0.47-1.23)	0.09 (0.03-0.28)***	3.65 (1.28-10.41)*	1.42 (0.73-2.77)	2.49 (1.01-6.15)*	0.87 (0.29-2.59)
55-64	0.48 (0.29-0.79)**	0.14 (0.04-0.49)**	2.54 (0.80-8.12)	1.20 (0.58-2.45)	1.86 (0.68-5.12)	0.76 (0.24-2.45)
65 and over	0.37 (0.21-0.67)**	0.29 (0.09-0.95)*	2.16 (0.55-8.45)	0.87 (0.39-1.96)	2.14 (0.72-6.34)	0.17 (0.03-1.13)
Marital status						
No	1.00	1.00	1.00	1.00	1.00	1.00
Yes	1.13 (0.89-1.43)	1.13 (0.54-2.34)	0.64 (0.41-1.01)	0.77 (0.56-1.06)	0.56 (0.37-0.86)**	0.53 (0.29-0.96)*
Immigrant						
No	1.00	1.00	1.00	1.00	1.00	1.00
Yes	0.80 (0.64-1)	0.95 (0.5-1.82)	0.95 (0.63-1.44)	0.98 (0.72-1.33)	0.62 (0.40-0.96)*	1.41 (0.76-2.6)
Education						
Primary and pre-primary	1.00	1.00	1.00	1.00	1.00	1.00
Secondary & post-secondary (non-tertiary)	1.20 (0.8-1.82)	1.61 (0.58-4.46)	1.74 (0.81-3.72)	1.61 (0.94-2.78)	1.06 (0.59-1.91)	2.03 (0.65-6.38)
Tertiary	1.42 (0.9-2.24)	3.27 (1.04-10.23)*	0.93 (0.39-2.22)	1.21 (0.65-2.24)	0.59 (0.29-1.23)	1.74 (0.47-6.44)
Job Status						
Employed	1.00	1.00	1.00	1.00	1.00	1.00
Unemployed	0.95 (0.52-1.76)	0.40 (0.09-1.84)	2.04 (0.85-4.87)	1.65 (0.83-3.30)	1.68 (0.69-4.12)	2.84 (1.01-7.97)*
Retired	1.1 (0.72-1.66)	0.64 (0.23-1.79)	1.07 (0.47-2.47)	0.78 (0.45-1.36)	0.98 (0.47-2.05)	1.49 (0.35-6.34)
Student, domestic, compulsoryservice	1.11 (0.79-1.57)	0.54 (0.21-1.34)	1.10 (0.54-2.25)	1.11 (0.71-1.75)	1.82 (0.97-3.40)	2.50 (1.20-5.24)*
Permanently disabled and other inactive status	0.72 (0.38-1.39)	0.82 (0.18-3.73)	1.06 (0.44-2.59)	1.05 (0.55-2.02)	1.11 (0.50-2.48)	1.83 (0.61-5.46)
Social support (Low)						
Low	1.00	1.00	1.00	1.00	1.00	1.00
Moderate	1.17 (0.67-2.04)	0.59 (0.22-1.56)	0.48 (0.22-1.06)	0.43 (0.24-0.78)**	0.35 (0.17-0.72)**	0.19 (0.08-0.44)***
High	0.74 (0.43-1.3)	0.47 (0.18-1.26)	0.35 (0.16-0.77)**	0.40 (0.22-0.72)**	0.30 (0.15-0.60)**	0.07 (0.03-0.16)***
Household income						

Below 1st quintile (Lowest)	1.00	1.00	1.00	1.00	1.00	1.00
Between 1st & 2nd quintile	0.87 (0.59-1.28)	0.75 (0.32-1.75)	0.38 (0.18-0.78)**	0.79 (0.51-1.22)	0.50 (0.27-0.94)*	0.55 (0.23-1.29)
Between 2nd & 3rd quintile	1.05 (0.73-1.52)	0.20 (0.07-0.58)**	0.68 (0.37-1.24)	0.79 (0.51-1.23)	0.76 (0.44-1.32)	0.87 (0.39-1.96)
Between 3rd & 4th quintile	1.19 (0.81-1.76)	0.37 (0.12-1.12)	0.41 (0.19-0.87)*	0.48 (0.28-0.81)**	0.32 (0.16-0.67)**	0.52 (0.19-1.42)
Between 4th & 5th quintile (Highest)	0.97 (0.65-1.44)	0.16 (0.04-0.61)**	0.39 (0.17-0.87)*	0.30 (0.17-0.54)***	0.35 (0.17-0.75)**	0.20 (0.06-0.71)*
Income not reported	0.85 (0.60-1.21)	0.52 (0.24-1.14)	0.63 (0.36-1.12)	0.64 (0.42-0.97)*	0.69 (0.41-1.19)	0.80 (0.38-1.72)
Informal Carer						
No	1.00	1.00	1.00	1.00	1.00	1.00
Yes	1.36 (1.06-1.75)*	1.08 (0.55-2.09)	1.37 (0.87-2.15)	1.07 (0.75-1.52)	1.07 (0.69-1.66)	0.63 (0.31-1.28)
BMI						
Underweight or Normal	1.00	1.00	1.00	1.00	1.00	1.00
Overweight	0.83 (0.66-1.06)	0.84 (0.44-1.63)	0.53 (0.31-0.90)*	1.25 (0.91-1.72)	1.10 (0.69-1.74)	2.26 (1.20-4.24)*
Obese	0.98 (0.74-1.3)	1.41 (0.77-2.59)	0.98 (0.58-1.65)	1.32 (0.91-1.90)	1.57 (0.95-2.57)	1.61 (0.81-3.21)
Smoker (No)						
No	1.00	1.00	1.00	1.00	1.00	1.00
Occasionally	1.51 (0.99-2.29)	1.92 (0.85-4.31)	2.24 (1.13-4.45)*	0.80 (0.43-1.48)	0.31 (0.09-1.06)	0.27 (0.06-1.24)
Daily	0.95 (0.71-1.27)	0.91 (0.43-1.9)	1.31 (0.81-2.12)	1.72 (1.25-2.38)**	1.04 (0.66-1.64)	2.04 (1.13-3.69)*
Alcohol consumption						
Never	1.00	1.00	1.00	1.00	1.00	1.00
Ex-drinkers	1.30 (0.87-1.94)	0.80 (0.32-1.97)	1.57 (0.78-3.19)	1.09 (0.67-1.77)	1.19 (0.6-2.36)	1.77 (0.74-4.21)
Irregularly	1.05 (0.71-1.56)	1.11 (0.46-2.65)	1.18 (0.57-2.42)	0.81 (0.5-1.33)	1.27 (0.69-2.37)	1.43 (0.57-3.61)
Regularly	1.16 (0.87-1.55)	0.54 (0.24-1.19)	1.00 (0.59-1.68)	0.60 (0.42-0.87)**	0.86 (0.52-1.41)	1.42 (0.69-2.93)
Everyday	1.07 (0.79-1.45)	0.70 (0.3-1.63)	0.94 (0.51-1.74)	0.78 (0.53-1.17)	0.91 (0.53-1.57)	1.18 (0.52-2.67)
Self-assessed health						
Good/very good	1.00	1.00	1.00	1.00	1.00	1.00
Fair	1.10 (0.85-1.44)	3.5 (1.88-6.53)***	1.41 (0.83-2.4)	1.52 (1.07-2.15)*	0.88 (0.55-1.41)	1.36 (0.68-2.73)
Bad/Very bad	1.86 (1.25-2.79)**	7.88 (3.71-16.73)***	3.76 (1.89-7.48)***	2.66 (1.61-4.39)***	1.67 (0.85-3.29)	2.40 (0.99-5.84)
Chronic disease (No)						
No	1.00	1.00	1.00	1.00	1.00	1.00
Yes	1.45 (1.14-1.84)**	1.09 (0.53-2.22)	0.77 (0.46-1.29)	0.90 (0.65-1.26)	0.75 (0.48-1.18)	2.04 (0.96-4.35)
Limitations in activities due to health problems						
No limitations	1.00	1.00	1.00	1.00	1.00	1.00
Limited/severely limited	1.56 (1.23-1.97)***	1.30 (0.72-2.35)	1.24 (0.74-2.10)	1.11 (0.80-1.55)	1.56 (0.99-2.45)	2.17 (1.08-4.35)*
Canton						
Esch zur Alzette	1.00	1.00	1.00	1.00	1.00	1.00
Capellen	1.12 (0.78-1.60)	1.17 (0.34-4.01)	1.12 (0.54-2.33)	1.09 (0.67-1.78)	1.33 (0.7-2.52)	1.50 (0.62-3.68)
Clervaux	0.81 (0.45-1.46)	3.63 (1.14-11.52)*	0.96 (0.31-2.92)	0.64 (0.27-1.48)	1.40 (0.56-3.49)	0.29 (0.05-1.68)

Diekirch	0.94 (0.54-1.62)	3.92 (1.27-12.04)*	2.04 (0.89-4.67)	1.17 (0.59-2.33)	2.06 (0.91-4.68)	1.58 (0.41-6.13)
Echternach	1.22 (0.64-2.31)	2.63 (0.64-10.84)	1.81 (0.59-5.60)	1.21 (0.59-2.46)	1.20 (0.38-3.81)	0.64 (0.06-6.66)
Grevenmacher	1.38 (0.89-2.16)	2.27 (0.77-6.66)	1.07 (0.42-2.72)	1.47 (0.83-2.59)	2.12 (1.03-4.35)*	1.53 (0.48-4.86)
Luxembourg	1.17 (0.90-1.53)	1.48 (0.68-3.23)	0.77 (0.46-1.31)	0.94 (0.66-1.35)	1.01 (0.60-1.68)	1.60 (0.83-3.10)
Mersch	1.75 (1.13-2.72)*	0.88 (0.18-4.39)	1.65 (0.71-3.85)	1.00 (0.51-1.92)	2.04 (1.00-4.16)	0.51 (0.10-2.54)
Redange	0.96 (0.56-1.63)	1.37 (0.25-7.53)	1.31 (0.53-3.28)	0.53 (0.22-1.30)	0.60 (0.18-2.03)	0.67 (0.13-3.34)
Remich	1.02 (0.57-1.84)	3.70 (1.1-12.5)*	0.66 (0.21-2.06)	0.85 (0.41-1.73)	1.30 (0.53-3.15)	3.64 (1.07-12.36)*
Vianden	0.49 (0.12-1.90)	6.99 (0.65-75.66)	0.69 (0.12-3.86)	0.91 (0.27-3.06)	2.37 (0.77-7.31)	1.69 (0.28-10.20)
Wiltz	0.57 (0.27-1.21)	1.44 (0.28-7.35)	1.74 (0.44-6.81)	0.98 (0.42-2.27)	1.43 (0.38-5.37)	0.93 (0.16-5.38)
Constant	0.22 (0.09-0.51)**	0.12 (0.02-0.73)*	0.07 (0.01-0.30)***	0.28 (0.10-0.80)*	0.25 (0.08-0.84)*	0.02 (0.00-0.11)***

Note: Estimates are weighted for age, sex and district of residence. ***p < 0.001.**p < 0.01.*p < 0.05. Data are odds ratios adjusted for explanatory variables (95% confidence intervals)
