# Supplementary material for: Investigating unmet need for healthcare using the European Health Interview Survey: a cross-sectional survey study of Luxembourg
Source: BMJ Open. 2021 Aug 3;11(8):e048860. doi: 10.1136/bmjopen-2021-048860 (PMC8336210; doi:10.1136/bmjopen-2021-048860)
Supplement: Author's manuscript [file bmjopen-2021-048860.draft_revisions.pdf]

BMJ Open is committed to open peer review. As part of this commitment we make the peer review history of every article we publish publicly available.

When an article is published we post the peer reviewers' comments and the authors' responses online. We also post the versions of the paper that were used during peer review. These are the versions that the peer review comments apply to.

The versions of the paper that follow are the versions that were submitted during the peer review process. They are not the versions of record or the final published versions. They should not be cited or distributed as the published version of this manuscript.

BMJ Open is an open access journal and the full, final, typeset and author-corrected version of record of the manuscript is available on our site with no access controls, subscription charges or pay-per-view fees (http://bmjopen.bmj.com).

If you have any questions on BMJ Open's open peer review process please email info.bmjopen@bmj.com

BMJ Open

Investigating unmet need for health care using the European Health Interview Survey: a cross-sectional survey study of Luxembourg

Journal:	*BMJ Open*
Manuscript ID	bmjopen-2021-048860
Article Type:	Original research
Date Submitted by the Author:	11-Jan-2021

Complete List of Authors:	Moran, Valerie; Luxembourg Institute of Health; Luxembourg Institute of Socio-Economic Research,<br>Suhrcke, Marc; Luxembourg Institute of Socio-Economic Research,<br>Ruiz-Castell, Maria; Luxembourg Institute of Health,<br>Barré, Jessica; Caisse nationale de santé<br>Huiart, Laetitia; Santé publique France

Keywords:	Health economics < HEALTH SERVICES ADMINISTRATION & MANAGEMENT, Health policy < HEALTH SERVICES ADMINISTRATION & MANAGEMENT, International health services < HEALTH SERVICES ADMINISTRATION & MANAGEMENT, Organisation of health services < HEALTH SERVICES ADMINISTRATION & MANAGEMENT

SCHOLARONE™
Manuscripts

I, the Submitting Author has the right to grant and does grant on behalf of all authors of the Work (as defined in the below author licence), an exclusive licence and/or a non-exclusive licence for contributions from authors who are: i) UK Crown employees; ii) where BMJ has agreed a CC-BY licence shall apply, and/or iii) in accordance with the terms applicable for US Federal Government officers or employees acting as part of their official duties; on a worldwide, perpetual, irrevocable, royalty-free basis to BMJ Publishing Group Ltd ("BMJ") its licensees and where the relevant Journal is co-owned by BMJ to the co-owners of the Journal, to publish the Work in this journal and any other BMJ products and to exploit all rights, as set out in our licence.

The Submitting Author accepts and understands that any supply made under these terms is made by BMJ to the Submitting Author unless you are acting as an employee on behalf of your employer or a postgraduate student of an affiliated institution which is paying any applicable article publishing charge ("APC") for Open Access articles. Where the Submitting Author wishes to make the Work available on an Open Access basis (and intends to pay the relevant APC), the terms of reuse of such Open Access shall be governed by a Creative Commons licence – details of these licences and which Creative Commons licence will apply to this Work are set out in our licence referred to above.

Other than as permitted in any relevant BMJ Author's Self Archiving Policies, I confirm this Work has not been accepted for publication elsewhere, is not being considered for publication elsewhere and does not duplicate material already published. I confirm all authors consent to publication of this Work and authorise the granting of this licence.

For peer review only

Investigating unmet need for health care using the European Health Interview Survey: a cross-sectional survey study of Luxembourg

Valerie Moran*[1,2], Marc Suhrcke[2], Maria Ruiz-Castell[1], Jessica Barré[3], Laetitia Huiart[4].

[1] Luxembourg Institute of Health (LIH), Strassen, Luxembourg
[2] Luxembourg Institute of Socio-Economic Research (LISER), Esch-sur-Alzette, Luxembourg
[3] Caisse national de santé, Luxembourg, Luxembourg
[4] Santé Publique, Paris, France

*Corresponding author:
Valerie Moran
Valerie.Moran@lih.lu

For peer review only

Abstract

Objectives: We illustrate the value of the European Health Interview Survey (EHIS) to investigate the prevalence and determinants of unmet need in a more thorough way than other more widely used surveys have allowed. We use Luxembourg as an interesting and policy-relevant case study.

Design: Cross-sectional survey conducted between February and December 2014. We investigated associations between the outcome and independent variables using multivariate logistic regression models.

Setting and participants: 4,004 members of the resident population in private households in Luxembourg aged 15 years and over.

Outcome measures: We created six binary variables to measure unmet need arising from wait time, distance and affordability of medical care, dental care, prescribed medicines and mental health care among those who reported a need for care.

Results: The most common barrier to access arose from wait times (32%) and the least common from distance/transportation (4%). Dental care (12%) was most commonly cited as being unaffordable, followed by prescribed medicines (6%), medical (5%) and mental health care (5%). Respondents who reported bad or very bad health were associated with every component of unmet need compared to those with good or very good health (wait: OR 2.41, 95% CI 1.53-3.80, distance/transportation: OR 7.12 95% CI 2.91-17.44, afford medical care: OR 5.35 95% CI 2.39-11.95, afford dental care: OR 3.26 95% CI 1.86-5.71, afford prescribed medicines: OR 2.22 95% CI 1.04-4.71, afford mental health care: OR 3.58 95% CI 1.25-10.30).

Conclusions: Compared to other European surveys, the EHIS facilitates separate analyses of unmet need arising from long waits, distance or transportation problems, and affordability of medical, dental and mental health care and prescribed medicines. This study demonstrates the EHIS is a useful resource that could be utilised for future within- and between-country analyses of unmet need for health care in the general population in European countries.

For peer review only

Strengths and limitations of this study

We investigate unmet need using a representative cross-sectional population based survey – the European Health Interview Survey (EHIS).

The EHIS allows us to investigate unmet need due to the affordability of prescribed medications and mental health care, in addition to medical and dental care.

As unmet need is self-reported it may be subject to recall bias or response bias.

The EHIS does not investigate unmet need due to wait for different types of services.

This study explores associations between unmet need and socio-demographic, health status and risk factor variables but does not establish causality.

For peer review only

For peer review only - http://bmjopen.bmj.com/site/about/guidelines.xhtml

Introduction

In recent years, the concept of universal health coverage (UHC) has emerged as a key objective of international organisations including the WHO, OECD and World Bank, who support national governments to achieve this goal. UHC is defined as "ensuring that all people have access to needed health services…..of sufficient quality to be effective while also ensuring that the use of these services does not expose the user to financial hardship" (1). Therefore, access to care is a core component of UHC. Barriers to accessing health care arising from the cost, physical accessibility and quality of services can lead to unmet need at an individual level due to affordability, distance and waiting times (2, 3). Survey data is commonly used to ascertain individuals' perceptions of unmet need arising from various barriers to accessing care (4, 5).

To date, studies that investigated unmet need within and across European countries have used data from the European Union Statistics on Income and Living Conditions (EU-SILC) (6-17), the European Social Survey (ESS) (18, 19) and the European Health Interview Survey (EHIS) (20-22). The studies using the EHIS undertook analyses in a national context but focused on very specific sub-groups rather than comprehensive population-level analysis.The EHIS differs from the EU-SILC and ESS as it does not ask respondents a binary (yes/no) question on whether they have unmet need. Rather, the EHIS asks respondents to consider unmet need arising from specific barriers to accessing health care, including long waits, distance or transportation problems and the affordability of services. The EHIS data allows the investigation of each component of unmet need separately and the consideration of financial barriers for medical, dental and mental health care and prescribed medicines. The ESS only collects data on unmet need for medical care and while the EU-SILC contains questions on unmet need for medical and dental care, it does not ask respondents about unmet need for prescribed medicines or mental health care. In this paper, we illustrate the value of using EHIS data for the purpose of investigating the prevalence and determinants of unmet need related to different barriers to access, in a potentially more thorough way than other more widely surveys have allowed. We use Luxembourg as a case study to demonstrate the usefulness of EHIS data. Luxembourg offers an interesting policy case that allows us to examine whether unmet need varies for services where patients face different cost barriers i.e. prescribed medicines compared to medical, dental and mental health care.

For peer review only - http://bmjopen.bmj.com/site/about/guidelines.xhtml

Compared to other EU countries, Luxembourg has high per capita public health care expenditures, a low share of total health spending financed by out-of-pocket payments and a comprehensive benefit package (23, 24). Similar to its neighbouring countries of Belgium, France and Germany, Luxembourg provides universal coverage of health care through a social health insurance system, the *Caisse Nationale de Santé* (CNS). Registration with the CNS is mandatory for those who are economically active or receive a substitutive income (25). Uninsured family members can benefit from coverage through their affiliation with an insured family member (26). In 2018, the CNS covered 93% of the resident population (27, 28). The proportion not covered included EU officials based in Luxembourg, who were insured under a separate scheme provided by their employer (25) and vulnerable populations including the homeless and irregular immigrants and their families (29). Luxembourg is among a minority of countries (including Belgium and France) in the EU where patients pay ambulatory care providers directly and then claim reimbursement from the social health insurance fund (30). In Belgium and France, specific population groups including those on low incomes or with a chronic illness have their costs directly covered by the social insurance fund, a scheme known as the '*tiers payant'* or third party payment system (31, 32). In Luxembourg, when patients access health care including doctor consultations in outpatient or inpatient settings, dental and paramedical services, they must pay providers the full cost upon receipt of care and then apply for a refund from the CNS for the covered share of the payment (excluding co-payments). The CNS directly reimburses providers for hospital services (excluding the doctors' fees), laboratory tests and pharmaceuticals, leaving the patient to pay only the co-payment at the point of use (33). Co-payments differ for different services. Hospital treatment is covered at 100% (except for a per diem of €22.54, with an exemption for childbirth) and outpatient treatment at 88% for adults. Dental care is covered at a rate of 88% for adults after an annual amount of €60, which is paid by the CNS and is covered at a rate of 100% for those under 18 years. Medications eligible for CNS coverage are reimbursed at a rate of 40%, 80% or 100% based on criteria such as severity of illness, availability of substitutes and financial burden for the patient (25, 34).

The payment of the full cost of medical, dental and mental health care by the patient upon receipt of care may create a financial barrier to accessing care for certain groups, for example, those on lower incomes (29). In 2013, the government introduced the system of 'third-party social payment' ('*tiers payant social*'), which entitles people in economic hardship to request assistance with the payment of health care expenses (35). Eligible patients are exempted from the payment of costs and the CNS reimburses the provider. The local social welfare office covers any co-payments the

For peer review only - http://bmjopen.bmj.com/site/about/guidelines.xhtml

patient cannot afford. Following a national debate over recent years, the government announced in November 2019 that a universal system of third-party payment would be introduced (36). In addition to financial access to care, waiting times for health services have been identified as an important policy issue in Luxembourg. Waiting times are commonly a pertinent issue in countries with a national health system funded by general taxation. However, a recent report highlighted that waiting times were a medium to high priority in Luxembourg, in contrast to its neighbouring countries of Belgium and Germany, where waiting times were a low to medium or low priority (37).

Data and methods

The EHIS is a cross-sectional observational survey undertaken in all EU countries. Patients or the public were not involved in the design, conduct, reporting or dissemination of the EHIS. Information on the 2014 EHIS for Luxembourg, including the questionnaire and data access procedure is available on the Ministry of Health website (38). The survey collects information on health status, health determinants, utilisation of and barriers to access to health care and sociodemographic characteristics (39). The coverage of the survey includes the resident population in private households aged 15 years and over. A one-stage random sample stratified by age, sex and district of residence (Luxembourg, Diekirch and Grevenmacher) was drawn from the registry of CNS insurees (38, 40). Among the 16,000 individuals invited to participate, 4,823 (30.1%) responded by submitting an electronic (70%) or paper (30%) questionnaire (39). Of these respondents, 4,118 participants met the inclusion criteria, provided informed consent and completed the questionnaire. Data were collected between February and December 2014 (40). The final database comprises 4,004 individuals who completed more than 50% of the questionnaire and had no missing data for age, sex or district, giving a participation rate of 25%. This database was prepared according to a European protocol (41) and was validated by Eurostat (38).

The EHIS questions ask respondents to consider unmet need arising from barriers to accessing health care due to delay and affordability among those who report a need for health care. The survey questions are available in Appendix 1 of the online supplementary material. For our dependent variables, we created six binary variables to measure barriers to access due to wait time, distance and affordability of medical care, dental care, prescribed medicines and mental health care among those who reported a need for care. We coded these variables as one, if respondents replied “Yes” and zero if respondents replied “No”.

For peer review only - http://bmjopen.bmj.com/site/about/guidelines.xhtml

Independent variables cover socio-demographics, health behaviours and health status. Socio-demographic variables include sex, age, marital status, immigrant, education, employment status, income, social support and being an informal carer. Sex, marital status, immigrant and informal carer are constructed as binary variables while age, education and employment status are constructed as categorical variables. Income is measured in quintiles and also included as a categorical variable. Following Ruiz-Castell et al. (2017) (42), we created a categorical variable on low, moderate and high social support using the questions: "how much concern do people show in what you are doing?" and "how easy is it to get practical help from neighbors if you should need it?". Health risk factors include Body Mass Index (BMI), smoking and alcohol consumption. We considered alcohol consumption as 'irregular', if it occurred at most 2-3 days per month, and 'regular', if it occurred at least once per week. We use three measures of health status: self-assessed health, presence of a chronic disease and limitations in activities due to health problems. We also include binary variables for the twelve cantons (see Figure 1) to capture geographical differences in unmet need.

Insert Figure 1 around here

We conduct separate analyses for each component of unmet need and investigate associations between the outcome and independent variables using multivariate logistic regression models. As a sensitivity analysis, following Bataineh et al. (43), we included a binary variable equal to one for observations with missing data for income in all models. We use sample weights to adjust for age, sex and district of residence. We estimated models with robust standard errors. We report results as odds ratios with 95% confidence intervals. We estimated all models using Stata Version 15 (44).

Results

For each component of unmet need, we excluded respondents who reported no need for health care. This ranged from 15% of observations for dental care and prescribed medicines to 38% for mental health care (See Table A1 in Appendix 2 of the online supplementary data). We also excluded observations with missing data. Table A2 in Appendix 2 of the online supplementary material shows the percentage of missing data for each independent variable. Missing data for the independent variables did not exceed 7%, except for income, where 29% of observations had missing data. Table A3 in Appendix 2 shows the characteristics of respondents with missing income data. These were more likely to be female, aged 15-24, unmarried, have Luxembourgish nationality, have only primary and pre-primary education, be a student, fulfilling domestic tasks or

For peer review only - http://bmjopen.bmj.com/site/about/guidelines.xhtml

in compulsory service, have no limitations in activities due to a health problem and were less likely to be overweight or smoke daily.

Table 1 shows the sample size for each component of unmet need and the weighted percentage of unmet need. Over one-third (35%) of respondents report unmet need due to any component (long waits, distance or transportation or affordability of care). The most commonly cited barrier to access was due to wait times (32%) while the least common was distance (4%). 15% of respondents reported unmet need due to affordability of care. Dental care (12%) was most commonly cited as being unaffordable, followed by prescribed medicines (6%), medical (5%) and mental health care (5%).

Insert Table 1 around here

Table 2 presents the number and weighted percentage of respondents according to the independent variables for each component of unmet need. Respondents who had bad or very bad health, a chronic disease, limitations in activities due to health problems or were ex-drinkers were more likely to report unmet need for all components. Respondents with obesity, low social support, and whose income fell below the first quintile were more likely to report unmet need due to distance and financial barriers. There was no discernible pattern between the remaining respondent characteristics and types of unmet need.

Insert Table 2 around here

Table 3 displays the results of the logistic regression models. Respondents who reported bad or very bad health were associated with a higher risk of experiencing unmet need for every component, compared to those with good or very good health. Variables positively associated with reporting unmet need due to wait times included being female (OR 1.52, 95% CI 1.19-1.93), having a chronic condition (OR 1.45, 95% CI 1.11-1.91) and limitations in activities due to health problems (OR 1.33, 95% CI 1.02-1.73). Respondents aged 65 years and over were less likely to experience long wait times in comparison to the 15-24 age group (OR 0.44, 95% CI 0.21-0.90). A lower risk of experiencing delays due to distance or transportation problems was associated with the age groups 35-44 (OR 0.20, 95% CI 0.05-0.75), 45-54 (OR 0.04, 95% CI 0.01-0.23) and 55-64 (OR 0.14, 95% CI 0.03-0.63) as well as the income groups between the second and third quintiles (OR 0.19, 95% CI 0.06-0.54) and the fourth and fifth quintiles (OR 0.14, 95% CI 0.04-0.57). There is some consistency in determinants of unmet need arising from financial barriers for different types of care. Moderate or high social support was associated with a lower risk for dental

For peer review only - http://bmjopen.bmj.com/site/about/guidelines.xhtml

care, prescribed medicines and mental health care. Married respondents were also associated with lower risk for prescribed medicines (OR 0.53, 95% CI 0.33-0.84) and mental health care (OR 0.40, 95% CI 0.21-0.79). Non-participation in the labour force due to studies, domestic work or compulsory service was associated with a higher risk of being unable to afford prescribed medicines (OR 2.22, 95% CI 1.04-4.76) and mental health care (OR 2.95, 95% CI 1.28-6.77). Income between the first and second quintiles were associated with a lower risk of unmet need for medical care (OR 0.37, 95% CI 0.18-0.78) and prescribed medicines (OR 0.49, 95% CI 0.26-0.95); income between the third and fourth quintiles was associated with a lower risk for dental care (OR 0.46, 95% CI 0.26-0.79) and prescribed medicines (OR 0.33, 95% CI 0.15-0.70); and income between the fourth and fifth quintiles was associated with a lower risk for dental care (OR 0.29, 95% CI 0.16-0.53), prescribed medicines (OR 0.38, 95% CI 0.17-0.82) and mental health care (OR 0.17, 95% CI 0.05-0.61). Daily smokers were associated with a higher risk of unmet need for dental (OR 1.82, 95% CI 1.28-2.60) and mental health care (OR 2.15, 95% CI 1.10-4.17) compared to non-smokers. Immigrants were at higher risk of being unable to afford prescribed medicines (OR 0.58, 95% CI 0.35-0.97).

Insert Table 3 around here

Table A4 in Appendix 2 of the supplementary data shows the results of the sensitivity analysis that includes a binary variable measuring income non-response in each model. This variable was statistically significant in only the model for affordability of dental care (OR 0.64, 95% CI 0.42-0.97) and results for all models were largely unchanged.

Discussion

In this paper, we demonstrate the use of EHIS data to measure unmet need in the general population, using Luxembourg as an illustrative and policy-relevant case study. The most common barrier to accessing health care arose from long waits (32%). Compared to other countries who collect this data using the EHIS, Luxembourg has the highest proportion of respondents who report unmet need due to long waits, comparable to Italy (30%) (45). Respondents were most likely to report being unable to afford dental care (12%) followed by prescribed medicines (6%) – a result that is on par with the EU averages (12.3% and 4.6% respectively) (46). In 2017, over 50% of total out-of-pocket spending on health in Luxembourg was on pharmaceuticals and other medical goods along with dental services (47). The percentage of respondents that reported being unable to afford mental health care (5%) was higher than the

For peer review only - http://bmjopen.bmj.com/site/about/guidelines.xhtml

EU average (2.7%) (46). The finding for Luxembourg may be driven in part by the non-reimbursement of visits to psychologists by the CNS.

Our study is subject to limitations arising from our data, some of which could in principle be overcome in future research, via development and adjustment of the EHIS questionnaire. As unmet need is self-reported, the data may suffer from the limitations inherent in survey data, including recall or response bias (respondent provide erroneous responses or misconstrue the question) (48, 49). Moreover, self-reported unmet need may be influenced by unobservable factors such as cultural norms, heath literacy and expectations of health services (7, 12, 50, 51). The survey sampling did not include sections of society who lack any insurance coverage and may have high unmet need including the homeless and irregular immigrants and their families (29). Individuals living in collective households and institutions were also excluded. Despite these limitations, survey data remains the best available source (50). Respondents with missing data on income were more likely to be young (15-24 years), with only primary and pre-primary education, and be students or fulfilling unpaid domestic tasks or compulsory service, implying they had low incomes. Therefore, disparities in unmet need arising from affordability of care may be underestimated.

Previous studies (6, 8, 13, 15, 17) have investigated unmet need within countries using EU-SILC data. The use of the EHIS brings two important advantages that move us beyond these studies. First, we undertake separate analyses of unmet need arising from long waits, distance or transportation problems, and affordability of medical, dental and mental health care and prescribed medicines. Second, we exploit a richer set of health variables, which enables us to investigate risk factors including BMI, smoking and alcohol consumption as determinants of unmet need. Moreover, this is the first study to use the EHIS to investigate the determinants of unmet need in the general population. Previous studies using the EHIS to investigate unmet need focused on specific population groups. Sakellariou et al. (22) used the EHIS to investigate if people with disabilities had higher unmet need for health care due to waiting times, distance or transport problems and unaffordability of medical and mental health care and prescribed medicines compared to people without disabilities in the UK. Rotarou et al. (21) conducted a similar study for Greece. Hoebel et al. (20) focused only on respondents aged 50-85 years of age in Germany to investigate unmet need due to the affordability of medical, dental and mental health care and prescribed medicines. They did not consider long waits or distance or transportation problems.

For peer review only - http://bmjopen.bmj.com/site/about/guidelines.xhtml

Three previous studies (6, 11, 15) used EU-SILC data on reasons for unmet need to investigate associations with various determinants. Two studies (6, 15) considered the main reason for unmet need due to availability (waiting lists) and accessibility (affordability and distance/transportation) while the remaining study (11) considered affordability and distance/transportation as separate categories. Comparable to our results, these studies reported a positive association between bad and very bad health (compared to very good) and unmet need due to availability (6) and accessibility (6, 15) or only affordability of care (11). One study (11) found that social support as measured by the ability to seek help was associated with a lower probability of unmet needs for medical care arising from economic costs, reflecting our finding that higher levels of social support were associated with lower unmet need due to affordability of care. Our result that females were associated with higher unmet need due to wait times was not supported by these studies, which found evidence of higher unmet need for females due to accessibility (6) and affordability of care (11). Women and respondents with bad or very bad self-assessed health may be unable to afford mental health care because they have higher need. A previous study (42) investigated the burden of depression in Luxembourg and reported a higher prevalence rate of depression symptoms in females compared to males and in those who perceived their health as poor, compared to those who perceived their health as good. This study also found that good social support was associated with a lower risk of depression, which could indicate a lower need for mental health care and hence lower unmet need arising from the affordability of mental health care.

Similar to previous studies (6, 15), we find that higher income is associated with a lower risk of unmet need due to distance and affordability of services. Income disparities in unmet need due to the affordability of services may reflect to some extent the need for patients to pay the full costs of many services directly to providers at the point of use. Our finding of no statistically significant relationship between income and unmet need due to waits is also reflected in previous studies (6, 8, 11, 15). The EHIS data allows us to consider the association between health behaviours and unmet need. We find that these variables were only associated with unmet need arising from the affordability of health care. Therefore, we add to a previous study from Canada (43) that considered the relationship between obesity, drinking and smoking and unmet need due to health system factors (including unavailability of services and long waiting times and excluding cost) and found evidence of a positive association between smoking (both daily and occasional) and unmet need.

The Luxembourgish health system is characterised by a high rate of population health insurance coverage, a comprehensive benefit package and a relatively low level of out-of-pocket payments.

For peer review only - http://bmjopen.bmj.com/site/about/guidelines.xhtml

Nevertheless, access to health care is an important issue, particularly in relation to wait times, as reflected in our finding that the majority of respondents reported unmet need due to long waits. Data on wait times for health care in Luxembourg are not routinely published. However, a recent report (52) revealed that waiting times are a medium to high priority in Luxembourg and are an issue across different types of services including specialist care, diagnostic tests, hospital emergency departments, primary care and cancer care. A 2016 study revealed an average wait of almost four hours between admission to and discharge from emergency services, which are provided by each of the four general hospitals. The majority (75%) of attendees experienced a wait of three hours or less, just below the government target of 85% (53). These findings prompted the government to set a maximum waiting time target of 2.5 hours for emergency services (54). This compares to a target of a maximum four-hour wait in accident and emergency departments from arrival to admission, transfer or discharge in England (55).

Efforts have also been undertaken to improve the organisation of cancer care in order to reduce waiting times. In 2016, the government embarked on a reform of the National Health Laboratory's diagnostic services by reducing outsourcing to other countries and concentrating the delivery of these services in Luxembourgish hospitals instead. The government also introduced maximum waiting time targets for cancer care. At least 95% of patients should receive a diagnosis within five working days while specific targets are in place for different types of cancer (e.g. a maximum of four weeks between chemotherapy and radiotherapy or two weeks following receipt of the analytical report) (52). If residents of Luxembourg enrolled in the CNS perceive wait times as too long, they may seek health care in another EU state. Prior authorisation from the CNS is not required for a doctor consultation (in a health centre, clinic or hospital) but is required if the consultation uses specialised medical equipment or hospital infrastructure or for inpatient treatments with at least one overnight stay. The CNS may withhold authorisation if the necessary treatment can be provided in Luxembourg within a medically justifiable timeframe (56).

The EHIS does not ask respondents if they perceived long waits across the health care system or for particular services (primary care, cancer care, elective treatment, diagnostic tests), nor the length of time perceived as long. Future research could investigate the issue of waiting times in Luxembourg for different types of services in order to inform policy measures to reduce unmet need. Recent policies focused on the reorganisation of cancer care delivery and introduction of waiting time targets for cancer care could be evaluated. Given our finding of income disparities in unmet need due to the affordability of care, an evaluation of the implementation of the third-party payment system to date would inform its planned expansion and potential further adjustments.

For peer review only - http://bmjopen.bmj.com/site/about/guidelines.xhtml

The collection of a third wave of EHIS data will enable an assessment of trends in unmet need in Luxembourg over time. This research also demonstrates that the EHIS is a useful resource that could be utilised for future within- and between-country analyses of unmet need for health care in the general population in European countries. While previous cross-country studies have examined patient and health system characteristics associated with unmet need across European countries for medical and dental care, the EHIS could complement these studies by extending the analyses to unmet need arising from the affordability of mental health care and prescribed medicines.

For peer review only

For peer review only - http://bmjopen.bmj.com/site/about/guidelines.xhtml

Authors' contribution:

Valerie Moran: Conceptualisation, Methodology, Software, Formal Analysis, Writing – Original Draft, Writing – Review and Editing, Visualisation. **Marc Suhrcke:** Writing – Review and Editing, **Maria Ruiz-Castell:** Writing – Review and Editing, Software, Resources, **Jessica Barré:** Data curation, **Laetitia Huiart:** Conceptualisation, Writing – Review and Editing, Supervision.

Funding – The EHIS Luxembourg study was funded by Luxembourg's Directorate and Ministry of Health and the Luxembourg Institute of Health. There is no associated grant number.

Acknowledgements: We would like to thank to the population of Luxembourg and all the EHIS team who have contributed to this study. We would like to thank EHIS steering committee members S Couffignal, N de Rekeneire, S Leite, G Weber, AC Lorcy, G Osier and I Salagean for their valuable contributions.

Competing interests: Jessica Barré was formerly an employee of the Luxembourg Institute of Health and is currently employed at the *Caisse National de Santé* (CNS). The views presented are those of the authors alone and not of the CNS.

Data Sharing Statement: Information on the data access procedure for the 2014 EHIS for Luxembourg is available on the Ministry of Health website: https://sante.public.lu/fr/statistiques/ehis/ehis-methodologie/ehis-formulaire-demande-de-donnees.docx

Ethics approval: Luxembourg's National Ethics Committee, the Comité National d'Ethique de Recherche (CNER) was informed by the Luxembourg Ministry of Health of the national regulation and European obligation to collect EHIS data. The survey was completely anonymous and no information on name, address or date of birth of respondents was collected.

For peer review only

References

1. WHO. Health systems: Universal health coverage: WHO; 2020 [Available from: https://www.who.int/healthsystems/universal_health_coverage/en/.
2. Gulliford M, Figueroa-Munoz J, Morgan M, Hughes D, Gibson B, Beech R, et al. What does 'access to health care' mean? J Health Serv Res Policy. 2002;7(3):186-8.
3. European Union. EXPH (EXpert Panel on effective ways of investing in Health). Opinion on Benchmarking Access to Healthcare in the EU. Brussels: European Union; 2017.
4. European Union. Expert Panel on effective ways of investing in Health (EXPH), Report on Access to Health Services in the European Union. Brussels: European Commission; 2016.
5. OECD. Health for Everyone?: Social Inequalities in Health and Health Systems. Paris: OECD Publishing; 2019.
6. Cavalieri M. Geographical variation of unmet medical needs in Italy: a multivariate logistic regression analysis. Int J Health Geogr. 2013;12:27-.
7. Chaupain-Guillot S, Guillot O. Health system characteristics and unmet care needs in Europe: an analysis based on EU-SILC data. The European journal of health economics : HEPAC : health economics in prevention and care. 2015;16(7):781-96.
8. Connolly S, Wren M-A. Unmet healthcare needs in Ireland: Analysis using the EU-SILC survey. Health Policy. 2017;121(4):434-41.
9. Detollenaere J, Hanssens L, Vyncke V, De Maeseneer J, Willems S. Do We Reap What We Sow? Exploring the Association between the Strength of European Primary Healthcare Systems and Inequity in Unmet Need. PLoS One. 2017;12(1):e0169274-e.
10. Elstad JI. Income inequality and foregone medical care in Europe during The Great Recession: multilevel analyses of EU-SILC surveys 2008-2013. Int J Equity Health. 2016;15(1):101-.
11. Fiorillo D. Reasons for unmet needs for health care: the role of social capital and social support in some western EU countries. International journal of health economics and management. 2019.
12. Israel S. How social policies can improve financial accessibility of healthcare: a multi-level analysis of unmet medical need in European countries. Int J Equity Health. 2016;15:41.
13. Legido-Quigley H, Karanikolos M, Hernandez-Plaza S, de Freitas C, Bernardo L, Padilla B, et al. Effects of the financial crisis and Troika austerity measures on health and health care access in Portugal. Health Policy. 2016;120(7):833-9.
14. Madureira-Lima J, Reeves A, Clair A, Stuckler D. The Great Recession and inequalities in access to health care: a study of unemployment and unmet medical need in Europe in the economic crisis. International journal of epidemiology. 2018;47(1):58-68.
15. Popovic N, Terzic-Supic Z, Simic S, Mladenovic B. Predictors of unmet health care needs in Serbia; Analysis based on EU-SILC data. PLoS One. 2017;12(11):e0187866.
16. Reeves A, McKee M, Mackenbach J, Whitehead M, Stuckler D. Public pensions and unmet medical need among older people: cross-national analysis of 16 European countries, 2004-2010. Journal of epidemiology and community health. 2017;71(2):174-80.
17. Yardim MS, Uner S. Equity in access to care in the era of health system reforms in Turkey. Health Policy. 2018;122(6):645-51.
18. Fjær EL, Stornes P, Borisova LV, McNamara CL, Eikemo TA. Subjective perceptions of unmet need for health care in Europe among social groups: Findings from the European social survey (2014) special module on the social determinants of health. European journal of public health. 2017;27(suppl_1):82-9.
19. Quintal C, Lourenço Ó, Ramos LM, Antunes M. No unmet needs without needs! Assessing the role of social capital using data from European social survey 2014. Health Policy. 2019;123(8):747-55.

For peer review only - http://bmjopen.bmj.com/site/about/guidelines.xhtml

20. Hoebel J, Rommel A, Schröder SL, Fuchs J, Nowossadeck E, Lampert T. Socioeconomic Inequalities in Health and Perceived Unmet Needs for Healthcare among the Elderly in Germany. International journal of environmental research and public health. 2017;14(10).
21. Rotarou ES, Sakellariou D. Access to health care in an age of austerity: disabled people's unmet needs in Greece. Critical Public Health. 2019;29(1):48-60.
22. Sakellariou D, Rotarou ES. Access to healthcare for men and women with disabilities in the UK: secondary analysis of cross-sectional data. BMJ open. 2017;7(8):e016614.
23. OECD. OECD Health Statistics 2020. Paris: OECD Publishing; 2020.
24. OECD/European Observatory on Health Systems and Policies. State of Health in the EU Luxembourg Country Health Profile 2019. OECD Publishing, Paris/European Observatory on Health Systems and Policies, Brussels; 2019.
25. Berthet F, Calteux A, Wolter M, Weber L, van Ginneken E, Spranger A. Health Systems in Transition Luxembourg. Brussels: European Observatory on Health Systems and Policies; 2015.
26. CNS d'Gesondheetskees. Insuring family members 2020 [Available from: https://cns.public.lu/en/assure/vie-privee/coassurance.html.
27. CNS d'Gesondheetskees. Rapport annuel 2018. Luxembourg: CNS d'Gesondheetskees; 2019.
28. Statec. Le Portail des Statistiques Grand-Duché de Luxembourg Luxembourg: Statec; 2020 [Available from: https://statistiques.public.lu/stat/TableViewer/tableView.aspx?ReportId=12853&IF_Language=fra&MainTheme=2&FldrName=1.
29. Médecins du Monde. La santé pour tous. Rapport annuel 2018 Médecins du Monde Luxembourg Luxembourg: Médecins du Monde Luxembourg 2019.
30. Health Systems Characteristics Survey [Internet]. Available from: https://qdd.oecd.org/subject.aspx?Subject=hsc.
31. Goujard A. France: improving the efficiency of the healthcare system. Paris: OECD Publishing; 2018.
32. Institut national d'assurance maladie-invalidité. Tiers payant : ne payez que votre part, la mutualité paie directement le dispensateur de soins https://www.inami.fgov.be/fr/themes/cout-remboursement/facilite-financiere/Pages/tiers-payant.aspx2017 [
33. CNS d'Gesondheetskees. Reimbursement 2019 [Available from: https://cns.public.lu/en/assure/remboursement-prise-charge.html.
34. CNS d'Gesondheetskees. Reimbursed services 2019 [Available from: https://cns.public.lu/en/assure/vie-privee/sante-prevention.html.
35. CNS d'Gesondheetskees. Third-party social payment 2020 [Available from: https://cns.public.lu/en/professionnels-sante/medecins/tiers-payant-social.html.
36. Government of Luxembourg. Réunion du comité quadripartite en matière d'assurance maladie-maternité Communiqué 2019 [Available from: https://gouvernement.lu/fr/actualites/toutes_actualites/communiques/2019/11-novembre/13-reunion-quadripartite.html.
37. OECD. Waiting Times for Health Services Next in Line. Paris: OECD Publishing; 2020.
38. Santé.lu. European Health Interview Survey (EHIS) 2018 [Available from: https://sante.public.lu/fr/statistiques/ehis/index.html.
39. Luxembourg Ministry of Health Directorate of Health, Luxembourg Institute of Health. European Health Interview Survey (EHIS) Study Methodology. Luxembourg Luxembourg Ministry of Health Directorate of Health and Luxembourg Institute of Health; 2017.
40. Bejko D, Ruiz-Castell M, Schritz A, Laursen B, Kisser R, Rogmans W, et al. "To survey or to register" is that the question for estimating population incidence of injuries? Archives of Public Health. 2018;76(1):76.

For peer review only - http://bmjopen.bmj.com/site/about/guidelines.xhtml

41. Eurostat. Quality report of the second wave of the European Health Interview survey. Luxembourg: Publications Office of the European Union: European Union; 2018.
42. Ruiz-Castell M, Kandala NB, Perquin M, Bocquet V, Kuemmerle A, Vögele C, et al. Depression burden in luxembourg: Individual risk factors, geographic variations and the role of migration, 2013-2015 European Health Examination Survey. Journal of affective disorders. 2017;222:41-8.
43. Bataineh H, Devlin RA, Barham V. Unmet health care and health care utilization. Health economics. 2019;28(4):529-42.
44. StataCorp. Stata Statistical Software: Release 15. College Station, TX: StataCorp LLC.; 2017.
45. Eurostat. Self-reported unmet needs for health care by sex, age, specific reasons and educational attainment level Eurostat; 2020 [Available from: https://appsso.eurostat.ec.europa.eu/nui/show.do?dataset=hlth_ehis_un1e&lang=en.
46. Eurostat. Self-reported unmet needs for specific health care-related services due to financial reasons by sex, age and educational attainment level: Eurostat; 2020 [Available from: https://appsso.eurostat.ec.europa.eu/nui/show.do?dataset=hlth_ehis_un2e&lang=en.
47. OECD. Health at a Glance 2019. Paris: OECD Publishing; 2019.
48. Arora VS, Karanikolos M, Clair A, Reeves A, Stuckler D, McKee M. Data Resource Profile: The European Union Statistics on Income and Living Conditions (EU-SILC). International journal of epidemiology. 2015;44(2):451-61.
49. Iezzoni I. Risk adjustment for performance measurement. In: Smith PC, Mossialos E, Papanicolas I, Leatherman S, editors. Performance Measurement for Health System Improvement. Cambridge: Cambridge University Press; 2009.
50. Baeten R, Spasova S, Vanhercke B, Coster S. Inequalities in access to healthcare. A study of national policies. Brussels: European Commission; 2018.
51. Eurostat. Methodological issues in the analysis of the socioeconomic determinants of health using EU-SILC data. Luxembourg: Publications Office of the European Union; 2010.
52. OECD. Waiting Times for Health Services Next in Line. Paris: OECD Publishing; 2020.
53. Luxembourg Ministry of Health, Luxembourg Ministry of Social Security. Étude du fonctionnement des services d'urgence. Luxembourg: Luxembourg Ministry of Health and Luxembourg Ministry of Social Security; 2017.
54. Luxembourg Ministry of Health, Luxembourg Ministry of Social Security. Communiqué - Conférence de presse (15.12.2017) Vers une amélioration des services d'urgence Luxembourg: Luxembourg Ministry of Health and Luxembourg Ministry of Social Security; 2017 [Available from: https://sante.public.lu/fr/actualites/2017/12/urgences/urgences.pdf.
55. Department of Health and Social Care and Public Health England. Handbook to the NHS Constitution for England 2019 [Available from: https://www.gov.uk/government/publications/supplements-to-the-nhs-constitution-for-england/the-handbook-to-the-nhs-constitution-for-england#patients-and-the-public-your-rights-and-the-nhs-pledges-to-you.
56. CNS d'Gesondheetskees. Treatment abroad 2016 [Available from: https://cns.public.lu/en/assure/vie-privee/a-etranger.html.

For peer review only - http://bmjopen.bmj.com/site/about/guidelines.xhtml

Table 1 Prevalence of unmet need

Variable	N	%
Any unmet need (wait, distance or transportation, affordability)	3,521	35
Wait	1,639	32
Distance or transportation problems	1,531	4
Any affordability (medical, dental, prescribed medicines, mental health care)	2,078	15
Could not afford medical care	1,813	5
Could not afford dental care	1,888	12
Could not afford prescribed medicines	1,899	6
Could not afford mental health care	1,268	5

Note: N refers to number of respondents in estimation sample and % is percentage of estimation sample weighted for age, sex and district of residence.

For peer review only

For peer review only - http://bmjopen.bmj.com/site/about/guidelines.xhtml

Table 2. Characteristics of respondents reporting an unmet need due to wait, distance or affordability

	Wait (n=1,639)		Distance (n=1,531)		Could not afford medical care (n=1,813)		Could not afford dental care (n=1,888)		Could not afford prescribed medicines (n=1,899)		Could not afford mental health care (n=1,268)	
**Variable**	**N**	**(%)**	**N**	**(%)**	**N**	**(%)**	**N**	**(%)**	**N**	**(%)**	**N**	**(%)**
Sex												
Male	215	(45)	27	(57)	44	(51)	106	(52)	63	(61)	19	(35)
Female	314	(55)	24	(43)	50	(49)	116	(48)	45	(39)	44	(65)
Age												
15-24	35	(8)	10	(24)	6	(8)	15	(8)	7	(8)	4	(7)
25-34	119	(23)	11	(20)	16	(17)	46	(21)	21	(19)	22	(36)
35-44	119	(22)	10	(18)	20	(20)	46	(20)	12	(11)	15	(23)
45-54	119	(21)	3	(5)	25	(26)	55	(25)	31	(28)	10	(16)
55-64	78	(13)	6	(12)	14	(14)	37	(15)	16	(14)	11	(17)
65 and over	59	(12)	11	(21)	13	(15)	23	(11)	21	(20)	1	(1)
Marital status												
No	187	(37)	24	(50)	43	(46)	97	(45)	51	(50)	38	(62)
Yes	342	(63)	27	(50)	51	(54)	125	(55)	57	(50)	25	(38)
Immigrant												
No	322	(62)	29	(56)	60	(65)	134	(61)	80	(73)	31	(51)
Yes	207	(38)	22	(44)	34	(35)	88	(39)	28	(27)	32	(49)
Education												
Primary and pre-primary	34	(7)	5	(9)	11	(12)	18	(8)	12	(12)	5	(7)
Secondary & post-secondary	264	(50)	27	(54)	66	(71)	146	(66)	76	(71)	37	(61)
Tertiary	231	(43)	19	(37)	17	(17)	58	(25)	20	(18)	21	(32)
Job Status												
Employed	334	(62)	25	(47)	52	(54)	136	(61)	53	(47)	32	(50)

Unemployed	15	(3)	1	(2)	8	(9)	13	(6)	5	(5)	7	(12)
Retired	91	(17)	12	(22)	16	(16)	32	(14)	24	(21)	4	(6)
Student, domestic, compulsory service	74	(15)	10	(23)	11	(14)	30	(14)	19	(20)	13	(21)
Permanently disabled and other inactive status	15	(3)	3	(6)	7	(7)	11	(5)	7	(7)	7	(12)
Social support												
Low	19	(4)	4	(7)	8	(8)	16	(7)	10	(9)	10	(16)
Moderate	228	(44)	24	(48)	39	(42)	84	(39)	37	(35)	35	(55)
High	282	(52)	23	(45)	47	(50)	122	(54)	61	(56)	18	(29)
Household income												
Below 1st quintile (Lowest)	100	(19)	21	(40)	35	(37)	67	(30)	34	(32)	25	(40)
Between 1st & 2nd quintile	82	(16)	13	(30)	13	(15)	50	(23)	18	(17)	13	(20)
Between 2nd & 3rd quintile	133	(24)	6	(11)	24	(26)	57	(26)	33	(30)	15	(23)
Between 3rd & 4th quintile	108	(21)	7	(13)	12	(13)	29	(13)	12	(11)	7	(12)
Between 4th & 5th quintile (Highest)	106	(20)	4	(7)	10	(10)	19	(8)	11	(10)	3	(4)
Informal Carer												
No	419	(79)	43	(84)	75	(80)	183	(82)	88	(82)	53	(84)
Yes	110	(21)	8	(16)	19	(20)	39	(18)	20	(18)	10	(16)
BMI												
Normal or Underweight (<25)	286	(53)	23	(45)	47	(51)	93	(42)	42	(39)	28	(45)
Overweight (25-29)	141	(28)	14	(28)	19	(19)	78	(34)	33	(30)	19	(30)
Obese (>=30)	102	(19)	14	(27)	28	(30)	51	(23)	33	(31)	16	(25)
Smoker												
No	422	(80)	39	(76)	60	(65)	150	(67)	84	(78)	38	(60)
Occasionally	27	(5)	3	(7)	10	(10)	12	(5)	3	(2)	2	(3)
Daily	80	(15)	9	(18)	24	(24)	60	(28)	21	(20)	23	(37)
Alcohol consumption												
Never	102	(19)	13	(25)	22	(25)	55	(25)	23	(22)	14	(23)
Ex-drinkers	61	(11)	9	(18)	15	(16)	36	(17)	13	(12)	12	(20)
Irregularly	51	(9)	9	(15)	12	(12)	23	(10)	16	(15)	7	(10)

Regularly	180	(34)	10	(19)	27	(27)	52	(24)	34	(31)	19	(30)
Everyday	135	(26)	10	(23)	18	(20)	56	(25)	22	(20)	11	(16)
Self-assessed health												
Good/very good	325	(61)	17	(34)	40	(41)	113	(50)	56	(51)	29	(46)
Fair	142	(27)	20	(40)	26	(29)	74	(34)	31	(30)	18	(28)
Bad/Very bad	62	(12)	14	(26)	28	(30)	35	(16)	21	(19)	16	(26)
Chronic disease												
No	132	(25)	10	(20)	25	(26)	63	(28)	26	(32)	8	(13)
Yes	397	(75)	41	(80)	69	(74)	159	(72)	82	(68)	55	(87)
Limitations in activities due to health problems												
No limitations	272	(51)	17	(35)	36	(37)	106	(48)	44	(40)	24	(37)
Limited/severely limited	257	(49)	34	(65)	58	(63)	116	(52)	64	(60)	39	(63)
Canton												
Capellen	54	(10)	3	(6)	11	(12)	24	(10)	14	(12)	8	(12)
Clervaux	14	(3)	5	(9)	4	(5)	7	(4)	8	(8)	2	(3)
Diekirch	17	(3)	5	(11)	7	(8)	11	(6)	5	(5)	2	(3)
Echternach	14	(3)	2	(5)	4	(4)	9	(4)	3	(3)	2	(3)
Esch zur Alzette	143	(26)	12	(22)	30	(32)	70	(32)	29	(25)	18	(29)
Grevenmacher	37	(7)	5	(10)	6	(7)	15	(7)	9	(9)	4	(8)
Luxembourg	171	(32)	13	(26)	18	(18)	55	(23)	19	(17)	20	(31)
Mersch	37	(7)	1	(2)	5	(5)	10	(5)	9	(8)	1	(1)
Redange	16	(3)	1	(3)	3	(3)	4	(2)	2	(2)	1	(1)
Remich	17	(3)	2	(4)	3	(3)	9	(4)	5	(5)	3	(6)
Vianden	3	(1)	1	(2)	1	(1)	3	(1)	2	(2)	1	(2)
Wiltz	6	(1)	1	(2)	2	(3)	5	(2)	3	(3)	1	(1)

Note: N refers to number of respondents in estimation sample and (%) is percentage of estimation sample weighted for age, sex and district of residence.

For peer review only - http://bmjopen.bmj.com/site/about/guidelines.xhtml

Table 3. Multivariate logistic regression models for unmet need due to wait, distance or affordability

Variable	Wait (n=1,639)	Distance (n=1,531)	Could not afford medical care (n=1,813)	Could not afford dental care (n=1,888)	Could not afford prescribed medicines (n=1,899)	Could not afford mental health care (n=1,268)
Sex						
Male	1.00	1.00	1.00	1.00	1.00	1.00
Female	1.52 (1.19-1.93)**	0.83 (0.40-1.75)	1.04 (0.64-1.71)	0.96 (0.69-1.33)	0.55 (0.35-0.88)*	2.89 (1.41-5.94)**
Age						
15-24	1.00	1.00	1.00	1.00	1.00	1.00
25-34	1.32 (0.72-2.40)	0.27 (0.07-1.02)	1.76 (0.49-6.27)	1.62 (0.71-3.71)	3.22 (0.98-10.57)	6.39 (1.75-23.35)**
35-44	1.04 (0.55-1.93)	0.20 (0.05-0.75)*	1.87 (0.51-6.81)	1.36 (0.58-3.16)	1.53 (0.40-5.86)	2.49 (0.62-9.97)
45-54	1.00 (0.54-1.83)	0.04 (0.01-0.23)***	2.09 (0.58-7.49)	1.53 (0.66-3.56)	3.65 (1.05-12.66)*	1.33 (0.31-5.60)
55-64	0.59 (0.31-1.12)	0.14 (0.03-0.63)*	1.55 (0.39-6.06)	1.51 (0.62-3.68)	2.24 (0.58-8.58)	2.18 (0.51-9.35)
65 and over	0.44 (0.21-0.90)*	0.27 (0.07-1.07)	2.28 (0.50-10.36)	1.21 (0.46-3.19)	2.98 (0.76-11.75)	0.17 (0.01-2.16)
Marital status						
No	1.00	1.00	1.00	1.00	1.00	1.00
Yes	1.16 (0.89-1.52)	1.14 (0.47-2.78)	0.73 (0.43-1.23)	0.76 (0.53-1.08)	0.53 (0.33-0.84)**	0.40 (0.21-0.79)**
Immigrant						
No	1.00	1.00	1.00	1.00	1.00	1.00
Yes	0.79 (0.61-1.01)	1.18 (0.55-2.52)	0.79 (0.49-1.26)	0.85 (0.61-1.19)	0.58 (0.35-0.97)*	1.37 (0.68-2.76)
Education						
Primary and pre-primary	1.00	1.00	1.00	1.00	1.00	1.00
Secondary & post-secondary	1.14 (0.71-1.85)	1.26 (0.42-3.79)	1.48 (0.61-3.55)	1.6 (0.85-3.02)	1.35 (0.64-2.85)	1.55 (0.45-5.35)
Tertiary	1.40 (0.82-2.37)	2.59 (0.80-8.41)	0.55 (0.20-1.53)	1.2 (0.59-2.44)	0.67 (0.27-1.65)	1.22 (0.29-5.11)
Job Status						
Employed	1.00	1.00	1.00	1.00	1.00	1.00
Unemployed	0.62 (0.31-1.25)	0.21 (0.04-1.11)	1.74 (0.59-5.11)	1.4 (0.65-3.02)	1.51 (0.48-4.75)	1.51 (0.45-5.09)
Retired	1.22 (0.76-1.95)	0.91 (0.29-2.83)	0.6 (0.23-1.54)	0.58 (0.32-1.05)	0.88 (0.38-2.02)	1.2 (0.30-4.71)

For peer review only - http://bmjopen.bmj.com/site/about/guidelines.xhtml

Student, domestic, compulsory service	1.25 (0.82-1.91)	0.66 (0.23-1.85)	0.81 (0.33-2.01)	1.01 (0.58-1.76)	2.22 (1.04-4.76)*	2.95 (1.28-6.77)*
Permanently disabled and other inactive status	0.80 (0.38-1.69)	1.6 (0.29-8.81)	0.92 (0.35-2.41)	0.9 (0.44-1.84)	1.4 (0.58-3.37)	2.97 (0.92-9.64)
Social support						
Low	1.00	1.00	1.00	1.00	1.00	1.00
Moderate	1.54 (0.81-2.92)	0.84 (0.26-2.72)	0.6 (0.22-1.66)	0.46 (0.23-0.95)*	0.32 (0.14-0.77)*	0.27 (0.09-0.76)*
High	0.95 (0.50-1.81)	0.64 (0.19-2.22)	0.44 (0.16-1.23)	0.42 (0.21-0.86)*	0.30 (0.13-0.70)**	0.11 (0.03-0.35)***
Household income						
Below 1st quintile (Lowest)	1.00	1.00	1.00	1.00	1.00	1.00
Between 1st & 2nd quintile	0.85 (0.57-1.26)	0.74 (0.31-1.76)	0.37 (0.18-0.78)**	0.77 (0.48-1.21)	0.49 (0.26-0.95)*	0.49 (0.22-1.12)
Between 2nd & 3rd quintile	1.01 (0.70-1.48)	0.19 (0.06-0.54)**	0.73 (0.40-1.36)	0.77 (0.49-1.21)	0.77 (0.43-1.36)	0.78 (0.35-1.72)
Between 3rd & 4th quintile	1.16 (0.77-1.73)	0.35 (0.11-1.10)	0.47 (0.22-1.01)	0.46 (0.26-0.79)**	0.33 (0.15-0.70)**	0.47 (0.17-1.29)
Between 4th & 5th quintile (Highest)	0.91 (0.59-1.38)	0.14 (0.04-0.57)**	0.46 (0.20-1.07)	0.29 (0.16-0.53)***	0.38 (0.17-0.82)*	0.17 (0.05-0.61)**
Informal Carer						
No	1.00	1.00	1.00	1.00	1.00	1.00
Yes	1.36 (1.02-1.82)*	0.92 (0.42-2.03)	1.17 (0.67-2.03)	0.96 (0.63-1.45)	0.91 (0.53-1.56)	0.72 (0.32-1.64)
BMI						
Underweight or Normal	1.00	1.00	1.00	1.00	1.00	1.00
Overweight	0.81 (0.62-1.06)	1.04 (0.49-2.22)	0.49 (0.27-0.90)*	1.28 (0.90-1.83)	1.17 (0.69-1.99)	1.92 (0.94-3.91)
Obese	0.99 (0.72-1.35)	1.24 (0.61-2.55)	1.09 (0.62-1.92)	1.35 (0.89-2.02)	1.72 (0.96-3.08)	1.49 (0.68-3.27)
Smoker (No)						
No	1.00	1.00	1.00	1.00	1.00	1.00
Occasionally	1.06 (0.64-1.74)	1.38 (0.43-4.43)	2.76 (1.27-5.99)*	0.96 (0.49-1.86)	0.39 (0.10-1.44)	0.35 (0.07-1.64)
Daily	0.91 (0.66-1.26)	0.87 (0.38-1.96)	1.25 (0.72-2.15)	1.82 (1.28-2.60)**	0.90 (0.54-1.50)	2.15 (1.10-4.17)*
Alcohol consumption						
Never	1.00	1.00	1.00	1.00	1.00	1.00
Ex-drinkers	1.49 (0.95-2.35)	1.09 (0.41-2.90)	1.33 (0.59-3.01)	1.6 (0.93-2.77)	1.1 (0.49-2.49)	1.92 (0.73-5.08)
Irregularly	1.06 (0.68-1.63)	1.68 (0.64-4.38)	1.22 (0.54-2.73)	0.89 (0.51-1.55)	1.19 (0.59-2.41)	1.27 (0.40-4.04)
Regularly	1.12 (0.81-1.55)	0.75 (0.29-1.94)	0.89 (0.48-1.65)	0.63 (0.41-0.97)*	0.97 (0.54-1.74)	1.36 (0.59-3.17)
Everyday	1.07 (0.75-1.53)	0.97 (0.37-2.53)	0.85 (0.41-1.74)	1.02 (0.65-1.59)	0.8 (0.41-1.56)	1.2 (0.49-2.98)

For peer review only - http://bmjopen.bmj.com/site/about/guidelines.xhtml

Self-assessed health						
Good/very good	1.00	1.00	1.00	1.00	1.00	1.00
Fair	1.21 (0.90-1.62)	2.45 (1.17-5.12)*	1.26 (0.66-2.43)	1.63 (1.10-2.40) *	0.9 (0.52-1.55)	1.01 (0.44-2.28)
Bad/Very bad	2.41 (1.53-3.80)***	7.12 (2.91-17.44)***	5.35 (2.39-11.95)***	3.26 (1.86-5.71)***	2.22 (1.04-4.71)*	3.58 (1.25-10.30)*
Chronic disease						
No	1.00	1.00	1.00	1.00	1.00	1.00
Yes	1.45 (1.11-1.91)**	1.42 (0.60-3.37)	0.69 (0.38-1.28)	0.83 (0.57-1.21)	0.84 (0.48-1.44)	2.33 (0.89-6.08)
Limitations in activities due to health problems						
No limitations	1.00	1.00	1.00	1.00	1.00	1.00
Limited/severely limited	1.33 (1.02-1.73)*	1.37 (0.67-2.81)	1.39 (0.74-2.64)	1.15 (0.79-1.67)	1.49 (0.86-2.58)	1.53 (0.64-3.66)
Canton						
Esch zur Alzette	1.00	1.00	1.00	1.00	1.00	1.00
Capellen	1.21 (0.80-1.82)	1.08 (0.25-4.57)	1.42 (0.64-3.13)	1.41 (0.83-2.40)	2.12 (1.04-4.30)*	1.63 (0.61-4.37)
Clervaux	0.90 (0.46-1.75)	3.31 (0.78-14.14)	1.08 (0.31-3.81)	0.76 (0.31-1.88)	2.06 (0.77-5.53)	0.53 (0.12-2.31)
Diekirch	0.92 (0.49-1.72)	3.54 (1.02-12.32)	2.27 (0.87-5.95)	1.31 (0.60-2.83)	1.88 (0.68-5.18)	0.67 (0.12-3.85)
Echternach	1.43 (0.69-2.97)	2.02 (0.36-11.36)	1.63 (0.40-6.67)	1.47 (0.68-3.17)	1.13 (0.29-4.37)	1.19 (0.14-10.34)
Grevenmacher	1.67 (1.01-2.78)	3.06 (1.02-9.15)	1.38 (0.49-3.90)	1.27 (0.65-2.49)	2.53 (1.08-5.96)*	1.28 (0.30-5.51)
Luxembourg	1.27 (0.94-1.72)	1.4 (0.59-3.30)	0.8 (0.42-1.50)	0.99 (0.65-1.50)	1.02 (0.54-1.94)	1.54 (0.73-3.25)
Mersch	1.67 (1.01-2.76)*	0.43 (0.05-3.74)	1.11 (0.39-3.22)	0.88 (0.40-1.94)	2.38 (1.00-5.67)	0.29 (0.04-2.23)
Redange	1.07 (0.56-2.04)	0.95 (0.09-10.02)	0.91 (0.27-3.04)	0.53 (0.17-1.60)	0.60 (0.13-2.80)	0.80 (0.08-7.68)
Remich	0.99 (0.52-1.90)	1.64 (0.32-8.40)	0.75 (0.23-2.40)	1.05 (0.50-2.20)	1.51 (0.58-3.97)	3.45 (0.94-12.74)
Vianden	0.59 (0.14-2.46)	6.92 (0.62-77.07)	0.8 (0.14-4.45)	1.04 (0.29-3.67)	2.13 (0.58-7.77)	1.64 (0.24-11.19)
Wiltz	0.39 (0.15-0.99)	0.45 (0.07-3.16)	1.79 (0.33-9.75)	0.95 (0.34-2.71)	2.35 (0.57-9.65)	0.40 (0.05-3.14)
Constant	0.15 (0.05-0.42)***	0.08 (0.01-0.67)*	0.10 (0.02-0.57)*	0.22 (0.06-0.78)*	0.14 (0.03-0.59)**	0.01 (0.00-0.11)***

Note: Estimates are weighted for age, sex and district of residence. ***p < 0.001.**p < 0.01.*p < 0.05. Data are adjusted odds ratios (95% confidence intervals)

For peer review only - http://bmjopen.bmj.com/site/about/guidelines.xhtml

Figure 1. Map of Luxembourg cantons

Source: https://d-maps.com/m/europa/luxembourg/luxembourg88.gif

For peer review only

For peer review only - http://bmjopen.bmj.com/site/about/guidelines.xhtml

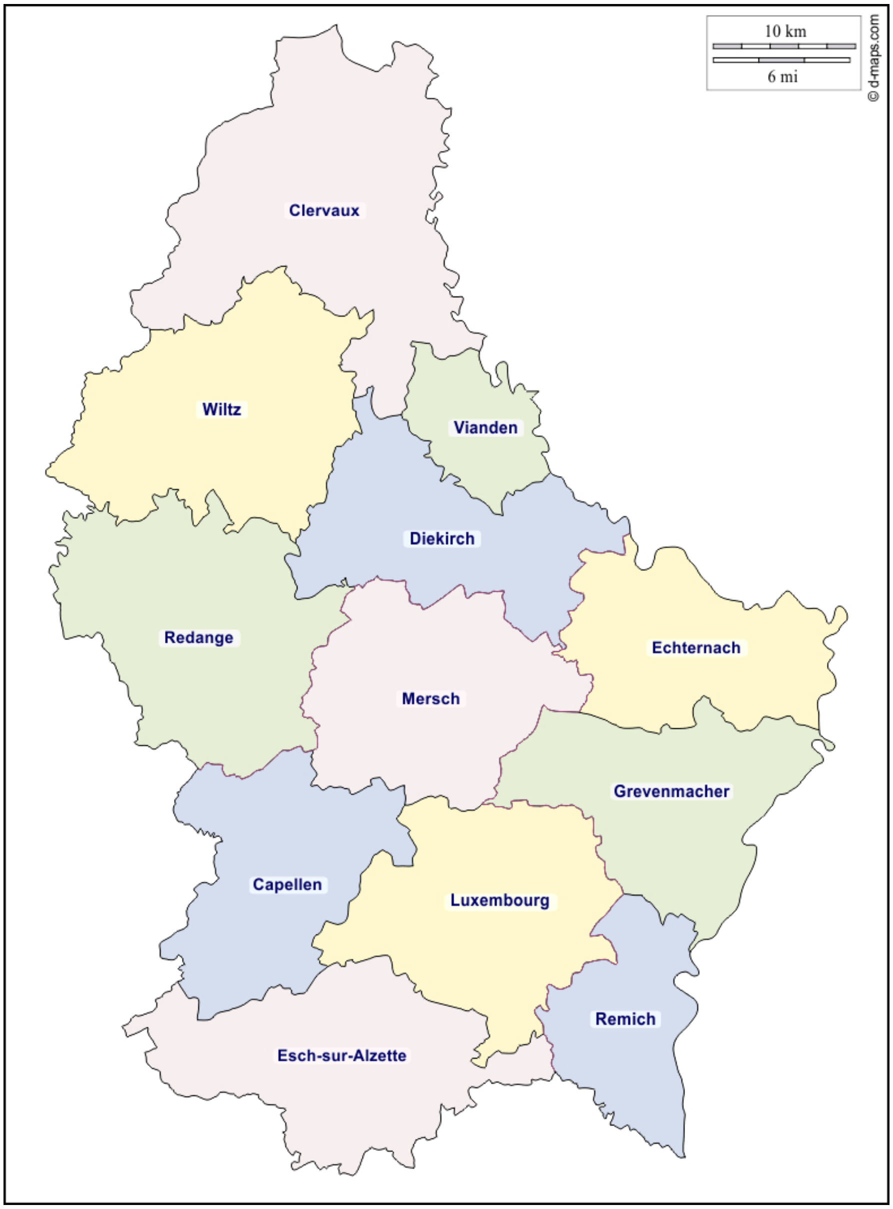

Figure 1. Map of Luxembourg cantons

276x373mm (72 x 72 DPI)

Appendix 1. Unmet Needs for Health Care Questions

3.22. In the **past 12 months**, have you experienced delay in getting health care because/due to…?

	Yes	No	No need for healthcare
The time needed to obtain an appointment was too long	☐	☐	☐
Distance or transportation problems	☐	☐	☐

3.23. Was there any time in the **past 12 months** you needed the following kinds of care, but could not afford it?

	Yes	No	No need for healthcare
Medical care	☐	☐	☐
Dental care	☐	☐	☐
Prescribed medicines	☐	☐	☐
Mental health care (by a psychologist or a psychiatrist for example)	☐	☐	☐

Source: Santé.lu. European Health Interview Survey (EHIS) 2018. Available from: https://sante.public.lu/fr/statistiques/ehis/ehis-methodologie/ehis-questionnaire-en.pdf

For peer review only

Appendix 2.

Table A1. Responses for unmet need measures

	Wait		Distance or transportation problems		Could not afford medical care		Could not afford dental care		Could not afford prescribed mediciness		Could not afford mental health care	
	N	%	N	%	N	%	N	%	N	%	N	%
No	1,906	48	2,482	62	2,866	72	2,809	70	2,977	74	2,097	52
Yes	878	22	92	2	182	5	399	10	216	5	106	3
No need for healthcare	1,043	26	983	25	782	20	599	15	609	15	1,533	38
Missing	177	4	447	11	174	4	197	5	202	5	268	7
**Total**	**4,004**	**100**	**4,004**	**100**	**4,004**	**100**	**4,004**	**100**	**4,004**	**100**	**4,004**	**100**

For peer review only

Table A2. Missing data for independent variables

Variable	N	%
Sex	0	0
Age	0	0
Marital status	123	3.34
Immigrant	27	0.71
Education	182	4.82
Employment status	284	7.30
Household income	1,116	28.84
Social support	66	1.69
Informal carer	170	4.46
BMI	58	1.43
Smoker	44	1.11
Alcohol consumption	143	3.64
Self-assessed health	51	1.28
Chronic disease	70	1.75
Limitations in activities due to health problems	217	5.60
Canton	6	0.14

Note: N refers to number of respondents in estimation sample and % is percentage of estimation sample weighted for age, sex and district of residence.

For peer review only

Table A3. Characteristics of respondents with and without missing income data

	Missing income				
	No		Yes		
**Variable**	**N**	**(%)**	**N**	**(%)**	**p-value**
Sex					
Male	1,415	(77)	425	(23)	<0.001
Female	1,473	(68)	691	(32)	
Age					
15-24	221	(47)	247	(53)	<0.001
25-34	526	(77)	154	(23)	
35-44	588	(80)	151	(20)	
45-54	599	(74)	206	(26)	
55-64	480	(74)	172	(26)	
65 and over	474	(72)	186	(28)	
Marital status					
No	1,061	(69)	474	(31)	<0.001
Yes	1,763	(75)	583	(25)	
Immigrant					
No	1,757	(70)	751	(30)	<0.001
Yes	1,113	(76)	356	(24)	
Education					
Primary and pre-primary	268	(67)	133	(33)	<0.001
Secondary & post-secondary	1,514	(72)	581	(28)	
Tertiary	1,020	(77)	306	(23)	
Job Status					
Employed	1,619	(79)	429	(21)	<0.001
Unemployed	75	(75)	25	(25)	
Retired	616	(76)	194	(24)	
Student, domestic, compulsory service	345	(53)	301	(47)	
Permanently disabled and other inactive status	80	(69)	36	(31)	
Social support					
Low	108	(73)	39	(27)	0.95
Moderate	1,030	(73)	389	(27)	
High	1,716	(72)	656	(28)	
Informal Carer					
No	2,295	(73)	843	(27)	0.354
Yes	497	(71)	199	(29)	
BMI					
Normal or Underweight (<25)	1,407	(70)	603	(30)	0.002
Overweight (25-29)	904	(76)	290	(24)	
Obese (>=30)	530	(71)	212	(29)	
Smoker					
No	2,244	(71)	908	(29)	0.007
Occasionally	165	(72)	63	(28)	

Daily	450	(78)	130	(22)	
Alcohol consumption					
Never	622	(74)	220	(26)	0.054
Ex-drinkers	225	(67)	112	(33)	
Irregularly	301	(75)	98	(25)	
Regularly	948	(72)	370	(28)	
Everyday	714	(74)	251	(26)	
Self-assessed health					
Good/very good	1,952	(71)	784	(29)	0.065
Fair	721	(75)	242	(25)	
Bad/Very bad	191	(75)	63	(25)	
Chronic disease					
No	937	(71)	390	(29)	0.066
Yes	1,913	(73)	694	(27)	
Limitations in activities due to health problems					
No limitations	1,711	(72)	682	(29)	0.005
Limited/severely limited	1,055	(76)	339	(24)	
Canton					
Capellen	263	(69)	117	(31)	0.252
Clervaux	94	(72)	36	(28)	
Diekirch	123	(72)	49	(28)	
Echternach	72	(71)	29	(29)	
Esch zur Alzette	854	(73)	309	(27)	
Grevenmacher	170	(76)	54	(24)	
Luxembourg	835	(73)	316	(27)	
Mersch	165	(68)	76	(32)	
Redange	92	(63)	53	(37)	
Remich	113	(72)	45	(28)	
Vianden	23	(82)	5	(18)	
Wiltz	80	(76)	25	(24)	

Note: N refers to number of respondents and (%) is percentage of estimation sample weighted for age, sex and district of residence.

only

Table A4. Sensitivity analysis with dummy variable for non-reporting of income

Variable	Wait (n=2,141)	Distance (n=2,000)	Could not afford medical care (n=2,348)	Could not afford dental care (n=2,465)	Could not afford prescribed mediciness (n=2,462)	Could not afford mental health care (n=1,672)
Sex						
Male	1.00	1.00	1.00	1.00	1.00	1.00
Female	1.55 (1.25-1.92)***	0.81 (0.44-1.49)	0.80 (0.52-1.23)	0.94 (0.7-1.25)	0.54 (0.36-0.80)**	2.34 (1.29-4.25)**
Age						
15-24	1.00	1.00	1.00	1.00	1.00	1.00
25-34	1.13 (0.7-1.8)	0.29 (0.1-0.84)*	2.78 (0.99-7.82)	1.48 (0.78-2.80)	2.46 (1.04-5.84)*	3.28 (1.21-8.90)*
35-44	0.84 (0.51-1.37)	0.16 (0.05-0.51)**	2.94 (1.00-8.66)	1.25 (0.64-2.46)	1.26 (0.46-3.48)	1.34 (0.47-3.80)
45-54	0.76 (0.47-1.23)	0.09 (0.03-0.28)***	3.65 (1.28-10.41)*	1.42 (0.73-2.77)	2.49 (1.01-6.15)*	0.87 (0.29-2.59)
55-64	0.48 (0.29-0.79)**	0.14 (0.04-0.49)**	2.54 (0.80-8.12)	1.2 (0.58-2.45)	1.86 (0.68-5.12)	0.76 (0.24-2.45)
65 and over	0.37 (0.21-0.67)**	0.29 (0.09-0.95)*	2.16 (0.55-8.45)	0.87 (0.39-1.96)	2.14 (0.72-6.34)	0.17 (0.03-1.13)
Marital status						
No	1.00	1.00	1.00	1.00	1.00	1.00
Yes	1.13 (0.89-1.43)	1.13 (0.54-2.34)	0.64 (0.41-1.01)	0.77 (0.56-1.06)	0.56 (0.37-0.86)**	0.53 (0.29-0.96)*
Immigrant						
No	1.00	1.00	1.00	1.00	1.00	1.00
Yes	0.80 (0.64-1)	0.95 (0.5-1.82)	0.95 (0.63-1.44)	0.98 (0.72-1.33)	0.62 (0.40-0.96)*	1.41 (0.76-2.6)
Education						
Primary and pre-primary	1.00	1.00	1.00	1.00	1.00	1.00
Secondary & post-secondary (non-tertiary)	1.20 (0.8-1.82)	1.61 (0.58-4.46)	1.74 (0.81-3.72)	1.61 (0.94-2.78)	1.06 (0.59-1.91)	2.03 (0.65-6.38)
Tertiary	1.42 (0.9-2.24)	3.27 (1.04-10.23)*	0.93 (0.39-2.22)	1.21 (0.65-2.24)	0.59 (0.29-1.23)	1.74 (0.47-6.44)
Job Status						
Employed	1.00	1.00	1.00	1.00	1.00	1.00
Unemployed	0.95 (0.52-1.76)	0.40 (0.09-1.84)	2.04 (0.85-4.87)	1.65 (0.83-3.30)	1.68 (0.69-4.12)	2.84 (1.01-7.97)*
Retired	1.1 (0.72-1.66)	0.64 (0.23-1.79)	1.07 (0.47-2.47)	0.78 (0.45-1.36)	0.98 (0.47-2.05)	1.49 (0.35-6.34)
Student, domestic, compulsoryservice	1.11 (0.79-1.57)	0.54 (0.21-1.34)	1.10 (0.54-2.25)	1.11 (0.71-1.75)	1.82 (0.97-3.40)	2.50 (1.20-5.24)*
Permanently disabled and other inactive status	0.72 (0.38-1.39)	0.82 (0.18-3.73)	1.06 (0.44-2.59)	1.05 (0.55-2.02)	1.11 (0.50-2.48)	1.83 (0.61-5.46)
Social support (Low)						

Low	1.00	1.00	1.00	1.00	1.00	1.00
Moderate	1.17 (0.67-2.04)	0.59 (0.22-1.56)	0.48 (0.22-1.06)	0.43 (0.24-0.78)**	0.35 (0.17-0.72)**	0.19 (0.08-0.44)***
High	0.74 (0.43-1.3)	0.47 (0.18-1.26)	0.35 (0.16-0.77)**	0.40 (0.22-0.72)**	0.30 (0.15-0.60)**	0.07 (0.03-0.16)***
Household income						
Below 1st quintile (Lowest)	1.00	1.00	1.00	1.00	1.00	1.00
Between 1st & 2nd quintile	0.87 (0.59-1.28)	0.75 (0.32-1.75)	0.38 (0.18-0.78)**	0.79 (0.51-1.22)	0.50 (0.27-0.94)*	0.55 (0.23-1.29)
Between 2nd & 3rd quintile	1.05 (0.73-1.52)	0.20 (0.07-0.58)**	0.68 (0.37-1.24)	0.79 (0.51-1.23)	0.76 (0.44-1.32)	0.87 (0.39-1.96)
Between 3rd & 4th quintile	1.19 (0.81-1.76)	0.37 (0.12-1.12)	0.41 (0.19-0.87)*	0.48 (0.28-0.81)**	0.32 (0.16-0.67)**	0.52 (0.19-1.42)
Between 4th & 5th quintile (Highest)	0.97 (0.65-1.44)	0.16 (0.04-0.61)**	0.39 (0.17-0.87)*	0.30 (0.17-0.54)***	0.35 (0.17-0.75)**	0.20 (0.06-0.71)*
Income not reported	0.85 (0.60-1.21)	0.52 (0.24-1.14)	0.63 (0.36-1.12)	0.64 (0.42-0.97)*	0.69 (0.41-1.19)	0.80 (0.38-1.72)
Informal Carer						
No	1.00	1.00	1.00	1.00	1.00	1.00
Yes	1.36 (1.06-1.75)*	1.08 (0.55-2.09)	1.37 (0.87-2.15)	1.07 (0.75-1.52)	1.07 (0.69-1.66)	0.63 (0.31-1.28)
BMI						
Underweight or Normal	1.00	1.00	1.00	1.00	1.00	1.00
Overweight	0.83 (0.66-1.06)	0.84 (0.44-1.63)	0.53 (0.31-0.90)*	1.25 (0.91-1.72)	1.10 (0.69-1.74)	2.26 (1.20-4.24)*
Obese	0.98 (0.74-1.3)	1.41 (0.77-2.59)	0.98 (0.58-1.65)	1.32 (0.91-1.90)	1.57 (0.95-2.57)	1.61 (0.81-3.21)
Smoker (No)						
No	1.00	1.00	1.00	1.00	1.00	1.00
Occasionally	1.51 (0.99-2.29)	1.92 (0.85-4.31)	2.24 (1.13-4.45)*	0.80 (0.43-1.48)	0.31 (0.09-1.06)	0.27 (0.06-1.24)
Daily	0.95 (0.71-1.27)	0.91 (0.43-1.9)	1.31 (0.81-2.12)	1.72 (1.25-2.38)**	1.04 (0.66-1.64)	2.04 (1.13-3.69)*
Alcohol consumption						
Never	1.00	1.00	1.00	1.00	1.00	1.00
Ex-drinkers	1.30 (0.87-1.94)	0.80 (0.32-1.97)	1.57 (0.78-3.19)	1.09 (0.67-1.77)	1.19 (0.6-2.36)	1.77 (0.74-4.21)
Irregularly	1.05 (0.71-1.56)	1.11 (0.46-2.65)	1.18 (0.57-2.42)	0.81 (0.5-1.33)	1.27 (0.69-2.37)	1.43 (0.57-3.61)
Regularly	1.16 (0.87-1.55)	0.54 (0.24-1.19)	1.00 (0.59-1.68)	0.60 (0.42-0.87)**	0.86 (0.52-1.41)	1.42 (0.69-2.93)
Everyday	1.07 (0.79-1.45)	0.70 (0.3-1.63)	0.94 (0.51-1.74)	0.78 (0.53-1.17)	0.91 (0.53-1.57)	1.18 (0.52-2.67)
Self-assessed health						
Good/very good	1.00	1.00	1.00	1.00	1.00	1.00
Fair	1.10 (0.85-1.44)	3.5 (1.88-6.53)***	1.41 (0.83-2.4)	1.52 (1.07-2.15)*	0.88 (0.55-1.41)	1.36 (0.68-2.73)
Bad/Very bad	1.86 (1.25-2.79)**	7.88 (3.71-16.73)***	3.76 (1.89-7.48)***	2.66 (1.61-4.39)***	1.67 (0.85-3.29)	2.40 (0.99-5.84)
Chronic disease (No)						
No	1.00	1.00	1.00	1.00	1.00	1.00
Yes	1.45 (1.14-1.84)**	1.09 (0.53-2.22)	0.77 (0.46-1.29)	0.90 (0.65-1.26)	0.75 (0.48-1.18)	2.04 (0.96-4.35)

For peer review only - http://bmjopen.bmj.com/site/about/guidelines.xhtml

Limitations in activities due to health problems						
No limitations	1.00	1.00	1.00	1.00	1.00	1.00
Limited/severely limited	1.56 (1.23-1.97)***	1.30 (0.72-2.35)	1.24 (0.74-2.10)	1.11 (0.80-1.55)	1.56 (0.99-2.45)	2.17 (1.08-4.35)*
Canton						
Esch zur Alzette	1.00	1.00	1.00	1.00	1.00	1.00
Capellen	1.12 (0.78-1.60)	1.17 (0.34-4.01)	1.12 (0.54-2.33)	1.09 (0.67-1.78)	1.33 (0.7-2.52)	1.50 (0.62-3.68)
Clervaux	0.81 (0.45-1.46)	3.63 (1.14-11.52)*	0.96 (0.31-2.92)	0.64 (0.27-1.48)	1.40 (0.56-3.49)	0.29 (0.05-1.68)
Diekirch	0.94 (0.54-1.62)	3.92 (1.27-12.04)*	2.04 (0.89-4.67)	1.17 (0.59-2.33)	2.06 (0.91-4.68)	1.58 (0.41-6.13)
Echternach	1.22 (0.64-2.31)	2.63 (0.64-10.84)	1.81 (0.59-5.60)	1.21 (0.59-2.46)	1.20 (0.38-3.81)	0.64 (0.06-6.66)
Grevenmacher	1.38 (0.89-2.16)	2.27 (0.77-6.66)	1.07 (0.42-2.72)	1.47 (0.83-2.59)	2.12 (1.03-4.35)*	1.53 (0.48-4.86)
Luxembourg	1.17 (0.90-1.53)	1.48 (0.68-3.23)	0.77 (0.46-1.31)	0.94 (0.66-1.35)	1.01 (0.60-1.68)	1.60 (0.83-3.10)
Mersch	1.75 (1.13-2.72)*	0.88 (0.18-4.39)	1.65 (0.71-3.85)	1.00 (0.51-1.92)	2.04 (1.00-4.16)	0.51 (0.10-2.54)
Redange	0.96 (0.56-1.63)	1.37 (0.25-7.53)	1.31 (0.53-3.28)	0.53 (0.22-1.30)	0.60 (0.18-2.03)	0.67 (0.13-3.34)
Remich	1.02 (0.57-1.84)	3.70 (1.1-12.5)*	0.66 (0.21-2.06)	0.85 (0.41-1.73)	1.30 (0.53-3.15)	3.64 (1.07-12.36)*
Vianden	0.49 (0.12-1.90)	6.99 (0.65-75.66)	0.69 (0.12-3.86)	0.91 (0.27-3.06)	2.37 (0.77-7.31)	1.69 (0.28-10.20)
Wiltz	0.57 (0.27-1.21)	1.44 (0.28-7.35)	1.74 (0.44-6.81)	0.98 (0.42-2.27)	1.43 (0.38-5.37)	0.93 (0.16-5.38)
Constant	0.22 (0.09-0.51)**	0.12 (0.02-0.73)*	0.07 (0.01-0.30)***	0.28 (0.10-0.80)*	0.25 (0.08-0.84)*	0.02 (0.00-0.11)***

Note: Estimates are weighted for age, sex and district of residence. ***p < 0.001.**p < 0.01.*p < 0.05.

For peer review only - http://bmjopen.bmj.com/site/about/guidelines.xhtml

STROBE Statement—Checklist of items that should be included in reports of ***cross-sectional studies***

	Item No	Recommendation	Page No
**Title and abstract**	1	(*a*) Indicate the study's design with a commonly used term in the title or the abstract	1
		(*b*) Provide in the abstract an informative and balanced summary of what was done and what was found	2
**Introduction**			
Background/rationale	2	Explain the scientific background and rationale for the investigation being reported	4
Objectives	3	State specific objectives, including any prespecified hypotheses	4
**Methods**			
Study design	4	Present key elements of study design early in the paper	6
Setting	5	Describe the setting, locations, and relevant dates, including periods of recruitment, exposure, follow-up, and data collection	6
Participants	6	(*a*) Give the eligibility criteria, and the sources and methods of selection of participants	6
Variables	7	Clearly define all outcomes, exposures, predictors, potential confounders, and effect modifiers. Give diagnostic criteria, if applicable	6 and 7
Data sources/ measurement	8*	For each variable of interest, give sources of data and details of methods of assessment (measurement). Describe comparability of assessment methods if there is more than one group	6 and 7
Bias	9	Describe any efforts to address potential sources of bias	7
Study size	10	Explain how the study size was arrived at	6
Quantitative variables	11	Explain how quantitative variables were handled in the analyses. If applicable, describe which groupings were chosen and why	6 and 7
Statistical methods	12	(*a*) Describe all statistical methods, including those used to control for confounding	7
		(*b*) Describe any methods used to examine subgroups and interactions	NA
		(*c*) Explain how missing data were addressed	7
		(*d*) If applicable, describe analytical methods taking account of sampling strategy	7
		(*e*) Describe any sensitivity analyses	7
**Results**			
Participants	13*	(a) Report numbers of individuals at each stage of study—eg numbers potentially eligible, examined for eligibility, confirmed eligible, included in the study, completing follow-up, and analysed	6 and 7
		(b) Give reasons for non-participation at each stage	6 and 7
		(c) Consider use of a flow diagram	
Descriptive data	14*	(a) Give characteristics of study participants (eg demographic, clinical, social) and information on exposures and potential confounders	8, 19-21
		(b) Indicate number of participants with missing data for each variable of interest	Tables A1 and A2
Outcome data	15*	Report numbers of outcome events or summary measures	8 and 18

For peer review only

Main results	16	(*a*) Give unadjusted estimates and, if applicable, confounder-adjusted estimates and their precision (eg, 95% confidence interval). Make clear which confounders were adjusted for and why they were included	8-9, 22-24
		(*b*) Report category boundaries when continuous variables were categorized	6-7
		(*c*) If relevant, consider translating estimates of relative risk into absolute risk for a meaningful time period	N/A
Other analyses	17	Report other analyses done—eg analyses of subgroups and interactions, and sensitivity analyses	7 and Table A4
**Discussion**			
Key results	18	Summarise key results with reference to study objectives	9
Limitations	19	Discuss limitations of the study, taking into account sources of potential bias or imprecision. Discuss both direction and magnitude of any potential bias	10
Interpretation	20	Give a cautious overall interpretation of results considering objectives, limitations, multiplicity of analyses, results from similar studies, and other relevant evidence	10-12
Generalisability	21	Discuss the generalisability (external validity) of the study results	9-10
**Other information**			
Funding	22	Give the source of funding and the role of the funders for the present study and, if applicable, for the original study on which the present article is based	14

*Give information separately for exposed and unexposed groups.

Note: An Explanation and Elaboration article discusses each checklist item and gives methodological background and published examples of transparent reporting. The STROBE checklist is best used in conjunction with this article (freely available on the Web sites of PLoS Medicine at http://www.plosmedicine.org/, Annals of Internal Medicine at http://www.annals.org/, and Epidemiology at http://www.epidem.com/). Information on the STROBE Initiative is available at www.strobe-statement.org.

For peer review only

BMJ Open

Investigating unmet need for health care using the European Health Interview Survey: a cross-sectional survey study of Luxembourg

Journal:	*BMJ Open*
Manuscript ID	bmjopen-2021-048860.R1
Article Type:	Original research
Date Submitted by the Author:	12-May-2021
Complete List of Authors:	Moran, Valerie; Luxembourg Institute of Health; Luxembourg Institute of Socio-Economic Research,<br>Suhrcke, Marc; Luxembourg Institute of Socio-Economic Research; University of York Centre for Health Economics<br>Ruiz-Castell, Maria; Luxembourg Institute of Health,<br>Barré, Jessica; Caisse nationale de santé<br>Huiart, Laetitia; Santé publique France
**Primary Subject Heading**:	Health policy
Secondary Subject Heading:	Health services research, Public health
Keywords:	Health economics < HEALTH SERVICES ADMINISTRATION & MANAGEMENT, Health policy < HEALTH SERVICES ADMINISTRATION & MANAGEMENT, International health services < HEALTH SERVICES ADMINISTRATION & MANAGEMENT, Organisation of health services < HEALTH SERVICES ADMINISTRATION & MANAGEMENT

SCHOLARONE™
Manuscripts

I, the Submitting Author has the right to grant and does grant on behalf of all authors of the Work (as defined in the below author licence), an exclusive licence and/or a non-exclusive licence for contributions from authors who are: i) UK Crown employees; ii) where BMJ has agreed a CC-BY licence shall apply, and/or iii) in accordance with the terms applicable for US Federal Government officers or employees acting as part of their official duties; on a worldwide, perpetual, irrevocable, royalty-free basis to BMJ Publishing Group Ltd ("BMJ") its licensees and where the relevant Journal is co-owned by BMJ to the co-owners of the Journal, to publish the Work in this journal and any other BMJ products and to exploit all rights, as set out in our licence.

The Submitting Author accepts and understands that any supply made under these terms is made by BMJ to the Submitting Author unless you are acting as an employee on behalf of your employer or a postgraduate student of an affiliated institution which is paying any applicable article publishing charge ("APC") for Open Access articles. Where the Submitting Author wishes to make the Work available on an Open Access basis (and intends to pay the relevant APC), the terms of reuse of such Open Access shall be governed by a Creative Commons licence – details of these licences and which Creative Commons licence will apply to this Work are set out in our licence referred to above.

Other than as permitted in any relevant BMJ Author's Self Archiving Policies, I confirm this Work has not been accepted for publication elsewhere, is not being considered for publication elsewhere and does not duplicate material already published. I confirm all authors consent to publication of this Work and authorise the granting of this licence.

For peer review only

Investigating unmet need for health care using the European Health Interview Survey: a cross-sectional survey study of Luxembourg

Valerie Moran*[1,2], Marc Suhrcke[2,3], Maria Ruiz-Castell[1], Jessica Barré[4], Laetitia Huiart[5].

[1] Luxembourg Institute of Health (LIH), Strassen, Luxembourg
[2] Luxembourg Institute of Socio-Economic Research (LISER), Esch-sur-Alzette, Luxembourg
[3] Centre for Health Economics, University of York, York, UK
[4] Caisse national de santé, Luxembourg, Luxembourg
[5] Santé Publique, Paris, France

*Corresponding author:
Valerie Moran
Valerie.Moran@lih.lu, valerie.moran@liser.lu

For peer review only - http://bmjopen.bmj.com/site/about/guidelines.xhtml

Abstract

Objectives: We investigate the prevalence of unmet need arising from wait times, distance/transportation and financial affordability using the European Health Interview Survey. We explore associations between individual characteristics and the probability of reporting unmet need.

Design: Cross-sectional survey conducted between February and December 2014.

Setting and participants: 4,004 members of the resident population in private households registered with the health insurance fund in Luxembourg aged 15 years and over.

Outcome measures: Six binary variables that measured unmet need arising from wait time, distance/transportation and affordability of medical, dental and mental health care and prescribed medicines among those who reported a need for care.

Results: The most common barrier to access arose from wait times (32%) and the least common from distance/transportation (4%). Dental care (12%) was most often reported as unaffordable, followed by prescribed medicines (6%), medical (5%) and mental health (5%) care. Respondents who reported bad/very bad health were associated with a higher risk of unmet need compared to those with good/very good health (wait: OR 2.41, 95% CI 1.53-3.80, distance/transportation: OR 7.12 95% CI 2.91-17.44, afford medical care: OR 5.35 95% CI 2.39-11.95, afford dental care: OR 3.26 95% CI 1.86-5.71, afford prescribed medicines: OR 2.22 95% CI 1.04-4.71, afford mental health care: OR 3.58 95% CI 1.25-10.30). Income between the fourth and fifth quintiles was associated with a lower risk of unmet need for dental care (OR 0.29, 95% CI 0.16-0.53), prescribed medicines (OR 0.38, 95% CI 0.17-0.82) and mental health care (OR 0.17, 95% CI 0.05-0.61) compared to income between the first and second quintiles.

Conclusions: Recent and planned reforms to address waiting times and financial barriers to accessing health care may help to address unmet need. In addition, policymakers should consider additional policies targeted at high-risk groups with poor health and low incomes.

For peer review only

Strengths and limitations of this study

We investigated unmet need using a representative cross-sectional population based survey – the European Health Interview Survey (EHIS).

The EHIS allowed us to investigate unmet need due to the affordability of prescribed medications and mental health care, in addition to medical and dental care.

As unmet need was self-reported, it may be subject to recall bias or response bias.

The EHIS does not collect data on unmet need due to wait for different types of services.

This study explored associations between unmet need and socio-demographic, health status and risk factor variables but did not establish causality.

For peer review only

Introduction

In recent years, the concept of universal health coverage (UHC) has emerged as a key objective of international organisations including the WHO, OECD and World Bank, who support national governments to achieve this goal. UHC is defined as “ensuring that all people have access to needed health services…..of sufficient quality to be effective while also ensuring that the use of these services does not expose the user to financial hardship” [1]. Therefore, access to care is a core component of UHC. At the EU level, the European Parliament, Council and Commission announced the European Pillar of Social Rights in 2017 [2]. The Pillar comprises 20 guiding principles that underpin a fair and inclusive society. Chapter 3 of the Pillar relates to social protection and inclusion and contains the principle that everyone is entitled to timely access to good quality affordable health care [3]. Barriers to accessing health care arising from the cost, physical accessibility and quality of services can lead to unmet need at an individual level due to affordability, distance and waiting times [4, 5]. Unmet need may also arise from other factors related to personal choices and circumstances including a lack of time to seek care due to family or work responsibilities, fear or dislike of medical personnel and treatment, a preference to wait and see if the symptoms resolve by themselves without seeking care and issues related to health literacy including language problems and a lack of knowledge of appropriate medical care [6, 7].

view only

Countries have introduced various policies to address different aspects of unmet need. Many countries have implemented maximum waiting times, particularly for specialist consultations and elective treatments [8]. Countries have also introduced policies to reduce financial barriers to accessing health care. Belgium has put in place a range of financial protection measures including lower co-payments for vulnerable groups and ceilings on the total amount of co-payments paid by a household based on household income, regulation of supplementary payments and the third party payment system (outlined in more detail below) [9]. In France, individuals with chronic illnesses, pregnant women, low-income groups and individuals who suffered a work accident are exempt from most or all co-payments [10, 11].

Previous literature on unmet need for health care

Survey data is commonly used to ascertain individuals’ perceptions of unmet need arising from various barriers to accessing care [12, 13]. To date, studies that investigated unmet need within and across European countries have used data from the European Union Statistics on Income and Living Conditions (EU-SILC) [14-25], the European Social Survey (ESS) [26, 27] and the European Health Interview Survey (EHIS) [28-30]. Several studies that investigated unmet

For peer review only - http://bmjopen.bmj.com/site/about/guidelines.xhtml

need for medical care [14, 16, 18, 19, 21, 27] found that females were associated with higher unmet need compared to males. While there were conflicting results across studies for variables measuring age, education, employment, immigrant status and urban vs rural area, there was consensus for other covariates. Respondents in poorer health [14, 16, 23, 25-27] and those with a chronic condition or illness [14, 16, 25, 27] were more likely to report unmet need. Higher income groups were less likely to report unmet need [14, 16, 23, 25, 27] as were respondents with greater social capital and social support [19, 27]. A small number of studies examined the determinants of unmet need for different services. Chaupain-Guillot and Guillot [15] found that older age was associated with a lower probability of reporting unmet need for medical and dental care while poorer health status and lower income was associated with a higher probability. However, while a higher level of education was associated with an increased probability of reporting unmet need for medical care, it was associated with a reduced probability of reporting unmet need for dental care. Hoebel et al. [28] investigated unmet need among older people with low socio-economic status in Germany. Among those aged 50-64, low socio-economic status was associated with higher unmet need for medical, dental and mental health care for both men and women. However, among the 65-85 age group low socio-economic status was associated with higher unmet need only among men for medical and dental care. Rotarou et al. and Sakellariou et al. [29, 30] found that people with a disability were more likely to report higher unmet need for medical, dental and mental health care in Greece and the UK respectively.

The Luxembourg health system and access to health care

Luxembourg provides universal coverage of health care through a mandatory social health insurance system, the *Caisse Nationale de Santé* (CNS). In 2018, the CNS covered 93% of the resident population [31, 32]. The proportion not covered included EU officials based in Luxembourg, who were insured under a separate scheme provided by their employer [33] and vulnerable populations including the homeless and irregular immigrants and their families [34].

In Luxembourg, when patients access health care including doctor consultations in outpatient or inpatient settings, dental and paramedical services, they must pay providers the full cost upon receipt of care and then apply for a refund from the CNS for the covered share of the payment (excluding co-payments). The CNS directly reimburses providers for hospital services (excluding the doctors' fees), laboratory tests and pharmaceuticals, leaving the patient to pay only the co-payment at the point of use [35].

For peer review only - http://bmjopen.bmj.com/site/about/guidelines.xhtml

Luxembourg is among a minority of countries (including Belgium and France) in the EU where patients pay ambulatory care providers directly and then claim reimbursement from the social health insurance fund [36]. The payment of the full cost of medical, dental and mental health care by the patient upon receipt of care may create a financial barrier to accessing care for certain groups, for example, those on lower incomes [34]. In Belgium and France, specific population groups including those on low incomes or with a chronic illness have their costs directly covered by the social insurance fund, a scheme known as the '*tiers payant'* or third party payment system [10, 37]. In Luxembourg, the government introduced the system of 'third-party social payment' ('*tiers payant social*') in 2013, which entitles people in economic hardship to request assistance with the payment of health care expenses [38]. Eligible patients are exempted from the payment of costs and the CNS reimburses the provider. The local social welfare office covers any co-payments the patient cannot afford. The purpose of this policy is to enable people in economic hardship to access health care [38]. Therefore, the third-party payment system addresses unmet need arising from financial barriers to care in order to reduce financial hardship arising from health care utilisation [9]. However, it is unclear what the impact of this policy has been on addressing unmet need due to affordability of care in Luxembourg, as the policy has not been evaluated to date. Nevertheless, following a national debate over recent years, the government announced in November 2019 that a universal system of third-party payment would be introduced [39], which would cover the entire enrolled population of the CNS and replace the existing third-party social payment.

In addition to financial access to care, waiting times for health services have been identified as an important policy issue in Luxembourg. Waiting times are commonly a pertinent issue in countries with a national health system funded by general taxation. However, a recent report highlighted that waiting times were a medium to high priority in Luxembourg, in contrast to its neighbouring countries of Belgium and Germany, where waiting times were a low to medium or low priority [8]. This report also revealed that waiting times were an issue across different types of services including specialist care, diagnostic tests, hospital emergency departments, primary care and cancer care. Data on waiting times for health care in Luxembourg are not routinely published. However, a 2016 study revealed an average wait of almost four hours between admission to and discharge from emergency services, which are provided by each of the four general hospitals. The majority (75%) of attendees experienced a wait of three hours or less, just below the government target of 85% [40]. These findings prompted the government to set a maximum waiting time target of 2.5 hours for emergency services [41]. Efforts have also been undertaken to improve the organisation of cancer care in order to reduce waiting times. In 2016, the government embarked on a reform of the National Health Laboratory's diagnostic services by reducing outsourcing to other countries and concentrating the delivery

For peer review only - http://bmjopen.bmj.com/site/about/guidelines.xhtml

of these services in Luxembourgish hospitals instead. The government also introduced maximum waiting time targets for cancer care. At least 95% of patients should receive a diagnosis within five working days while specific targets are in place for different types of cancer (e.g. a maximum of four weeks between chemotherapy and radiotherapy or two weeks following receipt of the analytical report) [8]. If residents of Luxembourg enrolled in the CNS perceive wait times as too long, they may seek health care in another EU state. Prior authorisation from the CNS is not required for a doctor consultation (in a health centre, clinic or hospital) but is required if the consultation uses specialised medical equipment or hospital infrastructure or for inpatient treatments with at least one overnight stay. The CNS may withhold authorisation if the necessary treatment can be provided in Luxembourg within a medically justifiable timeframe [42].

The issue of unmet need for health care in Luxembourg has not been studied to date despite its policy relevance and the availability of relevant survey data. The objective of this paper is to investigate the prevalence and determinants of unmet need in Luxembourg. We used EHIS data as it allowed us to explore the prevalence and determinants of unmet need separately for waiting time, distance or transportation and the affordability of medical, dental and mental health care and prescribed medicines. Therefore, we also contribute to the limited number of studies that investigated unmet need not only for medical care but also for dental and mental health care and prescribed medications.

Data and methods

Study population and design

The EHIS is a cross-sectional observational survey undertaken in all EU countries. A first wave of data was collected in 17 EU member states (excluding Luxembourg) between 2006 and 2009 [43]. A second wave of the survey (EHIS 2) was collected in all 28 EU countries together with Iceland, Norway and Turkey between 2013 and 2015. Detailed information on the EHIS 2 methodology is available in a manual published by Eurostat [43] while a paper published by the Robert Koch Institute in Germany [44] provides a concise overview of the background and study methodology of the EHIS 2. Information on the EHIS 2 for Luxembourg, including the questionnaire and data access procedure is available on the Ministry of Health Directorate of Health website [45]. The survey collected information on health status, health determinants, utilisation of and barriers to access to health care and sociodemographic characteristics [46]. The coverage of the survey included the resident population in private households aged 15 years and over. A one-stage random sample stratified by age, sex and district of residence (Luxembourg, Diekirch and Grevenmacher) was drawn from the registry of CNS insurees [45,

For peer review only - http://bmjopen.bmj.com/site/about/guidelines.xhtml

47]. Among the 16,000 individuals invited to participate, 4,823 responded (response rate of 30.1%) by submitting an electronic (70%) or paper (30%) questionnaire [46]. Of these respondents, 4,118 participants met the inclusion criteria, provided informed consent and completed the questionnaire (participation rate of 24.7%). Data were collected between February and December 2014 [47]. The EHIS 2 Luxembourg database comprised 4,004 individuals who completed more than 50% of the questionnaire and had no missing data for age, sex or district, (final participation rate of 25%). This database was prepared according to a European protocol [48] and was validated by Eurostat [45].

The EHIS differs from the EU-SILC and ESS as it does not ask respondents a binary (yes/no) question on whether they have unmet need. Rather, the EHIS asks respondents to consider unmet need arising from specific barriers to accessing health care, including long waits, distance or transportation problems and the affordability of services. The EHIS data allows the investigation of each component of unmet need separately and the consideration of financial barriers for medical, dental and mental health care and prescribed medicines. The survey questions are available in Appendix 1 of the online supplementary material. Table 1 shows the number and percentage of respondents who reported no unmet need, an unmet need or no need for health care for each component of unmet need. The percentage of respondents who reported no need for health care ranged from 15% for affordability of dental care and prescribed medicines to 38% for mental health care.

Insert Table 1 around here

Outcome variables

For our outcome variables, we created six binary variables to measure barriers to access due to wait time, distance and affordability of medical care, dental care, prescribed medicines and mental health care among those who reported a need for care. We coded these variables as one, if respondents replied "Yes", as zero if respondents replied "No" and as missing if respondents reported "No need for healthcare".

Explanatory variables

Explanatory variables covered socio-demographics, health behaviours and health status. Socio-demographic variables included sex, age, marital status, immigrant, education, employment status, income, social support and being an informal carer. Sex, marital status, immigrant and informal carer were constructed as binary variables while age, education and employment status were constructed as categorical variables. Household income was measured in quintiles and included as a categorical variable. Following Ruiz-Castell et al. (2017) [49], we created a categorical variable on low, moderate and high social support using

For peer review only - http://bmjopen.bmj.com/site/about/guidelines.xhtml

the questions: “how much concern do people show in what you are doing?” and “how easy is it to get practical help from neighbours if you should need it?”. Health risk factors included Body Mass Index (BMI), smoking and alcohol consumption. We considered alcohol consumption as ‘irregular’, if it occurred at most 2-3 days per month, and ‘regular’, if it occurred at least once per week. We used three measures of health status: self-assessed health, presence of a chronic disease and limitations in activities due to health problems. We also included binary variables (fixed effects) for the twelve cantons (see Figure 1) to capture geographical differences in unmet need.

Insert Figure 1 around here

We excluded observations with missing data for the explanatory variables. Table A1 in Appendix 2 of the online supplementary material shows the percentage of missing data for each explanatory variable. Missing data for the explanatory variables did not exceed 7%, except for income, where 29% of observations had missing data. Table A2 in Appendix 2 shows the characteristics of respondents with missing income data. These were more likely female, aged 15-24, unmarried, of Luxembourgish nationality, had only primary and pre-primary education, were students, fulfilling domestic tasks or in compulsory service, had no limitations in activities due to a health problem and were less likely to be overweight or smoke daily.

Statistical data analysis

We conducted separate analyses for each component of unmet need and investigated associations between the outcome and explanatory variables using multivariate logistic regression models of the following form:

$$P(y=1|x) = \frac{e^{\alpha + \beta X}}{1 + e^{(\alpha + \beta X)}}$$

where y is unmet need due to wait, distance or affordability and X is a vector of explanatory variables.

We used sample weights to ensure the sample was representative of the population in terms of age, sex and district of residence. We estimated models with robust standard errors. As a sensitivity analysis, following Bataineh et al. [50], we included a binary variable equal to one for observations with missing data for income in all models. We reported results for all models as odds ratios adjusted for the explanatory variables with 95% confidence intervals. We estimated all models using Stata Version 15 [51].

For peer review only - http://bmjopen.bmj.com/site/about/guidelines.xhtml

Patient and public involvement

Patients or the public were not involved in the design, conduct, reporting or dissemination of the EHIS.

Results

Table 2 shows the sample size for each component of unmet need and the weighted percentage of unmet need. The most commonly cited barrier to access was due to wait times (32%) while the least common was distance (4%). 15% of respondents reported unmet need due to affordability of care. Dental care (12%) was most commonly cited as being unaffordable, followed by prescribed medicines (6%), medical (5%) and mental health care (5%).

Insert Table 2 around here

Table 3 presents the number and weighted percentage of respondents according to the explanatory variables for each component of unmet need. A higher proportion of respondents who had bad or very bad health, a chronic disease, limitations in activities due to health problems or were ex-drinkers reported unmet need for all components. A higher proportion of respondents with obesity, low social support, and whose income fell below the first quintile reported unmet need due to distance and financial barriers. There was no discernible pattern between the remaining respondent characteristics and types of unmet need.

Insert Table 3 around here

Table 4 displays the adjusted odds ratios from the multivariate logistic regression models. Respondents who reported bad or very bad health were associated with a higher risk of experiencing unmet need for every component, compared to those with good or very good health. Variables positively associated with reporting unmet need due to wait times included being female (OR 1.52, 95% CI 1.19-1.93), having a chronic condition (OR 1.45, 95% CI 1.11-1.91) and limitations in activities due to health problems (OR 1.33, 95% CI 1.02-1.73). Respondents aged 65 years and over were less likely to experience long wait times in comparison to the 15-24 age group (OR 0.44, 95% CI 0.21-0.90). A lower risk of experiencing delays due to distance or transportation problems was associated with the age groups 35-44 (OR 0.20, 95% CI 0.05-0.75), 45-54 (OR 0.04, 95% CI 0.01-0.23) and 55-64 (OR 0.14, 95% CI 0.03-0.63) as well as the income groups between the second and third quintiles (OR 0.19, 95% CI 0.06-0.54) and the fourth and fifth quintiles (OR 0.14, 95% CI 0.04-0.57). There is some consistency in determinants of unmet need arising from financial barriers for different types of care. Moderate or high social support was associated with a lower risk for dental care, prescribed medicines and mental health care. Married respondents were also associated with

For peer review only - http://bmjopen.bmj.com/site/about/guidelines.xhtml

a lower risk for prescribed medicines (OR 0.53, 95% CI 0.33-0.84) and mental health care (OR 0.40, 95% CI 0.21-0.79). Non-participation in the labour force due to studies, domestic work or compulsory service was associated with a higher risk of being unable to afford prescribed medicines (OR 2.22, 95% CI 1.04-4.76) and mental health care (OR 2.95, 95% CI 1.28-6.77). Income between the first and second quintiles was associated with a lower risk of unmet need for medical care (OR 0.37, 95% CI 0.18-0.78) and prescribed medicines (OR 0.49, 95% CI 0.26-0.95); income between the third and fourth quintiles was associated with a lower risk for dental care (OR 0.46, 95% CI 0.26-0.79) and prescribed medicines (OR 0.33, 95% CI 0.15-0.70); and income between the fourth and fifth quintiles was associated with a lower risk for dental care (OR 0.29, 95% CI 0.16-0.53), prescribed medicines (OR 0.38, 95% CI 0.17-0.82) and mental health care (OR 0.17, 95% CI 0.05-0.61). Daily smokers were associated with a higher risk of unmet need for dental (OR 1.82, 95% CI 1.28-2.60) and mental health care (OR 2.15, 95% CI 1.10-4.17) compared to non-smokers. Immigrants were at higher risk of being unable to afford prescribed medicines (OR 0.58, 95% CI 0.35-0.97).

Insert Table 4 around here

Table A3 in Appendix 2 of the supplementary data shows the results of the sensitivity analysis that included a binary variable measuring income non-response in each model. This variable was statistically significant in only the model for affordability of dental care with non-reporting of income associated with a lower risk of reporting unmet need due to the affordability of dental care (OR 0.64, 95% CI 0.42-0.97).Results for all models were largely unchanged. As in the main analyses, a lower risk of unmet need due to the affordability of dental care was associated with moderate (OR 0.43, 95% CI 0.24-0.78) or high (OR 0.40, 95% CI 0.22-0.72) social support, income between the third and fourth quintile (OR 0.48, 95% CI 0.28-0.81) and fourth and fifth quintile (OR 0.30, 95% CI 0.17-0.54) and regular alcohol consumption (OR 0.60, 95% CI 0.42-0.87).

Discussion

A statement of the principal findings

In this paper, we investigated the prevalence and determinants of unmet need in Luxembourg using EHIS 2 data. The most common barrier to accessing health care arose from long waits (32%). Compared to other EU countries who collected this data using the EHIS, Luxembourg had the highest proportion of respondents who reported unmet need due to long waits [52]. Respondents were most likely to report being unable to afford dental care (12%) followed by prescribed medicines (6%) – a result that is on par with the EU averages (12.3% and 4.6% respectively) [53]. The percentage of respondents who reported being unable to afford mental health care (5%) was higher than the EU average (2.7%) [53]. The association of various

For peer review only - http://bmjopen.bmj.com/site/about/guidelines.xhtml

determinants of unmet need varied according to the different components. However, bad or very bad self-reported health was positively associated with every component of unmet need. This group was twice as likely to report unmet need due to wait time, seven times more likely to report unmet need due to distance or transportation, and two to five times more likely to report unmet need due to affordability of services compared to respondents who assessed their health as good or very good. Another notable finding was the clear income gradient for unmet need due to the affordability of care, with the highest income quintile 71% less likely to report unmet need for dental care, 62% less likely for prescribed medications and 83% less likely for mental health care compared to the lowest income quintile.

Comparisons with previous studies

Previous studies [14, 16, 21, 23, 25] have investigated unmet need within countries using EU-SILC data. The use of the EHIS brought two important advantages that moved us beyond these studies. First, we undertook separate analyses of unmet need arising from long waits, distance or transportation problems, and affordability of medical, dental and mental health care and prescribed medicines. Second, we exploited a richer set of health variables, which enabled us to investigate risk factors including BMI, smoking and alcohol consumption as determinants of unmet need. Moreover, this is the first study that used the EHIS to investigate the determinants of unmet need in the general population. Previous studies used the EHIS to investigate unmet need for specific population groups. Sakellariou et al. [30] investigated whether people with disabilities had higher unmet need for health care due to waiting times, distance or transport problems and unaffordability of medical and mental health care and prescribed medicines compared to people without disabilities in the UK. Rotarou et al. [29] conducted a similar study for Greece. Hoebel et al. [28] focused only on respondents aged 50-85 years of age in Germany to investigate unmet need due to the affordability of medical, dental and mental health care and prescribed medicines. They did not consider long waits or distance or transportation problems.

Three previous studies [14, 19, 23] used EU-SILC data on reasons for unmet need to investigate associations with various determinants. Two studies [14, 23] considered the main reason for unmet need due to availability (waiting lists) and accessibility (affordability and distance/transportation) while the remaining study [19] considered affordability and distance/transportation as separate categories. Comparable to our results, these studies reported a positive association between bad and very bad health (compared to very good) and unmet need due to availability [14] and accessibility [14, 23] or only affordability of care [19]. One study [19] found that social support as measured by the ability to seek help was associated with a lower probability of unmet needs for medical care arising from economic

For peer review only - http://bmjopen.bmj.com/site/about/guidelines.xhtml

costs, reflecting our finding that higher levels of social support were associated with lower unmet need due to affordability of care. Our result that females were associated with higher unmet need due to wait times was not supported by these studies, which found evidence of higher unmet need for females due to accessibility [14] and affordability of care [19]. Women and respondents with bad or very bad self-assessed health may have been unable to afford mental health care because they have higher need. A previous study [49] investigated the burden of depression in Luxembourg and reported a higher prevalence rate of depression symptoms in females compared to males and in those who perceived their health as poor, compared to those who perceived their health as good. This study also found that good social support was associated with a lower risk of depression, which could indicate a lower need for mental health care and hence lower unmet need arising from the affordability of mental health care.

Similar to previous studies [14, 23], we found that higher income was associated with a lower risk of unmet need due to distance and affordability of services. Income disparities in unmet need due to the affordability of services may have reflected the requirement for patients to pay the full costs of many services directly to providers at the point of use. Our finding of no statistically significant relationship between income and unmet need due to waits was also reflected in previous studies [14, 16, 19, 23]. Although we did not find any association between unmet need due to waits and socio-economic variables including education, income and job status, previous studies reported evidence of inequalities in waiting times related to education and income [54-56]. The EHIS data allowed us to consider the association between health behaviours and unmet need. We found that these variables were only associated with unmet need arising from the affordability of health care. Therefore, our results add to a previous study from Canada [50] that considered the relationship between obesity, drinking and smoking and unmet need due to health system factors (including unavailability of services and long waiting times and excluding cost) and found evidence of a positive association between smoking (both daily and occasional) and unmet need.

Study limitations

Our study is subject to limitations arising from our data, some of which could in principle be overcome in future research, via development and adjustment of the EHIS questionnaire. As unmet need was self-reported, the data may suffer from the limitations inherent in survey data, including recall or response bias (respondent inaccurately remembers or misunderstands the question) [57, 58]. Moreover, self-reported unmet need may be influenced by unobservable factors such as cultural norms, health literacy and expectations of health services [15, 20, 59, 60]. The EHIS did not ask respondents if they perceived long waits across the health care

For peer review only - http://bmjopen.bmj.com/site/about/guidelines.xhtml

system or for particular services (primary care, cancer care, elective treatment, diagnostic tests), nor the length of time perceived as long. The survey sampling did not include sections of society who lack any insurance coverage and may have high unmet need including the homeless and irregular immigrants and their families [34]. Individuals living in collective households (for example, a boarding house or hostel or a dormitory in an educational establishment) [43] and institutions were also excluded. Despite these limitations, survey data remains the best available source [59]. We excluded observations with missing data for household income. The profile of respondents who did not report household income indicated that they were young people in full-time education who may have been living in the family home. Perhaps these respondents did not know the value of the household income, because they were not a main earner. We could assume that some of these respondents were less likely to bear the financial costs of care and therefore less likely to report unmet need due to the affordability of care. Indeed, the results of the sensitivity analysis as shown in Table A3 of Appendix 2 suggested this was the case, although the variable measuring income non-response is only statistically significant in the model measuring affordability of dental care.

Implications for clinicians and policymakers

The Luxembourgish health system is characterised by a high rate of population health insurance coverage, a comprehensive benefit package and a relatively low level of out-of-pocket payments. Nevertheless, access to health care is an important issue, particularly in relation to wait times, as reflected in our finding that the majority of respondents reported unmet need due to long waiting times to obtain an appointment. While Luxembourg has introduced reforms to address waiting times in emergency departments and for cancer care, there is potential to implement measures that could address waiting times for other priority services where patients must wait to obtain an appointment, for example primary care. Unlike Luxembourg, in many European countries, nurses or physician assistants play a key role in primary care by providing health education, immunisations and routine checks of people with chronic illnesses [8], allowing GPs more time to attend to patients with more complex needs. Policymakers could also consider the introduction of a maximum waiting time for specialist consultations, a policy adopted by several European countries.

In response to the COVID-19 pandemic, teleconsultations are now reimbursed by the CNS. The retention and embedding of telemedicine into the health care system in Luxembourg could help to reduce unmet need due to distance and transportation, particularly for those with poor health who may face difficulties in travelling to health care providers. The increased use of teleconsultations could also help to address waiting times for particular services, for example, primary and specialist care.

For peer review only - http://bmjopen.bmj.com/site/about/guidelines.xhtml

While the introduction of a universal third-party payment system may help to address unmet need due to the affordability of care this reform may not be sufficient by itself as individuals may still face a financial barrier to care due to co-payments. For example, while prescribed medicines are currently covered by the third party payment system, meaning that individuals only pay the co-payment, our results show that 6% of respondents reported unmet need to affordability of prescribed medicines. Therefore, additional reforms aimed at reducing the risk of unmet need could address co-payments and target high-risk groups such as those with low incomes or in poor health, similar to policies in Belgium and France (as described in the Introduction). Currently the CNS does not reimburse visits to psychologists and expanding health insurance coverage to psychologists could help to address unmet need due to the affordability of mental health care, especially for at-risk groups including individuals who are female, aged 25-34, are students or undertaking domestic duties or compulsory service and with low income.

Implications for future research

Future revisions of the EHIS could expand the social variables collected, for example by adding information on ethnicity or parents' country of birth. Additional questions on social support could help to distinguish between emotional, instrumental and informational support [19]. Future research could investigate the issue of waiting times in Luxembourg for different types of services in order to inform policy measures to reduce unmet need. Recent policies focused on the reorganisation of cancer care delivery and introduction of waiting time targets for cancer care could be evaluated. Given our finding of income disparities in unmet need due to the affordability of care, an evaluation of the implementation of the third-party payment system to date would inform its planned expansion and potential further adjustments. The collection of a third wave of EHIS data will enable an assessment of trends in unmet need in Luxembourg over time. This research also demonstrates that the EHIS is a useful resource that could be utilised for future within- and between-country analyses of unmet need for health care in the general population in European countries. While previous cross-country studies have examined patient and health system characteristics associated with unmet need across European countries for medical and dental care, the EHIS could complement these studies by extending the analyses to unmet need arising from the affordability of mental health care and prescribed medicines.

Conclusions

For peer review only - http://bmjopen.bmj.com/site/about/guidelines.xhtml

Compared to other EU countries, Luxembourg has high per capita public health care expenditures, a low share of total health spending financed by out-of-pocket payments and a comprehensive benefit package [61, 62]. Nevertheless, this study provides evidence that some individuals experienced difficulties accessing care, particularly due to long waits and affordability of care. Future reforms to improve access to health care should first target high-risk groups including those with low incomes and poor health. Future policies to address unmet need should also consider other vulnerable populations who lack formal health care coverage including the homeless, undocumented immigrants and those in the informal labour market.

Authors' contribution:

Valerie Moran: Conceptualisation, Methodology, Software, Formal Analysis, Writing – Original Draft, Writing – Review and Editing, Visualisation. **Marc Suhrcke:** Writing – Review and Editing, **Maria Ruiz-Castell:** Writing – Review and Editing, Software, Resources, **Jessica Barré:** Data curation, **Laetitia Huiart:** Conceptualisation, Writing – Review and Editing, Supervision.

Funding: The EHIS Luxembourg study was funded by the Luxembourg Ministry of Health Directorate of Health and the Luxembourg Institute of Health. This research was supported by the Luxembourg National Research Fund (C19/BM/13723812).

Acknowledgements: We would like to thank to the population of Luxembourg and all the EHIS team who have contributed to this study. We would like to thank EHIS steering committee members S Couffignal, N de Rekeneire, S Leite, G Weber, AC Lorcy, G Osier and I Salagean for their valuable contributions. We thank colleagues for their feedback on earlier iterations of the research presented at seminars at the Luxembourg Institute of Health and Luxembourg Institute of Socio-Economic Research. We also thank the reviewers for their valuable comments.

Competing interests: Jessica Barré was formerly an employee of the Luxembourg Institute of Health and is currently employed at the *Caisse National de Santé* (CNS). The views presented are those of the authors alone and not of the CNS.

Data Sharing Statement: Information on the data access procedure for the 2014 EHIS for Luxembourg is available on the Ministry of Health Directorate of Health website: https://sante.public.lu/fr/statistiques/ehis/ehis-methodologie/ehis-formulaire-demande-de-donnees.docx

Ethics approval: Luxembourg's National Ethics Committee, the Comité National d'Ethique de

For peer review only - http://bmjopen.bmj.com/site/about/guidelines.xhtml

Recherche (CNER) was informed by the Luxembourg Ministry of Health Directorate of Health of the national regulation and European obligation to collect EHIS data. The survey was completely anonymous and no information on name, address or date of birth of respondents was collected.

For peer review only

For peer review only - http://bmjopen.bmj.com/site/about/guidelines.xhtml

References

1. WHO. *Health systems: Universal health coverage*. 2020; Available from: https://www.who.int/healthsystems/universal_health_coverage/en/#:~:text=Universal%20health%20coverage%20is%20defined,the%20user%20the%20financial%20hardship.
2. European Commission. *European Pillar of Social Rights* 2021; Available from: https://ec.europa.eu/info/strategy/priorities-2019-2024/economy-works-people/jobs-growth-and-investment/european-pillar-social-rights_en#background.
3. European Commission. *The European Pillar of Social Rights in 20 principles*. 2021; Available from: https://ec.europa.eu/info/strategy/priorities-2019-2024/economy-works-people/jobs-growth-and-investment/european-pillar-social-rights/european-pillar-social-rights-20-principles_en.
4. Gulliford, M., et al., *What does 'access to health care' mean?* J Health Serv Res Policy, 2002. **7**(3): p. 186-8.
5. European Union, *EXPH (EXpert Panel on effective ways of investing in Health). Opinion on Benchmarking Access to Healthcare in the EU*. 2017, European Union: Brussels.
6. Eurostat. *Methodological guidelines and description of EU-SILC target variables PH010: General health*. 2017; Available from: https://ec.europa.eu/eurostat/documents/1012329/8655367/PERSONAL+DATA_Health.pdf/0a942278-dd03-47f6-9af6-3f3000f678ce.
7. Allin, S. and C. Masseria, *Unmet need as an indicator of health care access.* Eurohealth, 2009. **15**(3): p. 7-10.
8. OECD, *Waiting Times for Health Services Next in Line*, in *OECD Health Policy Studies*. 2020, OECD Publishing: Paris.
9. Bouckaert N., M.d.N., C., Van de Voorde, C., *Health System Performance Assessment: How Equitable is the Belgian System?* 2020, Belgian Health Care Knowledge Centre (KCE): Brussels.
10. Goujard, A., *France: improving the efficiency of the healthcare system*, in *OECD Economics Department Working Papers No. 1455*. 2018, OECD Publishing: Paris.
11. OECD and European Observatory on Health Systems and Policies, *France: Country Health Profile 2019, State of Health in the EU,*. 2019: OECD Publishing, Paris/European Observatory on Health Systems and Policies, Brussels.
12. European Union, *Expert Panel on effective ways of investing in Health (EXPH), Report on Access to Health Services in the European Union*. 2016, European Commission: Brussels.
13. OECD, *Health for Everyone?: Social Inequalities in Health and Health Systems*, in *OECD Health Policy Studies*. 2019, OECD Publishing: Paris.
14. Cavalieri, M., *Geographical variation of unmet medical needs in Italy: a multivariate logistic regression analysis.* International journal of health geographics, 2013. **12**: p. 27-27.
15. Chaupain-Guillot, S. and O. Guillot, *Health system characteristics and unmet care needs in Europe: an analysis based on EU-SILC data.* Eur J Health Econ, 2015. **16**(7): p. 781-96.
16. Connolly, S. and M.-A. Wren, *Unmet healthcare needs in Ireland: Analysis using the EU-SILC survey.* Health Policy, 2017. **121**(4): p. 434-441.
17. Detollenaere, J., et al., *Do We Reap What We Sow? Exploring the Association between the Strength of European Primary Healthcare Systems and Inequity in Unmet Need.* PloS one, 2017. **12**(1): p. e0169274-e0169274.
18. Elstad, J.I., *Income inequality and foregone medical care in Europe during The Great Recession: multilevel analyses of EU-SILC surveys 2008-2013.* International journal for equity in health, 2016. **15**(1): p. 101-101.

For peer review only

19. Fiorillo, D., *Reasons for unmet needs for health care: the role of social capital and social support in some western EU countries.* Int J Health Econ Manag, 2019.
20. Israel, S., *How social policies can improve financial accessibility of healthcare: a multi-level analysis of unmet medical need in European countries.* Int J Equity Health, 2016. **15**: p. 41.
21. Legido-Quigley, H., et al., *Effects of the financial crisis and Troika austerity measures on health and health care access in Portugal.* Health Policy, 2016. **120**(7): p. 833-839.
22. Madureira-Lima, J., et al., *The Great Recession and inequalities in access to health care: a study of unemployment and unmet medical need in Europe in the economic crisis.* Int J Epidemiol, 2018. **47**(1): p. 58-68.
23. Popovic, N., et al., *Predictors of unmet health care needs in Serbia; Analysis based on EU-SILC data.* PLoS One, 2017. **12**(11): p. e0187866.
24. Reeves, A., et al., *Public pensions and unmet medical need among older people: cross-national analysis of 16 European countries, 2004-2010.* J Epidemiol Community Health, 2017. **71**(2): p. 174-180.
25. Yardim, M.S. and S. Uner, *Equity in access to care in the era of health system reforms in Turkey.* Health Policy, 2018. **122**(6): p. 645-651.
26. Fjær, E.L., et al., *Subjective perceptions of unmet need for health care in Europe among social groups: Findings from the European social survey (2014) special module on the social determinants of health.* Eur J Public Health, 2017. **27**(suppl_1): p. 82-89.
27. Quintal, C., et al., *No unmet needs without needs! Assessing the role of social capital using data from European social survey 2014.* Health Policy, 2019. **123**(8): p. 747-755.
28. Hoebel, J., et al., *Socioeconomic Inequalities in Health and Perceived Unmet Needs for Healthcare among the Elderly in Germany.* Int J Environ Res Public Health, 2017. **14**(10).
29. Rotarou, E.S. and D. Sakellariou, *Access to health care in an age of austerity: disabled people's unmet needs in Greece.* Critical Public Health, 2019. **29**(1): p. 48-60.
30. Sakellariou, D. and E.S. Rotarou, *Access to healthcare for men and women with disabilities in the UK: secondary analysis of cross-sectional data.* BMJ Open, 2017. **7**(8): p. e016614.
31. CNS d'Gesondheetskees, *Rapport annuel 2018*. 2019, CNS d'Gesondheetskees: Luxembourg.
32. Statec. *Le Portail des Statistiques Grand-Duché de Luxembourg* 2020; Available from:https://statistiques.public.lu/stat/TableViewer/tableView.aspx?ReportId=12853&IF_Language=fra&MainTheme=2&FldrName=1.
33. Berthet, F., et al., *Health Systems in Transition Luxembourg*. 2015, European Observatory on Health Systems and Policies: Brussels.
34. Médecins du Monde, *La santé pour tous. Rapport annuel 2018 Médecins du Monde Luxembourg* 2019, Médecins du Monde Luxembourg Luxembourg.
35. CNS d'Gesondheetskees. *Reimbursement*. 2019; Available from: https://cns.public.lu/en/assure/remboursement-prise-charge.html.
36. OECD. *Health Systems Characteristics Survey*. Available from: https://qdd.oecd.org/subject.aspx?Subject=hsc.
37. Institut national d'assurance maladie-invalidité. *Tiers payant : ne payez que votre part, la mutualité paie directement le dispensateur de soins*. 2017.
38. CNS d'Gesondheetskees. *Third-party social payment*. 2020; Available from: https://cns.public.lu/en/professionnels-sante/medecins/tiers-payant-social.html.
39. Government of Luxembourg. *Réunion du comité quadripartite en matière d'assurance maladie-maternité Communiqué*. 2019; Available from: https://gouvernement.lu/fr/actualites/toutes_actualites/communiques/2019/11-novembre/13-reunion-quadripartite.html.

For peer review only - http://bmjopen.bmj.com/site/about/guidelines.xhtml

40. Luxembourg Ministry of Health and Luxembourg Ministry of Social Security, *Étude du fonctionnement des services d'urgence*. 2017, Luxembourg Ministry of Health and Luxembourg Ministry of Social Security: Luxembourg.
41. Luxembourg Ministry of Health and Luxembourg Ministry of Social Security. *Communiqué - Conférence de presse (15.12.2017) Vers une amélioration des services d'urgence*. 2017; Available from: https://sante.public.lu/fr/actualites/2017/12/urgences/urgences.pdf.
42. CNS d'Gesondheetskees. *Treatment abroad*. 2016; Available from: https://cns.public.lu/en/assure/vie-privee/a-etranger.html.
43. Eurostat and European Commission, *European Health Interview Survey (EHIS wave 2) Methodological manual*. 2013, Publications Office of the European Union: Luxembourg.
44. Robert Koch-Institut, *European Health Interview Survey (EHIS) 2 – Background and study methodology.* Journal of Health Monitoring, 2019. **4**(4): p. 66--79.
45. Santé.lu. *European Health Interview Survey (EHIS)*. 2018; Available from: https://sante.public.lu/fr/statistiques/ehis/index.html.
46. Luxembourg Ministry of Health Directorate of Health and Luxembourg Institute of Health, *European Health Interview Survey (EHIS) Study Methodology*. 2017, Luxembourg Ministry of Health Directorate of Health and Luxembourg Institute of Health: Luxembourg
47. Bejko, D., et al., *"To survey or to register" is that the question for estimating population incidence of injuries?* Archives of Public Health, 2018. **76**(1): p. 76.
48. Eurostat, *Quality report of the second wave of the European Health Interview survey*. 2018, European Union: Luxembourg: Publications Office of the European Union.
49. Ruiz-Castell, M., et al., *Depression burden in luxembourg: Individual risk factors, geographic variations and the role of migration, 2013-2015 European Health Examination Survey.* J Affect Disord, 2017. **222**: p. 41-48.
50. Bataineh, H., R.A. Devlin, and V. Barham, *Unmet health care and health care utilization.* Health Economics, 2019. **28**(4): p. 529-542.
51. StataCorp., *Stata Statistical Software: Release 15.* 2017, StataCorp LLC.: College Station, TX.
52. Eurostat. *Self-reported unmet needs for health care by sex, age, specific reasons and educational attainment level* 2020; Available from: https://appsso.eurostat.ec.europa.eu/nui/show.do?dataset=hlth_ehis_un1e&lang=en.
53. Eurostat. *Self-reported unmet needs for specific health care-related services due to financial reasons by sex, age and educational attainment level*. 2020; Available from: https://appsso.eurostat.ec.europa.eu/nui/show.do?dataset=hlth_ehis_un2e&lang=en.
54. Simonsen, N.F., et al., *What explains differences in waiting times for health care across socioeconomic status?* Health Economics, 2020. **29**(12): p. 1764-1785.
55. Siciliani, L. and R. Verzulli, *Waiting times and socioeconomic status among elderly Europeans: evidence from SHARE.* Health Econ, 2009. **18**(11): p. 1295-306.
56. Laudicella, M., L. Siciliani, and R. Cookson, *Waiting times and socioeconomic status: evidence from England.* Soc Sci Med, 2012. **74**(9): p. 1331-41.
57. Arora, V.S., et al., *Data Resource Profile: The European Union Statistics on Income and Living Conditions (EU-SILC).* Int J Epidemiol, 2015. **44**(2): p. 451-61.
58. Iezzoni, I., *Risk adjustment for performance measurement*, in *Performance Measurement for Health System Improvement*, P.C. Smith, et al., Editors. 2009, Cambridge University Press: Cambridge.
59. Baeten, R., et al., *Inequalities in access to healthcare. A study of national policies*. 2018, European Commission: Brussels.
60. Eurostat, *Methodological issues in the analysis of the socioeconomic determinants of health using EU-SILC data*, in *Eurostat Methodologies and Working Papers*. 2010, Publications Office of the European Union: Luxembourg.
61. OECD, *OECD Health Statistics 2020*. 2020, OECD Publishing: Paris.

For peer review only - http://bmjopen.bmj.com/site/about/guidelines.xhtml

62. OECD and European Observatory on Health Systems and Policies, *Luxembourg: Country Health Profile 2019, State of Health in the EU*. 2019: OECD Publishing, Paris/European Observatory on Health Systems and Policies, Brussels.

For peer review only

For peer review only - http://bmjopen.bmj.com/site/about/guidelines.xhtml

Table 1. Responses for the six components of unmet need

	Wait		Distance or transportation problems		Could not afford medical care		Could not afford dental care		Could not afford prescribed medicines		Could not afford mental health care	
	N	%	N	%	N	%	N	%	N	%	N	%
No	1,906	48	2,482	62	2,866	72	2,809	70	2,977	74	2,097	52
Yes	878	22	92	2	182	5	399	10	216	5	106	3
No need for health care	1,043	26	983	25	782	20	599	15	609	15	1,533	38
Missing	177	4	447	11	174	4	197	5	202	5	268	7
**Total**	**4,004**	**100**	**4,004**	**100**	**4,004**	**100**	**4,004**	**100**	**4,004**	**100**	**4,004**	**100**

For peer review only - http://bmjopen.bmj.com/site/about/guidelines.xhtml

Table 2 Prevalence of unmet need

Variable	N	N, unmet need = 1	%
Wait	1,639	529	32
Distance or transportation problems	1,531	51	4
Could not afford medical care	1,813	94	5
Could not afford dental care	1,888	222	12
Could not afford prescribed medicines	1,899	108	6
Could not afford mental health care	1,268	63	5

Note: N refers to number of respondents in estimation sample, N, unmet need =1 refers to the number of respondents in the estimation sample who reported an unmet need for each component and % is the percentage of estimation sample who reported an unmet need for each component weighted for age, sex and district of residence.

For peer review only

For peer review only - http://bmjopen.bmj.com/site/about/guidelines.xhtml

Table 3. Characteristics of respondents reporting an unmet need due to wait, distance or affordability

	Wait (n=1,639)		Distance (n=1,531)		Could not afford medical care (n=1,813)		Could not afford dental care (n=1,888)		Could not afford prescribed medicines (n=1,899)		Could not afford mental health care (n=1,268)	
**Variable**	**N**	**(%)**	**N**	**(%)**	**N**	**(%)**	**N**	**(%)**	**N**	**(%)**	**N**	**(%)**
Sex												
Male	215	(45)	27	(57)	44	(51)	106	(52)	63	(61)	19	(35)
Female	314	(55)	24	(43)	50	(49)	116	(48)	45	(39)	44	(65)
Age												
15-24	35	(8)	10	(24)	6	(8)	15	(8)	7	(8)	4	(7)
25-34	119	(23)	11	(20)	16	(17)	46	(21)	21	(19)	22	(36)
35-44	119	(22)	10	(18)	20	(20)	46	(20)	12	(11)	15	(23)
45-54	119	(21)	3	(5)	25	(26)	55	(25)	31	(28)	10	(16)
55-64	78	(13)	6	(12)	14	(14)	37	(15)	16	(14)	11	(17)
65 and over	59	(12)	11	(21)	13	(15)	23	(11)	21	(20)	1	(1)
Marital status												
No	187	(37)	24	(50)	43	(46)	97	(45)	51	(50)	38	(62)
Yes	342	(63)	27	(50)	51	(54)	125	(55)	57	(50)	25	(38)
Immigrant												
No	322	(62)	29	(56)	60	(65)	134	(61)	80	(73)	31	(51)
Yes	207	(38)	22	(44)	34	(35)	88	(39)	28	(27)	32	(49)
Education												

For peer review only - http://bmjopen.bmj.com/site/about/guidelines.xhtml

Primary and pre-primary	34	(7)	5	(9)	11	(12)	18	(8)	12	(12)	5	(7)
Secondary & post-secondary	264	(50)	27	(54)	66	(71)	146	(66)	76	(71)	37	(61)
Tertiary	231	(43)	19	(37)	17	(17)	58	(25)	20	(18)	21	(32)
Job Status												
Employed	334	(62)	25	(47)	52	(54)	136	(61)	53	(47)	32	(50)
Unemployed	15	(3)	1	(2)	8	(9)	13	(6)	5	(5)	7	(12)
Retired	91	(17)	12	(22)	16	(16)	32	(14)	24	(21)	4	(6)
Student, domestic, compulsory service	74	(15)	10	(23)	11	(14)	30	(14)	19	(20)	13	(21)
Permanently disabled and other inactive status	15	(3)	3	(6)	7	(7)	11	(5)	7	(7)	7	(12)
Social support												
Low	19	(4)	4	(7)	8	(8)	16	(7)	10	(9)	10	(16)
Moderate	228	(44)	24	(48)	39	(42)	84	(39)	37	(35)	35	(55)
High	282	(52)	23	(45)	47	(50)	122	(54)	61	(56)	18	(29)
Household income												
Below 1st quintile (Lowest)	100	(19)	21	(40)	35	(37)	67	(30)	34	(32)	25	(40)
Between 1st & 2nd quintile	82	(16)	13	(30)	13	(15)	50	(23)	18	(17)	13	(20)
Between 2nd & 3rd quintile	133	(24)	6	(11)	24	(26)	57	(26)	33	(30)	15	(23)
Between 3rd & 4th quintile	108	(21)	7	(13)	12	(13)	29	(13)	12	(11)	7	(12)
Between 4th & 5th quintile (Highest)	106	(20)	4	(7)	10	(10)	19	(8)	11	(10)	3	(4)
Informal Carer												
No	419	(79)	43	(84)	75	(80)	183	(82)	88	(82)	53	(84)
Yes	110	(21)	8	(16)	19	(20)	39	(18)	20	(18)	10	(16)
BMI												

For peer review only - http://bmjopen.bmj.com/site/about/guidelines.xhtml

Normal or Underweight (<25)	286	(53)	23	(45)	47	(51)	93	(42)	42	(39)	28	(45)
Overweight (25-29)	141	(28)	14	(28)	19	(19)	78	(34)	33	(30)	19	(30)
Obese (>=30)	102	(19)	14	(27)	28	(30)	51	(23)	33	(31)	16	(25)
Smoker												
No	422	(80)	39	(76)	60	(65)	150	(67)	84	(78)	38	(60)
Occasionally	27	(5)	3	(7)	10	(10)	12	(5)	3	(2)	2	(3)
Daily	80	(15)	9	(18)	24	(24)	60	(28)	21	(20)	23	(37)
Alcohol consumption												
Never	102	(19)	13	(25)	22	(25)	55	(25)	23	(22)	14	(23)
Ex-drinkers	61	(11)	9	(18)	15	(16)	36	(17)	13	(12)	12	(20)
Irregularly	51	(9)	9	(15)	12	(12)	23	(10)	16	(15)	7	(10)
Regularly	180	(34)	10	(19)	27	(27)	52	(24)	34	(31)	19	(30)
Everyday	135	(26)	10	(23)	18	(20)	56	(25)	22	(20)	11	(16)
Self-assessed health												
Good/very good	325	(61)	17	(34)	40	(41)	113	(50)	56	(51)	29	(46)
Fair	142	(27)	20	(40)	26	(29)	74	(34)	31	(30)	18	(28)
Bad/Very bad	62	(12)	14	(26)	28	(30)	35	(16)	21	(19)	16	(26)
Chronic disease												
No	132	(25)	10	(20)	25	(26)	63	(28)	26	(32)	8	(13)
Yes	397	(75)	41	(80)	69	(74)	159	(72)	82	(68)	55	(87)
Limitations in activities due to health problems												
No limitations	272	(51)	17	(35)	36	(37)	106	(48)	44	(40)	24	(37)
Limited/severely limited	257	(49)	34	(65)	58	(63)	116	(52)	64	(60)	39	(63)
Canton												

For peer review only - http://bmjopen.bmj.com/site/about/guidelines.xhtml

Capellen	54	(10)	3	(6)	11	(12)	24	(10)	14	(12)	8	(12)
Clervaux	14	(3)	5	(9)	4	(5)	7	(4)	8	(8)	2	(3)
Diekirch	17	(3)	5	(11)	7	(8)	11	(6)	5	(5)	2	(3)
Echternach	14	(3)	2	(5)	4	(4)	9	(4)	3	(3)	2	(3)
Esch sur Alzette	143	(26)	12	(22)	30	(32)	70	(32)	29	(25)	18	(29)
Grevenmacher	37	(7)	5	(10)	6	(7)	15	(7)	9	(9)	4	(8)
Luxembourg	171	(32)	13	(26)	18	(18)	55	(23)	19	(17)	20	(31)
Mersch	37	(7)	1	(2)	5	(5)	10	(5)	9	(8)	1	(1)
Redange	16	(3)	1	(3)	3	(3)	4	(2)	2	(2)	1	(1)
Remich	17	(3)	2	(4)	3	(3)	9	(4)	5	(5)	3	(6)
Vianden	3	(1)	1	(2)	1	(1)	3	(1)	2	(2)	1	(2)
Wiltz	6	(1)	1	(2)	2	(3)	5	(2)	3	(3)	1	(1)

Note: N refers to number of respondents in estimation sample and (%) is percentage of estimation sample weighted for age, sex and district of residence.

view only

Table 4. Multivariate logistic regression models for unmet need due to wait, distance or affordability

Variable	Wait (n=1,639)	Distance (n=1,531)	Could not afford medical care (n=1,813)	Could not afford dental care (n=1,888)	Could not afford prescribed medicines (n=1,899)	Could not afford mental health care (n=1,268)
Sex						
Male	1.00	1.00	1.00	1.00	1.00	1.00
Female	1.52 (1.19-1.93)**	0.83 (0.40-1.75)	1.04 (0.64-1.71)	0.96 (0.69-1.33)	0.55 (0.35-0.88)*	2.89 (1.41-5.94)**
Age						
15-24	1.00	1.00	1.00	1.00	1.00	1.00
25-34	1.32 (0.72-2.40)	0.27 (0.07-1.02)	1.76 (0.49-6.27)	1.62 (0.71-3.71)	3.22 (0.98-10.57)	6.39 (1.75-23.35)**
35-44	1.04 (0.55-1.93)	0.20 (0.05-0.75)*	1.87 (0.51-6.81)	1.36 (0.58-3.16)	1.53 (0.40-5.86)	2.49 (0.62-9.97)
45-54	1.00 (0.54-1.83)	0.04 (0.01-0.23)***	2.09 (0.58-7.49)	1.53 (0.66-3.56)	3.65 (1.05-12.66)*	1.33 (0.31-5.60)
55-64	0.59 (0.31-1.12)	0.14 (0.03-0.63)*	1.55 (0.39-6.06)	1.51 (0.62-3.68)	2.24 (0.58-8.58)	2.18 (0.51-9.35)
65 and over	0.44 (0.21-0.90)*	0.27 (0.07-1.07)	2.28 (0.50-10.36)	1.21 (0.46-3.19)	2.98 (0.76-11.75)	0.17 (0.01-2.16)
Marital status						
No	1.00	1.00	1.00	1.00	1.00	1.00
Yes	1.16 (0.89-1.52)	1.14 (0.47-2.78)	0.73 (0.43-1.23)	0.76 (0.53-1.08)	0.53 (0.33-0.84)**	0.40 (0.21-0.79)**
Immigrant						
No	1.00	1.00	1.00	1.00	1.00	1.00
Yes	0.79 (0.61-1.01)	1.18 (0.55-2.52)	0.79 (0.49-1.26)	0.85 (0.61-1.19)	0.58 (0.35-0.97)*	1.37 (0.68-2.76)
Education						
Primary and pre-primary	1.00	1.00	1.00	1.00	1.00	1.00
Secondary & post-secondary	1.14 (0.71-1.85)	1.26 (0.42-3.79)	1.48 (0.61-3.55)	1.6 (0.85-3.02)	1.35 (0.64-2.85)	1.55 (0.45-5.35)

For peer review only - http://bmjopen.bmj.com/site/about/guidelines.xhtml

Tertiary	1.40 (0.82-2.37)	2.59 (0.80-8.41)	0.55 (0.20-1.53)	1.2 (0.59-2.44)	0.67 (0.27-1.65)	1.22 (0.29-5.11)
Job Status						
Employed	1.00	1.00	1.00	1.00	1.00	1.00
Unemployed	0.62 (0.31-1.25)	0.21 (0.04-1.11)	1.74 (0.59-5.11)	1.4 (0.65-3.02)	1.51 (0.48-4.75)	1.51 (0.45-5.09)
Retired	1.22 (0.76-1.95)	0.91 (0.29-2.83)	0.6 (0.23-1.54)	0.58 (0.32-1.05)	0.88 (0.38-2.02)	1.2 (0.30-4.71)
Student, domestic, compulsory service	1.25 (0.82-1.91)	0.66 (0.23-1.85)	0.81 (0.33-2.01)	1.01 (0.58-1.76)	2.22 (1.04-4.76)*	2.95 (1.28-6.77)*
Permanently disabled and other inactive status	0.80 (0.38-1.69)	1.6 (0.29-8.81)	0.92 (0.35-2.41)	0.9 (0.44-1.84)	1.4 (0.58-3.37)	2.97 (0.92-9.64)
Social support						
Low	1.00	1.00	1.00	1.00	1.00	1.00
Moderate	1.54 (0.81-2.92)	0.84 (0.26-2.72)	0.6 (0.22-1.66)	0.46 (0.23-0.95)*	0.32 (0.14-0.77)*	0.27 (0.09-0.76)*
High	0.95 (0.50-1.81)	0.64 (0.19-2.22)	0.44 (0.16-1.23)	0.42 (0.21-0.86)*	0.30 (0.13-0.70)**	0.11 (0.03-0.35)***
Household income						
Below 1st quintile (Lowest)	1.00	1.00	1.00	1.00	1.00	1.00
Between 1st & 2nd quintile	0.85 (0.57-1.26)	0.74 (0.31-1.76)	0.37 (0.18-0.78)**	0.77 (0.48-1.21)	0.49 (0.26-0.95)*	0.49 (0.22-1.12)
Between 2nd & 3rd quintile	1.01 (0.70-1.48)	0.19 (0.06-0.54)**	0.73 (0.40-1.36)	0.77 (0.49-1.21)	0.77 (0.43-1.36)	0.78 (0.35-1.72)
Between 3rd & 4th quintile	1.16 (0.77-1.73)	0.35 (0.11-1.10)	0.47 (0.22-1.01)	0.46 (0.26-0.79)**	0.33 (0.15-0.70)**	0.47 (0.17-1.29)
Between 4th & 5th quintile (Highest)	0.91 (0.59-1.38)	0.14 (0.04-0.57)**	0.46 (0.20-1.07)	0.29 (0.16-0.53)***	0.38 (0.17-0.82)*	0.17 (0.05-0.61)**
Informal Carer						
No	1.00	1.00	1.00	1.00	1.00	1.00
Yes	1.36 (1.02-1.82)*	0.92 (0.42-2.03)	1.17 (0.67-2.03)	0.96 (0.63-1.45)	0.91 (0.53-1.56)	0.72 (0.32-1.64)
BMI						
Underweight or Normal	1.00	1.00	1.00	1.00	1.00	1.00
Overweight	0.81 (0.62-1.06)	1.04 (0.49-2.22)	0.49 (0.27-0.90)*	1.28 (0.90-1.83)	1.17 (0.69-1.99)	1.92 (0.94-3.91)
Obese	0.99 (0.72-1.35)	1.24 (0.61-2.55)	1.09 (0.62-1.92)	1.35 (0.89-2.02)	1.72 (0.96-3.08)	1.49 (0.68-3.27)

For peer review only - http://bmjopen.bmj.com/site/about/guidelines.xhtml

Smoker (No)						
No	1.00	1.00	1.00	1.00	1.00	1.00
Occasionally	1.06 (0.64-1.74)	1.38 (0.43-4.43)	2.76 (1.27-5.99)*	0.96 (0.49-1.86)	0.39 (0.10-1.44)	0.35 (0.07-1.64)
Daily	0.91 (0.66-1.26)	0.87 (0.38-1.96)	1.25 (0.72-2.15)	1.82 (1.28-2.60)**	0.90 (0.54-1.50)	2.15 (1.10-4.17)*
Alcohol consumption						
Never	1.00	1.00	1.00	1.00	1.00	1.00
Ex-drinkers	1.49 (0.95-2.35)	1.09 (0.41-2.90)	1.33 (0.59-3.01)	1.6 (0.93-2.77)	1.1 (0.49-2.49)	1.92 (0.73-5.08)
Irregularly	1.06 (0.68-1.63)	1.68 (0.64-4.38)	1.22 (0.54-2.73)	0.89 (0.51-1.55)	1.19 (0.59-2.41)	1.27 (0.40-4.04)
Regularly	1.12 (0.81-1.55)	0.75 (0.29-1.94)	0.89 (0.48-1.65)	0.63 (0.41-0.97)*	0.97 (0.54-1.74)	1.36 (0.59-3.17)
Everyday	1.07 (0.75-1.53)	0.97 (0.37-2.53)	0.85 (0.41-1.74)	1.02 (0.65-1.59)	0.8 (0.41-1.56)	1.2 (0.49-2.98)
Self-assessed health						
Good/very good	1.00	1.00	1.00	1.00	1.00	1.00
Fair	1.21 (0.90-1.62)	2.45 (1.17-5.12)*	1.26 (0.66-2.43)	1.63 (1.10-2.40) *	0.9 (0.52-1.55)	1.01 (0.44-2.28)
Bad/Very bad	2.41 (1.53-3.80)***	7.12 (2.91-17.44)***	5.35 (2.39-11.95)***	3.26 (1.86-5.71)***	2.22 (1.04-4.71)*	3.58 (1.25-10.30)*
Chronic disease						
No	1.00	1.00	1.00	1.00	1.00	1.00
Yes	1.45 (1.11-1.91)**	1.42 (0.60-3.37)	0.69 (0.38-1.28)	0.83 (0.57-1.21)	0.84 (0.48-1.44)	2.33 (0.89-6.08)
Limitations in activities due to health problems						
No limitations	1.00	1.00	1.00	1.00	1.00	1.00
Limited/severely limited	1.33 (1.02-1.73)*	1.37 (0.67-2.81)	1.39 (0.74-2.64)	1.15 (0.79-1.67)	1.49 (0.86-2.58)	1.53 (0.64-3.66)
Canton						
Esch zur Alzette	1.00	1.00	1.00	1.00	1.00	1.00
Capellen	1.21 (0.80-1.82)	1.08 (0.25-4.57)	1.42 (0.64-3.13)	1.41 (0.83-2.40)	2.12 (1.04-4.30)*	1.63 (0.61-4.37)
Clervaux	0.90 (0.46-1.75)	3.31 (0.78-14.14)	1.08 (0.31-3.81)	0.76 (0.31-1.88)	2.06 (0.77-5.53)	0.53 (0.12-2.31)

For peer review only - http://bmjopen.bmj.com/site/about/guidelines.xhtml

Diekirch	0.92 (0.49-1.72)	3.54 (1.02-12.32)	2.27 (0.87-5.95)	1.31 (0.60-2.83)	1.88 (0.68-5.18)	0.67 (0.12-3.85)
Echternach	1.43 (0.69-2.97)	2.02 (0.36-11.36)	1.63 (0.40-6.67)	1.47 (0.68-3.17)	1.13 (0.29-4.37)	1.19 (0.14-10.34)
Grevenmacher	1.67 (1.01-2.78)	3.06 (1.02-9.15)	1.38 (0.49-3.90)	1.27 (0.65-2.49)	2.53 (1.08-5.96)*	1.28 (0.30-5.51)
Luxembourg	1.27 (0.94-1.72)	1.4 (0.59-3.30)	0.8 (0.42-1.50)	0.99 (0.65-1.50)	1.02 (0.54-1.94)	1.54 (0.73-3.25)
Mersch	1.67 (1.01-2.76)*	0.43 (0.05-3.74)	1.11 (0.39-3.22)	0.88 (0.40-1.94)	2.38 (1.00-5.67)	0.29 (0.04-2.23)
Redange	1.07 (0.56-2.04)	0.95 (0.09-10.02)	0.91 (0.27-3.04)	0.53 (0.17-1.60)	0.60 (0.13-2.80)	0.80 (0.08-7.68)
Remich	0.99 (0.52-1.90)	1.64 (0.32-8.40)	0.75 (0.23-2.40)	1.05 (0.50-2.20)	1.51 (0.58-3.97)	3.45 (0.94-12.74)
Vianden	0.59 (0.14-2.46)	6.92 (0.62-77.07)	0.8 (0.14-4.45)	1.04 (0.29-3.67)	2.13 (0.58-7.77)	1.64 (0.24-11.19)
Wiltz	0.39 (0.15-0.99)	0.45 (0.07-3.16)	1.79 (0.33-9.75)	0.95 (0.34-2.71)	2.35 (0.57-9.65)	0.40 (0.05-3.14)
Constant	0.15 (0.05-0.42)***	0.08 (0.01-0.67)*	0.10 (0.02-0.57)*	0.22 (0.06-0.78)*	0.14 (0.03-0.59)**	0.01 (0.00-0.11)***

Note: Estimates are weighted for age, sex and district of residence. ***p < 0.001.**p < 0.01.*p < 0.05. Data are odds ratios adjusted for explanatory variables (95% confidence intervals)

For peer review only - http://bmjopen.bmj.com/site/about/guidelines.xhtml

Figure 1. Map of Luxembourg cantons

Source: https://d-maps.com/m/europa/luxembourg/luxembourg88.pdf

For peer review only

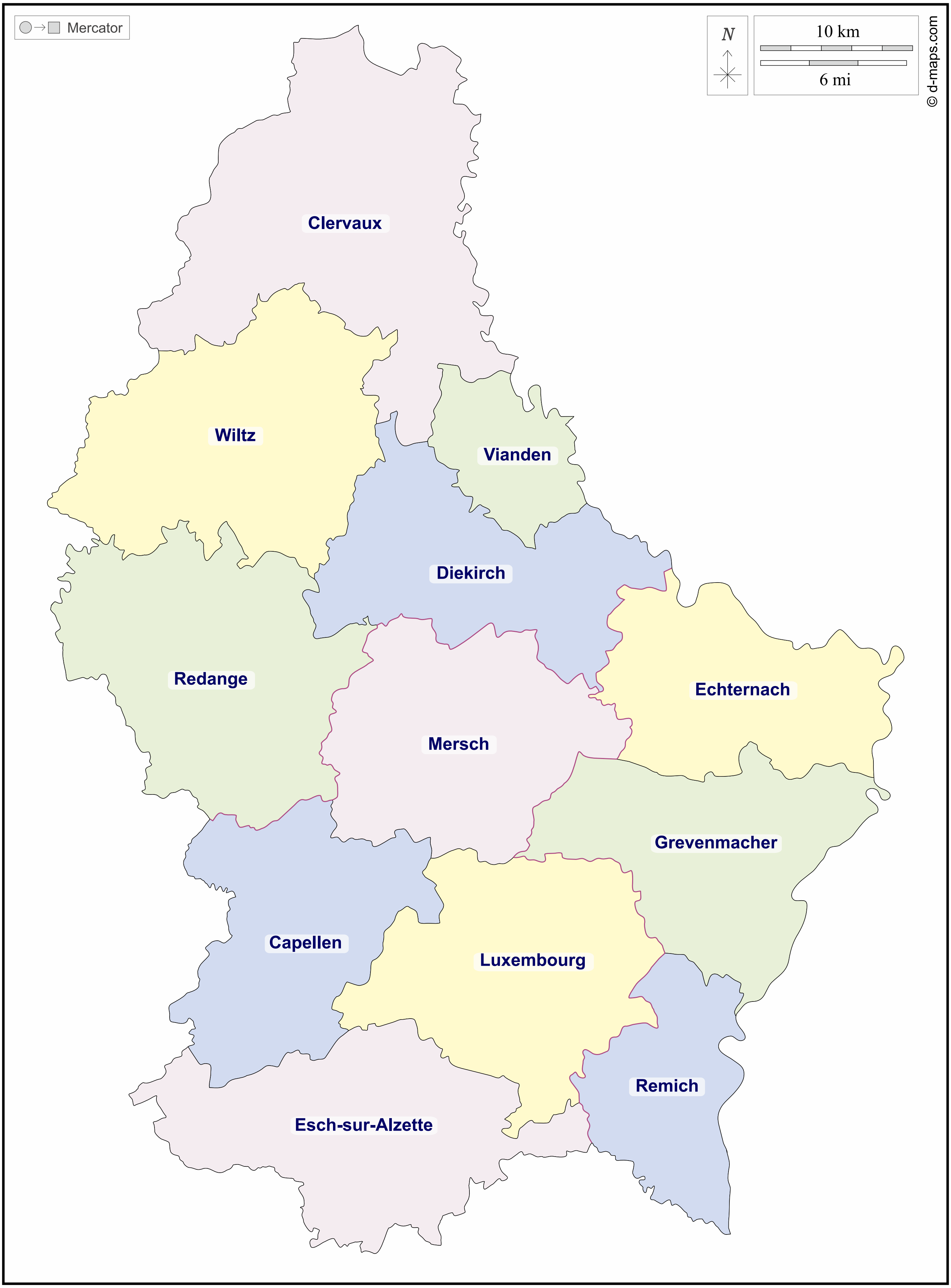
Mercator
N
10 km
6 mi
© d-maps.com
Clervaux
Wiltz
Vianden
Diekirch
Redange
Echternach
Mersch
Grevenmacher
Capellen
Luxembourg
Remich
Esch-sur-Alzette

Appendix 1. Unmet Needs for Health Care Questions

3.22. In the **past 12 months**, have you experienced delay in getting health care because/due to…?

	Yes	No	No need for healthcare
The time needed to obtain an appointment was too long	☐	☐	☐
Distance or transportation problems	☐	☐	☐

3.23. Was there any time in the **past 12 months** you needed the following kinds of care, but could not afford it?

	Yes	No	No need for healthcare
Medical care	☐	☐	☐
Dental care	☐	☐	☐
Prescribed medicines	☐	☐	☐
Mental health care (by a psychologist or a psychiatrist for example)	☐	☐	☐

Source: Santé.lu. European Health Interview Survey (EHIS) 2018. Available from: https://sante.public.lu/fr/statistiques/ehis/ehis-methodologie/ehis-questionnaire-en.pdf

For peer review only

Appendix 2.

Table A1. Missing data for independent variables

Variable	N	%
Sex	0	0
Age	0	0
Marital status	123	3.34
Immigrant	27	0.71
Education	182	4.82
Employment status	284	7.30
Household income	1,116	28.84
Social support	66	1.69
Informal carer	170	4.46
BMI	58	1.43
Smoker	44	1.11
Alcohol consumption	143	3.64
Self-assessed health	51	1.28
Chronic disease	70	1.75
Limitations in activities due to health problems	217	5.60
Canton	6	0.14

Note: N refers to number of respondents in estimation sample and % is percentage of estimation sample weighted for age, sex and district of residence.

For peer review only

Table A2. Characteristics of respondents with and without missing income data

	Missing income				
	No		Yes		
**Variable**	**N**	**(%)**	**N**	**(%)**	**p-value**
Sex					
Male	1,415	(77)	425	(23)	<0.001
Female	1,473	(68)	691	(32)	
Age					
15-24	221	(47)	247	(53)	<0.001
25-34	526	(77)	154	(23)	
35-44	588	(80)	151	(20)	
45-54	599	(74)	206	(26)	
55-64	480	(74)	172	(26)	
65 and over	474	(72)	186	(28)	
Marital status					
No	1,061	(69)	474	(31)	<0.001
Yes	1,763	(75)	583	(25)	
Immigrant					
No	1,757	(70)	751	(30)	<0.001
Yes	1,113	(76)	356	(24)	
Education					
Primary and pre-primary	268	(67)	133	(33)	<0.001
Secondary & post-secondary	1,514	(72)	581	(28)	
Tertiary	1,020	(77)	306	(23)	
Job Status					
Employed	1,619	(79)	429	(21)	<0.001
Unemployed	75	(75)	25	(25)	
Retired	616	(76)	194	(24)	
Student, domestic, compulsory service	345	(53)	301	(47)	
Permanently disabled and other inactive status	80	(69)	36	(31)	
Social support					
Low	108	(73)	39	(27)	0.95
Moderate	1,030	(73)	389	(27)	
High	1,716	(72)	656	(28)	
Informal Carer					
No	2,295	(73)	843	(27)	0.354
Yes	497	(71)	199	(29)	
BMI					
Normal or Underweight (<25)	1,407	(70)	603	(30)	0.002
Overweight (25-29)	904	(76)	290	(24)	
Obese (>=30)	530	(71)	212	(29)	
Smoker					
No	2,244	(71)	908	(29)	0.007
Occasionally	165	(72)	63	(28)	

Daily	450	(78)	130	(22)	
Alcohol consumption					
Never	622	(74)	220	(26)	0.054
Ex-drinkers	225	(67)	112	(33)	
Irregularly	301	(75)	98	(25)	
Regularly	948	(72)	370	(28)	
Everyday	714	(74)	251	(26)	
Self-assessed health					
Good/very good	1,952	(71)	784	(29)	0.065
Fair	721	(75)	242	(25)	
Bad/Very bad	191	(75)	63	(25)	
Chronic disease					
No	937	(71)	390	(29)	0.066
Yes	1,913	(73)	694	(27)	
Limitations in activities due to health problems					
No limitations	1,711	(72)	682	(29)	0.005
Limited/severely limited	1,055	(76)	339	(24)	
Canton					
Capellen	263	(69)	117	(31)	0.252
Clervaux	94	(72)	36	(28)	
Diekirch	123	(72)	49	(28)	
Echternach	72	(71)	29	(29)	
Esch zur Alzette	854	(73)	309	(27)	
Grevenmacher	170	(76)	54	(24)	
Luxembourg	835	(73)	316	(27)	
Mersch	165	(68)	76	(32)	
Redange	92	(63)	53	(37)	
Remich	113	(72)	45	(28)	
Vianden	23	(82)	5	(18)	
Wiltz	80	(76)	25	(24)	

Note: N refers to number of respondents and (%) is percentage of estimation sample weighted for age, sex and district of residence.

only

Table A3. Sensitivity analysis with dummy variable for non-reporting of income

Variable	Wait (n=2,141)	Distance (n=2,000)	Could not afford medical care (n=2,348)	Could not afford dental care (n=2,465)	Could not afford prescribed mediciness (n=2,462)	Could not afford mental health care (n=1,672)
Sex						
Male	1.00	1.00	1.00	1.00	1.00	1.00
Female	1.55 (1.25-1.92)***	0.81 (0.44-1.49)	0.80 (0.52-1.23)	0.94 (0.7-1.25)	0.54 (0.36-0.80)**	2.34 (1.29-4.25)**
Age						
15-24	1.00	1.00	1.00	1.00	1.00	1.00
25-34	1.13 (0.7-1.8)	0.29 (0.1-0.84)*	2.78 (0.99-7.82)	1.48 (0.78-2.80)	2.46 (1.04-5.84)*	3.28 (1.21-8.90)*
35-44	0.84 (0.51-1.37)	0.16 (0.05-0.51)**	2.94 (1.00-8.66)	1.25 (0.64-2.46)	1.26 (0.46-3.48)	1.34 (0.47-3.80)
45-54	0.76 (0.47-1.23)	0.09 (0.03-0.28)***	3.65 (1.28-10.41)*	1.42 (0.73-2.77)	2.49 (1.01-6.15)*	0.87 (0.29-2.59)
55-64	0.48 (0.29-0.79)**	0.14 (0.04-0.49)**	2.54 (0.80-8.12)	1.20 (0.58-2.45)	1.86 (0.68-5.12)	0.76 (0.24-2.45)
65 and over	0.37 (0.21-0.67)**	0.29 (0.09-0.95)*	2.16 (0.55-8.45)	0.87 (0.39-1.96)	2.14 (0.72-6.34)	0.17 (0.03-1.13)
Marital status						
No	1.00	1.00	1.00	1.00	1.00	1.00
Yes	1.13 (0.89-1.43)	1.13 (0.54-2.34)	0.64 (0.41-1.01)	0.77 (0.56-1.06)	0.56 (0.37-0.86)**	0.53 (0.29-0.96)*
Immigrant						
No	1.00	1.00	1.00	1.00	1.00	1.00
Yes	0.80 (0.64-1)	0.95 (0.5-1.82)	0.95 (0.63-1.44)	0.98 (0.72-1.33)	0.62 (0.40-0.96)*	1.41 (0.76-2.6)
Education						
Primary and pre-primary	1.00	1.00	1.00	1.00	1.00	1.00
Secondary & post-secondary (non-tertiary)	1.20 (0.8-1.82)	1.61 (0.58-4.46)	1.74 (0.81-3.72)	1.61 (0.94-2.78)	1.06 (0.59-1.91)	2.03 (0.65-6.38)
Tertiary	1.42 (0.9-2.24)	3.27 (1.04-10.23)*	0.93 (0.39-2.22)	1.21 (0.65-2.24)	0.59 (0.29-1.23)	1.74 (0.47-6.44)
Job Status						
Employed	1.00	1.00	1.00	1.00	1.00	1.00
Unemployed	0.95 (0.52-1.76)	0.40 (0.09-1.84)	2.04 (0.85-4.87)	1.65 (0.83-3.30)	1.68 (0.69-4.12)	2.84 (1.01-7.97)*
Retired	1.1 (0.72-1.66)	0.64 (0.23-1.79)	1.07 (0.47-2.47)	0.78 (0.45-1.36)	0.98 (0.47-2.05)	1.49 (0.35-6.34)
Student, domestic, compulsoryservice	1.11 (0.79-1.57)	0.54 (0.21-1.34)	1.10 (0.54-2.25)	1.11 (0.71-1.75)	1.82 (0.97-3.40)	2.50 (1.20-5.24)*
Permanently disabled and other inactive status	0.72 (0.38-1.39)	0.82 (0.18-3.73)	1.06 (0.44-2.59)	1.05 (0.55-2.02)	1.11 (0.50-2.48)	1.83 (0.61-5.46)
Social support (Low)						
Low	1.00	1.00	1.00	1.00	1.00	1.00
Moderate	1.17 (0.67-2.04)	0.59 (0.22-1.56)	0.48 (0.22-1.06)	0.43 (0.24-0.78)**	0.35 (0.17-0.72)**	0.19 (0.08-0.44)***
High	0.74 (0.43-1.3)	0.47 (0.18-1.26)	0.35 (0.16-0.77)**	0.40 (0.22-0.72)**	0.30 (0.15-0.60)**	0.07 (0.03-0.16)***
Household income						

Below 1st quintile (Lowest)	1.00	1.00	1.00	1.00	1.00	1.00
Between 1st & 2nd quintile	0.87 (0.59-1.28)	0.75 (0.32-1.75)	0.38 (0.18-0.78)**	0.79 (0.51-1.22)	0.50 (0.27-0.94)*	0.55 (0.23-1.29)
Between 2nd & 3rd quintile	1.05 (0.73-1.52)	0.20 (0.07-0.58)**	0.68 (0.37-1.24)	0.79 (0.51-1.23)	0.76 (0.44-1.32)	0.87 (0.39-1.96)
Between 3rd & 4th quintile	1.19 (0.81-1.76)	0.37 (0.12-1.12)	0.41 (0.19-0.87)*	0.48 (0.28-0.81)**	0.32 (0.16-0.67)**	0.52 (0.19-1.42)
Between 4th & 5th quintile (Highest)	0.97 (0.65-1.44)	0.16 (0.04-0.61)**	0.39 (0.17-0.87)*	0.30 (0.17-0.54)***	0.35 (0.17-0.75)**	0.20 (0.06-0.71)*
Income not reported	0.85 (0.60-1.21)	0.52 (0.24-1.14)	0.63 (0.36-1.12)	0.64 (0.42-0.97)*	0.69 (0.41-1.19)	0.80 (0.38-1.72)
Informal Carer						
No	1.00	1.00	1.00	1.00	1.00	1.00
Yes	1.36 (1.06-1.75)*	1.08 (0.55-2.09)	1.37 (0.87-2.15)	1.07 (0.75-1.52)	1.07 (0.69-1.66)	0.63 (0.31-1.28)
BMI						
Underweight or Normal	1.00	1.00	1.00	1.00	1.00	1.00
Overweight	0.83 (0.66-1.06)	0.84 (0.44-1.63)	0.53 (0.31-0.90)*	1.25 (0.91-1.72)	1.10 (0.69-1.74)	2.26 (1.20-4.24)*
Obese	0.98 (0.74-1.3)	1.41 (0.77-2.59)	0.98 (0.58-1.65)	1.32 (0.91-1.90)	1.57 (0.95-2.57)	1.61 (0.81-3.21)
Smoker (No)						
No	1.00	1.00	1.00	1.00	1.00	1.00
Occasionally	1.51 (0.99-2.29)	1.92 (0.85-4.31)	2.24 (1.13-4.45)*	0.80 (0.43-1.48)	0.31 (0.09-1.06)	0.27 (0.06-1.24)
Daily	0.95 (0.71-1.27)	0.91 (0.43-1.9)	1.31 (0.81-2.12)	1.72 (1.25-2.38)**	1.04 (0.66-1.64)	2.04 (1.13-3.69)*
Alcohol consumption						
Never	1.00	1.00	1.00	1.00	1.00	1.00
Ex-drinkers	1.30 (0.87-1.94)	0.80 (0.32-1.97)	1.57 (0.78-3.19)	1.09 (0.67-1.77)	1.19 (0.6-2.36)	1.77 (0.74-4.21)
Irregularly	1.05 (0.71-1.56)	1.11 (0.46-2.65)	1.18 (0.57-2.42)	0.81 (0.5-1.33)	1.27 (0.69-2.37)	1.43 (0.57-3.61)
Regularly	1.16 (0.87-1.55)	0.54 (0.24-1.19)	1.00 (0.59-1.68)	0.60 (0.42-0.87)**	0.86 (0.52-1.41)	1.42 (0.69-2.93)
Everyday	1.07 (0.79-1.45)	0.70 (0.3-1.63)	0.94 (0.51-1.74)	0.78 (0.53-1.17)	0.91 (0.53-1.57)	1.18 (0.52-2.67)
Self-assessed health						
Good/very good	1.00	1.00	1.00	1.00	1.00	1.00
Fair	1.10 (0.85-1.44)	3.5 (1.88-6.53)***	1.41 (0.83-2.4)	1.52 (1.07-2.15)*	0.88 (0.55-1.41)	1.36 (0.68-2.73)
Bad/Very bad	1.86 (1.25-2.79)**	7.88 (3.71-16.73)***	3.76 (1.89-7.48)***	2.66 (1.61-4.39)***	1.67 (0.85-3.29)	2.40 (0.99-5.84)
Chronic disease (No)						
No	1.00	1.00	1.00	1.00	1.00	1.00
Yes	1.45 (1.14-1.84)**	1.09 (0.53-2.22)	0.77 (0.46-1.29)	0.90 (0.65-1.26)	0.75 (0.48-1.18)	2.04 (0.96-4.35)
Limitations in activities due to health problems						
No limitations	1.00	1.00	1.00	1.00	1.00	1.00
Limited/severely limited	1.56 (1.23-1.97)***	1.30 (0.72-2.35)	1.24 (0.74-2.10)	1.11 (0.80-1.55)	1.56 (0.99-2.45)	2.17 (1.08-4.35)*
Canton						
Esch zur Alzette	1.00	1.00	1.00	1.00	1.00	1.00
Capellen	1.12 (0.78-1.60)	1.17 (0.34-4.01)	1.12 (0.54-2.33)	1.09 (0.67-1.78)	1.33 (0.7-2.52)	1.50 (0.62-3.68)
Clervaux	0.81 (0.45-1.46)	3.63 (1.14-11.52)*	0.96 (0.31-2.92)	0.64 (0.27-1.48)	1.40 (0.56-3.49)	0.29 (0.05-1.68)

Diekirch	0.94 (0.54-1.62)	3.92 (1.27-12.04)*	2.04 (0.89-4.67)	1.17 (0.59-2.33)	2.06 (0.91-4.68)	1.58 (0.41-6.13)
Echternach	1.22 (0.64-2.31)	2.63 (0.64-10.84)	1.81 (0.59-5.60)	1.21 (0.59-2.46)	1.20 (0.38-3.81)	0.64 (0.06-6.66)
Grevenmacher	1.38 (0.89-2.16)	2.27 (0.77-6.66)	1.07 (0.42-2.72)	1.47 (0.83-2.59)	2.12 (1.03-4.35)*	1.53 (0.48-4.86)
Luxembourg	1.17 (0.90-1.53)	1.48 (0.68-3.23)	0.77 (0.46-1.31)	0.94 (0.66-1.35)	1.01 (0.60-1.68)	1.60 (0.83-3.10)
Mersch	1.75 (1.13-2.72)*	0.88 (0.18-4.39)	1.65 (0.71-3.85)	1.00 (0.51-1.92)	2.04 (1.00-4.16)	0.51 (0.10-2.54)
Redange	0.96 (0.56-1.63)	1.37 (0.25-7.53)	1.31 (0.53-3.28)	0.53 (0.22-1.30)	0.60 (0.18-2.03)	0.67 (0.13-3.34)
Remich	1.02 (0.57-1.84)	3.70 (1.1-12.5)*	0.66 (0.21-2.06)	0.85 (0.41-1.73)	1.30 (0.53-3.15)	3.64 (1.07-12.36)*
Vianden	0.49 (0.12-1.90)	6.99 (0.65-75.66)	0.69 (0.12-3.86)	0.91 (0.27-3.06)	2.37 (0.77-7.31)	1.69 (0.28-10.20)
Wiltz	0.57 (0.27-1.21)	1.44 (0.28-7.35)	1.74 (0.44-6.81)	0.98 (0.42-2.27)	1.43 (0.38-5.37)	0.93 (0.16-5.38)
Constant	0.22 (0.09-0.51)**	0.12 (0.02-0.73)*	0.07 (0.01-0.30)***	0.28 (0.10-0.80)*	0.25 (0.08-0.84)*	0.02 (0.00-0.11)***

Note: Estimates are weighted for age, sex and district of residence. ***p < 0.001.**p < 0.01.*p < 0.05. Data are odds ratios adjusted for explanatory variables (95% confidence intervals)

For peer review only

STROBE Statement—Checklist of items that should be included in reports of ***cross-sectional studies***

	Item No	Recommendation	Page No
**Title and abstract**	1	(*a*) Indicate the study's design with a commonly used term in the title or the abstract	1
		(*b*) Provide in the abstract an informative and balanced summary of what was done and what was found	2
**Introduction**			
Background/rationale	2	Explain the scientific background and rationale for the investigation being reported	4-7
Objectives	3	State specific objectives, including any prespecified hypotheses	7
**Methods**			
Study design	4	Present key elements of study design early in the paper	7
Setting	5	Describe the setting, locations, and relevant dates, including periods of recruitment, exposure, follow-up, and data collection	7-8
Participants	6	(*a*) Give the eligibility criteria, and the sources and methods of selection of participants	7
Variables	7	Clearly define all outcomes, exposures, predictors, potential confounders, and effect modifiers. Give diagnostic criteria, if applicable	8-9
Data sources/ measurement	8*	For each variable of interest, give sources of data and details of methods of assessment (measurement). Describe comparability of assessment methods if there is more than one group	8-9
Bias	9	Describe any efforts to address potential sources of bias	9
Study size	10	Explain how the study size was arrived at	8-9
Quantitative variables	11	Explain how quantitative variables were handled in the analyses. If applicable, describe which groupings were chosen and why	8-9
Statistical methods	12	(*a*) Describe all statistical methods, including those used to control for confounding	9
		(*b*) Describe any methods used to examine subgroups and interactions	NA
		(*c*) Explain how missing data were addressed	9
		(*d*) If applicable, describe analytical methods taking account of sampling strategy	9
		(*e*) Describe any sensitivity analyses	9
**Results**			
Participants	13*	(a) Report numbers of individuals at each stage of study—eg numbers potentially eligible, examined for eligibility, confirmed eligible, included in the study, completing follow-up, and analysed	8
		(b) Give reasons for non-participation at each stage	8
		(c) Consider use of a flow diagram	
Descriptive data	14*	(a) Give characteristics of study participants (eg demographic, clinical, social) and information on exposures and potential confounders	10, 24-25
		(b) Indicate number of participants with missing data for each variable of interest	Tables 1, A1 and A2

For peer review only

Outcome data	15*	Report numbers of outcome events or summary measures	10, 23
Main results	16	(*a*) Give unadjusted estimates and, if applicable, confounder-adjusted estimates and their precision (eg, 95% confidence interval). Make clear which confounders were adjusted for and why they were included	10-11, 28-31
		(*b*) Report category boundaries when continuous variables were categorized	8-9
		(*c*) If relevant, consider translating estimates of relative risk into absolute risk for a meaningful time period	N/A
Other analyses	17	Report other analyses done—eg analyses of subgroups and interactions, and sensitivity analyses	7 and Table A4
**Discussion**			
Key results	18	Summarise key results with reference to study objectives	11-12
Limitations	19	Discuss limitations of the study, taking into account sources of potential bias or imprecision. Discuss both direction and magnitude of any potential bias	13-14
Interpretation	20	Give a cautious overall interpretation of results considering objectives, limitations, multiplicity of analyses, results from similar studies, and other relevant evidence	12-16
Generalisability	21	Discuss the generalisability (external validity) of the study results	11-14
**Other information**			
Funding	22	Give the source of funding and the role of the funders for the present study and, if applicable, for the original study on which the present article is based	14

*Give information separately for exposed and unexposed groups.

Note: An Explanation and Elaboration article discusses each checklist item and gives methodological background and published examples of transparent reporting. The STROBE checklist is best used in conjunction with this article (freely available on the Web sites of PLoS Medicine at http://www.plosmedicine.org/, Annals of Internal Medicine at http://www.annals.org/, and Epidemiology at http://www.epidem.com/). Information on the STROBE Initiative is available at www.strobe-statement.org.

For peer review only
